# An Investigation of Electrocatalytic CO_2_ Reduction Using a Manganese Tricarbonyl Biquinoline Complex

**DOI:** 10.3389/fchem.2019.00628

**Published:** 2019-09-24

**Authors:** Meaghan McKinnon, Veronika Belkina, Ken T. Ngo, Mehmed Z. Ertem, David C. Grills, Jonathan Rochford

**Affiliations:** ^1^Department of Chemistry, University of Massachusetts Boston, Boston, MA, United States; ^2^Chemistry Division, Brookhaven National Laboratory, Upton, NY, United States

**Keywords:** carbon dioxide reduction, electrocatalysis, manganese, carbon monoxide, hydrogen evolution, computational modeling

## Abstract

The subject of this study [*fac*-Mn(bqn)(CO)_3_(CH_3_CN)]^+^ (bqn = 2,2′-biquinoline), is of particular interest because the bqn ligand exhibits both steric and electronic influence over the fundamental redox properties of the complex and, consequently, its related catalytic properties with respect to the activation of CO_2_. While not a particularly efficient catalyst for CO_2_ to CO conversion, *in-situ* generation and activity measurements of the [*fac*-Mn(bqn)(CO)_3_]^−^ active catalyst allows for a better understanding of ligand design at the Mn center. By making direct comparisons to the related 2,2′-bipyridyl (bpy), 1,10-phenanthroline (phen), and 2,9-dimethyl-1,10-phenanthroline (dmphen) ligands via a combination of voltammetry, infrared spectroelectrochemistry, controlled potential electrolysis and computational analysis, the role of steric vs. electronic influences on the nucleophilicity of Mn-based CO_2_ reduction electrocatalysts is discussed.

## Introduction

The catalytic reduction of CO_2_ into useful C-1 chemical feedstocks offers one potential strategy to develop a carbon-neutral alternative to our current dependence on fossil fuels. A major challenge for the catalysis community is to develop molecular catalysts capable of coupling electrochemical reduction with protonation of the CO_2_ substrate toward useful C-1 products. One such example is the proton-coupled two-electron reduction of CO_2_ to CO, which comes at a cost of just −0.52 V vs. SHE in water at pH 7 (Equation 1) (Arakawa et al., 2001), while in dry acetonitrile at pH 0 (Equation 2), the standard electrode potential for this reaction has been estimated, using two different thermodynamic cycles, as −0.13 V vs. Fc^+/0^ (Matsubara et al., 2015) or −0.12 V vs. Fc^+/0^ (Pegis et al., 2015).

(1) $$\begin{array}{r} {\text{CO}}_{2\left(\text{g}\right)}+{2{\text{H}}^{+}}_{\left(\text{aq}\right)}+{2\text{e}}^{-}⇋{\text{CO}}_{\left(\text{g}\right)}+{\text{H}}_{2}{\text{O}}_{\left(\text{l}\right)}{\text{E}}^{0}=-0.52\text{ V vs}.\text{ SHE }\left(\text{pH }7\right) \end{array}$$

(2) $$\begin{array}{r} \text{C}{\text{O}}_{2\left(\text{g}\right)}+{2{\text{H}}^{+}}_{\left(\text{CH}3\text{CN}\right)}+2{\text{e}}^{-}⇋\text{C}{\text{O}}_{\left(\text{g}\right)}+{\text{H}}_{2}{\text{O}}_{\left(\text{CH}3\text{CN}\right)}\;{\text{E}}^{0}=-0.13\text{ V vs }.\text{ F}{\text{c}}^{+/0}\left(pH\;0\right) \end{array}$$

This seemingly simple reaction is limited by slow kinetic parameters, however, which demands an appropriate catalyst to access a kinetically efficient pathway for CO_2_ utilization – albeit at the cost of an electrochemical overpotential (η) (Appel and Helm, 2014). Of the homogeneous transition metal-based molecular catalysts used in this field (Francke et al., 2018; Sinopoli et al., 2018; Stanbury et al., 2018), manganese(I) polypyridyl tricarbonyl catalysts of the type [*fac*-Mn^I^(N^∧^N)(CO)_3_X]^n^ (where N^∧^N = polypyridyl ligand, X = Br^−^ (*n* = 0) or CH_3_CN (*n* = +1)), and their analogs, have been of keen interest due to their high selectivity, low cost, and low overpotential (Grills et al., 2018). Recently we have reported on tuning both the inner coordination sphere (McKinnon et al., 2019) and second coordination sphere (Ngo et al., 2017) of the ligand to optimize catalytic efficiency and selectivity for CO formation, notably against the competitive two-electron two-proton coupled redox transformations of CO_2_ to HCO_2_H and H^+^ to H_2_. More subtle modification on the periphery of the bpy ligand is also known to strongly influence the reduction potentials observed for these catalysts. This is evident, for example, with the inductive electron donating influence of the dtbpy (dtbpy = 4,4′-^t^Bu_2_-bpy) ligand, which shifts the reduction potential 0.11 V more negative relative to the simple bpy analog (Smieja et al., 2013). Similarly, while maintaining the same inner coordination sphere of [*fac-*Mn(N^∧^N)(CO)_3_L]^n^ catalysts but introducing redox non-innocence on the ligand backbone, a recent report on [*fac-*MnBr(phen-dione)(CO)_3_] (phen-dione = 1,10-phenanthroline-5,6-dione) demonstrated vastly different electrochemistry, maintaining a high selectivity for CO evolution, compared to the analogous bpy and phen (phen = 1,10-phenanthroline) complexes, notwithstanding significant ligand-based redox activity at the dione functional group to generate a bis-carboxylate phenanthroline intermediate species (Stanbury et al., 2017).

**Figure 1:**
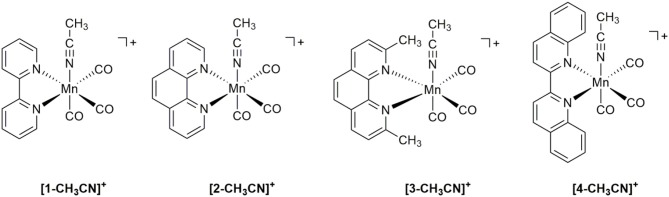
Molecular structures of complexes investigated.

In the current study we have focused on a rather simple modification of the polypyridyl ligand, but one which allows us to directly investigate both steric and electronic influences in the [*fac-*Mn(bqn)(CO)_3_(CH_3_CN)]^+^**([4-CH**_**3**_**CN]**^**+**^**)** pre-catalyst, where bqn = 2,2′-biquinoline (Figure 1). While fundamental in approach, a simple systematic extension of the π-conjugated system of the polypyridyl ligand has not yet been reported for Mn(I) CO_2_ reduction electrocatalysts. Four pre-catalysts of the general structure [*fac-*Mn(N^∧^N)(CO)_3_(CH_3_CN)]^+^ are here investigated using the benchmark 2,2′-bipyridyl (bpy) ligand **([1-CH**_**3**_**CN]**^**+**^**)** alongside the 1,10-phenanthroline (phen) **([2-CH**_**3**_**CN]**^**+**^**)**, 2,9-dimethyl-1,10-phenanthroline (dmphen) **([3-CH**_**3**_**CN]**^**+**^**)**, and aforementioned bqn ligands. Although the phen ligand does not have as extensive a π-conjugation as the bqn ligand, it serves a critical role in this study by bridging the gap between the sterically related dmphen and bqn ligands. Indeed, a direct comparison of the sterically and electronically expanded bqn ligand to the sterically related dmphen ligand probes the question of whether steric or electronic effects dominate in determining the redox, and ultimately catalytic, properties of this class of CO_2_ reduction electrocatalyst. As demonstrated below using a combination of IR-SEC and computational studies, the combined electronic and steric influences of the π-extended bqn ligand hinder formation of a **[4–4]**^**0**^ dimer, facilitating a concerted two-electron *ECE* mechanism for the generation of **[4]**^**−**^. However, this comes at the cost of a change in product selectivity for the **[4-CH**_**3**_**CN]**^**+**^ pre-catalyst to favor H_2_ evolution in the presence of excess TFE. Selective CO_2_ activation is observed for all catalysts here studied upon *in-situ* generation of a three-electron reduced [*fac-*Mn^(−1)^(N^∧^N^•−^)(CO)_3_]^2−^ active catalyst.

## Results and Discussion

### Synthesis and Structural Characterization

**Figure 2:**
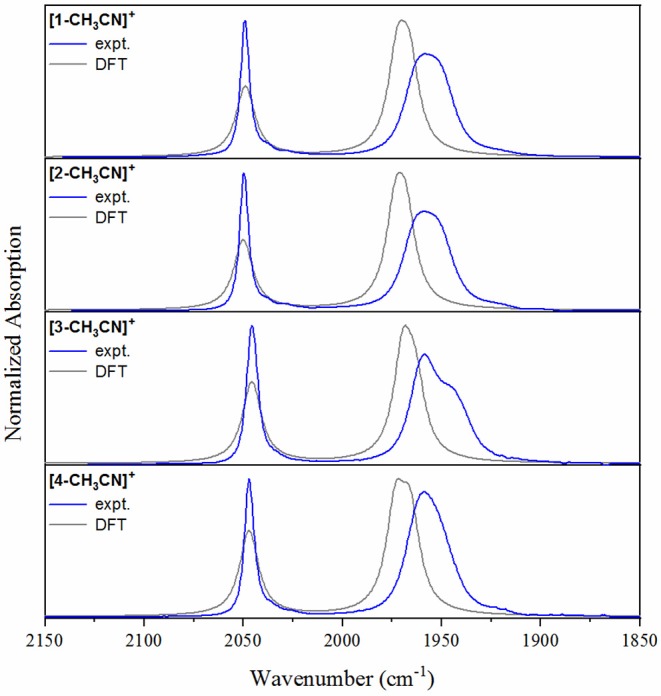
Experimental FTIR spectra (blue) of **[1-CH**_**3**_**CN]**^**+**^, **[2-CH**_**3**_**CN]**^**+**^, **[3-CH**_**3**_**CN]**^**+**^, and **[4-CH**_**3**_**CN]**^**+**^ recorded in acetonitrile displaying characteristic ν(CO) stretching modes. Density functional theory-computed IR spectra (gray) are included for comparison (empirically-derived frequency scaling factor = 0.9633, FWHM = 12 cm^−1^).

Initial metathesis of [MnBr(CO)_5_] with silver triflate was completed as a first step to produce the [Mn(CO)_5_(OTf)] intermediate (Scheiring et al., 2000), allowing for the subsequent straightforward isolation of pure [*fac-*Mn^I^(N^∧^N)(CO)_3_(OTf)] products. Microwave reflux of [Mn(CO)_5_(OTf)] with one equivalent of the appropriate ligand in tetrahydrofuran afforded the pale yellow [*fac-*Mn(bpy)(CO)_3_(OTf)] (**1-OTf**), [*fac-*Mn(phen)(CO)_3_(OTf)] (**2-OTf**), and [*fac-*Mn(dmphen)(CO)_3_(OTf)] (**3-OTf**) products, and the red-orange [*fac-*Mn(bqn)(CO)_3_(OTf)] (**4-OTf**) solid, in quantitative yield following precipitation in excess diethyl ether. Each product was satisfactorily characterized by ^1^H NMR and FTIR spectroscopies as well as elemental analysis. The *facial (fac)* arrangement of the Mn(CO)_3_ core structure in each complex was confirmed by FTIR spectroscopy, where characteristic ν(CO) vibrational stretching modes for each of the solvated **[1-CH**_**3**_**CN]**^**+**^, **[2-CH**_**3**_**CN]**^**+**^, **[3-CH**_**3**_**CN]**^**+**^ and **[4-CH**_**3**_**CN]**^**+**^ complexes in neat acetonitrile provides a very useful comparison of structural and electronic properties of all four complexes using a basic knowledge of point-group symmetry and Mn(*d*π)-CO(π^*^) back-bonding, respectively (**Table 2**). The bpy and phen complexes, **[1-CH**_**3**_**CN]**^**+**^ and **[2-CH**_**3**_**CN]**^**+**^, each exhibit *pseudo*-*C*_3v_ point group symmetry with identical FTIR spectra composed of a sharp, symmetric ν(CO) stretching mode at 2,050 cm^−1^ and a second broad, lower frequency asymmetric stretching mode at 1,958 cm^−1^. Consistent with voltammetry and computational analysis presented below, the identical ν(CO) stretching modes illustrates the negligible electronic influence of the π-extended phen ligand in comparison to the bpy system. This observation is consistent with related reports of the analogous bromide complexes (Kurtz et al., 2015; Stanbury et al., 2017; Tignor et al., 2018). In contrast, the dmphen complex **[3-CH**_**3**_**CN]**^**+**^ exhibits a descent in symmetry to *pseudo-C*_s_, evident in a breaking of degeneracy for its lower frequency asymmetric ν(CO) stretching modes, which occur at 1,959 and 1,944(sh) cm^−1^. This suggests a sterically induced distortion of the *fac*-Mn(CO)_3_ core, likely by the 2,10-dimethyl substituents of the dmphen ligand. Furthermore, the higher frequency symmetric ν(CO) stretching mode of **[3-CH**_**3**_**CN]**^**+**^ exhibits a 4 cm^−1^ shift to lower frequency at 2,046 cm^−1^, consistent with an inductive electron-donating influence of the two methyl substituents at dmphen, likely due to increased Mn(*d*π) → CO(π^*^) back-bonding. Interestingly, the bqn complex, **[4-CH**_**3**_**CN]**^**+**^ exhibits an FTIR spectral profile with ν(CO) = 2,047 and 1,959 cm^−1^, indicative again of *pseudo-C*_3v_ symmetry, similar to **[1-CH**_**3**_**CN]**^**+**^ and **[2-CH**_**3**_**CN]**^**+**^. However, with the high frequency symmetric ν(CO) stretching mode occurring at 2,047 cm^−1^, this suggests that the inductive electron-donating influence of the bqn ligand is similar to that of the dmphen ligand in **[3-CH**_**3**_**CN]**^**+**^. Experimental FTIR spectral profiles are consistent with calculated spectra, as illustrated in Figure 2.

### Cyclic Voltammetry Under Non-catalytic Conditions

**Figure 3:**
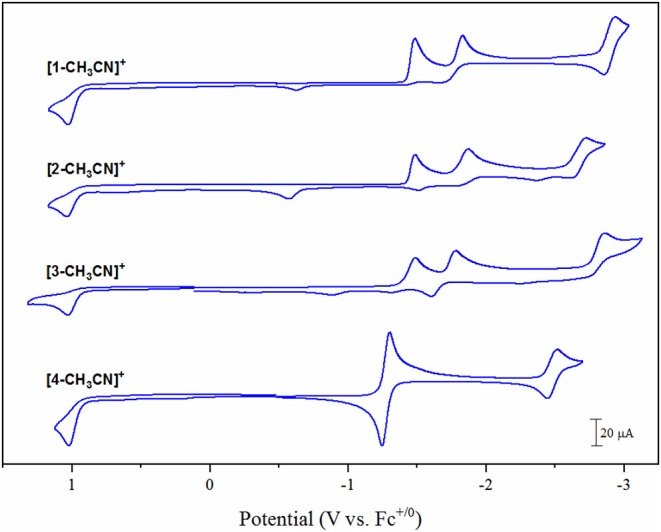
Cyclic voltammograms of **[1-CH**_**3**_**CN]**^**+**^, **[2-CH**_**3**_**CN]**^**+**^, **[3-CH**_**3**_**CN]**^**+**^ and **[4-CH**_**3**_**CN]**^**+**^ recorded in 0.1 M [Bu_4_N][PF_6_] acetonitrile supporting electrolyte at a glassy carbon working electrode with a scan rate of υ = 0.1 V s^−1^ under 1 atmosphere of argon.

Electrochemical characterization of each complex was carried out under inert conditions (1 atmosphere of argon), in the absence of auxiliary Brønsted acid, prior to screening for catalytic activity. Each complex displays an irreversible Mn(II/I) oxidation event within 20 mV of each other in the range of +1.02 to +1.04 V vs. the ferricenium/ferrocence (Fc^+/0^) pseudo-reference (Figure 3, Table 1). This suggests that there is little difference in electron density between each Mn-center upon varying the polypyridyl ligand across the four complexes. The very subtle differences observed in the *v*(CO) stretching modes by FTIR spectroscopy are unlikely to be reproduced by cyclic voltammetry. Furthermore, voltammetry data is more complex due to the *in-situ* generation of the Mn(II) oxidation state. We will focus on the reduction properties of these complexes forthwith due to their greater relevance toward our subsequent catalytic studies. The electrochemical properties of **[1-CH**_**3**_**CN]**^**+**^ have been recently reported (Grills et al., 2018; McKinnon et al., 2019). Three sequential one-electron cathodic peaks are observed at *E*_pc_ = −1.48 V, −1.83 V and −2.94 V with only the third reduction exhibiting quasi-reversible behavior (*E*_1/2_ = −2.90 V, Δ*E*_p_ = 81 mV at υ = 0.1 V s^−1^) vs. Fc^+/0^. The phen complex, **[2-CH**_**3**_**CN]**^**+**^ exhibits a first one-electron irreversible reduction at *E*_pc_ = −1.48 V vs. Fc^+/0^, identical to that observed for **[1-CH**_**3**_**CN]**^**+**^, further supporting the electronic similarity of bpy and phen at least in the [*fac-*Mn(N^∧^N)(CO)_3_L]^n^ class of complexes (Tignor et al., 2018). This first reduction has been previously established (Grills et al., 2018) as ligand-based according to an electrochemical-chemical (*EC*) reaction scheme whereby, upon one-electron reduction of the bpy π^*^ orbital, rapid CH_3_CN dissociation occurs with a concurrent shift in radical character from the ligand to form the neutral five-coordinate 17-valence electron intermediate, [*fac-*Mn^0^(bpy)(CO)_3_] (**[1]**^**0**^). This metastable Mn(0) complex, in the absence of any steric hindrance (Sampson et al., 2014), rapidly forms the Mn^0^-Mn^0^ bound 18-valence electron [*fac-*Mn^0^(bpy)(CO)_3_]_2_ dimer, **[1–1]**^**0**^. While unequivocal evidence of [*fac-*Mn^0^(N^∧^N)(CO)_3_] dimer formation is presented below via infrared spectroelectrochemical (IR-SEC) studies, its oxidation is often evident in the reverse anodic scan in cyclic voltammetry, observed here at −0.61 V and −0.58 V for **[1–1]**^**0**^ and **[2–2]**^**0**^, respectively. Thus, the second reduction event for [*fac-*Mn(N^∧^N)(CO)_3_L]^n^ complexes is often attributed to an irreversible two-electron *EC* event whereby the dimer is reductively cleaved to generate two equivalents of the two-electron reduced [*fac-*Mn^0^(N^∧^N^•−^)(CO)_3_]^−^ anion, now established as the principal active catalyst for CO_2_ activation by this class of complex via the most common *reduction-first* pathway (Riplinger et al., 2014). Reduction of the phen based dimer, **[2–2]**^**0**^ is observed at *E*_pc_ = −1.86 V, suggesting that **[2]**^**−**^ is more nucleophilic than the benchmark bpy analog, **[1]**^**−**^ generated at the slightly more positive potential of *E*_pc_ = −1.83 V. Similar to its bpy analog, the native **[2-CH**_**3**_**CN]**^**+**^ precursor exhibits a quasi-reversible third reduction event at *E*_pc_ = −2.72 V (*E*_1/2_ = −2.66 V, Δ*E*_p_ = 114 mV at υ = 0.1 V s^−1^). This third quasi-reversible reduction has rarely been discussed in the literature but computational analysis here suggests that it is a predominantly ligand based reduction giving rise to a 19-electron [*fac-*Mn^0^(N^∧^N^2−^)(CO)_3_]^2−^ dianion. The related dmphen complex, **[3-CH**_**3**_**CN]**^**+**^ exhibits unexceptionally similar voltammetry in comparison to both its bpy and phen analogs. Its first reduction again involves a ligand based *EC* pathway leading to formation of the **[3–3]**^**0**^ dimer. This *EC* reaction is shifted negatively by 10 mV to *E*_pc_ = −1.49 V, relative to formation of both **[1–1]**^**0**^ and **[2–2]**^**0**^, consistent with the inductive donating character of the 2,9-dimethyl substituents observed by FTIR studies. Although there is weak evidence of dimer oxidation in the full scan voltammogram presented in Figure 3, reversing the scan after just one-electron reduction clearly demonstrates a significant dimer oxidation peak at *E*_pa_ = −0.65 V (Figure S5). Reductive cleavage of the **[3–3]**^**0**^ dimer to form the two-electron reduced five-coordinate [*fac-*Mn^0^(dmbpy^•−^)(CO)_3_]^−^ anion (**[3]**^**−**^), however, occurs at a more positive potential of −1.78 V. This observation suggests that the steric influence of dmphen slightly hinders electronic coupling between the Mn(0) and dmphen^•−^ radical centers in **[3]**^**−**^, rendering it slightly less nucleophilic as a result. The third reduction wave for **[3]**^**−/2−**^ is observed at −2.86 V and, in contrast to its bpy and phen analogs, is completely irreversible.

**Table 1:** Redox potentials recorded by cyclic voltammetry for **[1-CH**_**3**_**CN]**^**+**^, **[2-CH**_**3**_**CN]**^**+**^, **[3-CH**_**3**_**CN]**^**+**^, and **[4-CH**_**3**_**CN]**^**+**^ reported vs. the ferricenium/ferrocene (Fc^+/0^) pseudo reference.

	**Oxidation**		**Reduction**		
**[1-CH**_**3**_**CN]**^**+**^	+1.03^a^	−0.61^b^	−1.48^c^	−1.83^c^	−2.90^d^
**[2-CH**_**3**_**CN]**^**+**^	+1.04^a^	−0.58^b^	−1.48^c^	−1.86^c^	−2.66^d^
**[3-CH**_**3**_**CN]**^**+**^	+1.03^a^	−0.65^b^	−1.49^c^	−1.78^c^	−2.86^c^
**[4-CH**_**3**_**CN]**^**+**^	+1.02^a^		−1.28^e^	−2.48^d^	

a *E_pa_, irreversible one-electron oxidation*.

b *E_pa_, Mn^0^-Mn^0^ dimer oxidation*.

c *E_pc_, irreversible one-electron reduction*.

d *E_1/2_, quasi-reversible one-electron reduction*.

e *E_1/2_, reversible concerted two-electron reduction*.

Conditions: 1 mM sample concentration; 0.1 M [Bu_4_N][PF_6_] acetonitrile supporting electrolyte; 3 mm diameter glassy carbon working electrode; Pt wire counter electrode; Ag/AgPF_6_ acetonitrile non-aqueous reference electrode; υ = 0.1 V s^−1^.

Noticeably in Figure 3, the bqn complex, **[4-CH**_**3**_**CN]**^**+**^ exhibits a single, reversible, concerted two-electron reduction event at *E*_pc_ = −1.30 V (*E*_1/2_ = −1.28 V, Δ*E*_p_ = 53 mV at υ = 0.1 V s^−1^) followed by a single additional quasi-reversible one-electron reduction event at *E*_pc_ = −2.51 V (*E*_1/2_ = −2.48 V, Δ*E*_p_ = 71 mV at υ = 0.1 V s^−1^) vs. Fc^+/0^. Equally important here is the lack of any evidence for dimer oxidation in the reverse anodic scan of **[4-CH**_**3**_**CN]**^**+**^. This electrochemical behavior, at least in its peak profile, is uncannily similar to previously reported bulky 6,6′-substituted bpy ligands investigated at the identical [*fac-*Mn^0^(N^∧^N)(CO)_3_(CH_3_CN)]^+^ center (Sampson et al., 2014; Ngo et al., 2017). Mn^0^-Mn^0^ formation is known to favor a staggered structure of both bpy ligands in **[1–1]**^**0**^ (Machan et al., 2014), and thus far only the inclusion of steric bulk orthogonal to the plane of the polypyridyl ligand has been demonstrated to prevent dimer formation (Sampson et al., 2014; Ngo et al., 2017). Thus, it is appropriate here to question whether the redox behavior exhibited by **[4-CH**_**3**_**CN]**^**+**^ is in fact due to an electronic influence of this relatively electron deficient ligand, or rather, alternatively is the result of a steric influence of the bqn ligand. The bqn ligand is, after all, π-extended from the same 6,6′-positions as previously reported 6,6′-orthoganol-sterically bulky bpy stystems, albeit here non-orthogonal to, but in the same plane as, the core bpy structure. This question is addressed explicitly via computational analysis presented below.

### Infrared Spectroelectrochemistry (IR-SEC)

**Figure 4:**
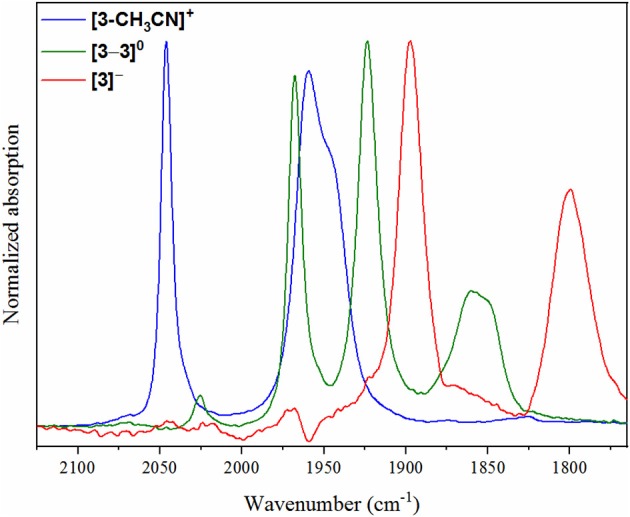
IR-SEC spectra recorded on **[3-CH**_**3**_**CN]**^**+**^ at the resting potential (blue), upon one-electron reduction (green) and upon two-electron reduction (red).

To gain structural insight into electrochemical activation of these pre-catalysts, IR-SEC was carried out in 0.1 M [Bu_4_N][PF_6_] acetonitrile supporting electrolyte under 1 atmosphere of argon (Table 2). IR-SEC is a powerful investigative tool that takes advantage of the structural specificity of IR spectroscopy for transition metal carbonyl complexes and the ability to prepare *in-situ* catalytic intermediates simply by gradually stepping the potential of the working electrode (Kaim and Fiedler, 2009; Machan et al., 2014). At open-circuit potential, i.e., resting potential of the native pre-catalyst, **[1-CH**_**3**_**CN]**^**+**^ and **[2-CH**_**3**_**CN]**^**+**^ both display identical ν(CO) stretching modes as presented earlier (Figure 2), confirming negligible influence of the 0.1 M [Bu_4_N][PF_6_] electrolyte on their FTIR spectra. When the potential was biased beyond the first-reduction at −1.60 V vs. Fc^+/0^ for both **[1-CH**_**3**_**CN]**^**+**^ and **[2-CH**_**3**_**CN]**^**+**^, a progression was observed where the native ν(CO) stretching modes diminished while four unique but related ν(CO) stretching modes grew in concurrently at 1,857, 1,879, 1,933, and 1,976 cm^−1^ from **[1-CH**_**3**_**CN]**^**+**^ (Figure S10) and at 1,857, 1,880, 1,935, and 1,977 cm^−1^ from **[2-CH**_**3**_**CN]**^**+**^ (Figure S11). These data are consistent with an earlier report of the **[1–1]**^**0**^ dimer (Hartl et al., 1995), and the peak profile associated with related Mn^0^-Mn^0^ dimers previously observed via IR-SEC (Grills et al., 2018), and they are also consistent with our computational frequency analysis. A similar assignment is made for **[2–2]**^**0**^ which exhibits almost identical ν(CO) stretching frequencies (vide supra). Upon biasing the working electrode potential further negative (−2.00 V vs. Fc^+/0^) beyond the second Mn^0^-Mn^0^/Mn^−^ based reduction for both **[1-CH**_**3**_**CN]**^**+**^ and **[2-CH**_**3**_**CN]**^**+**^, loss of the four dimer stretches was observed (Figures S10, S11) with concurrent growth of two new ν(CO) stretching modes at lower wavenumber, corresponding to the five-coordinate two-electron reduced [*fac-*Mn^0^(N^∧^N^•−^)(CO)_3_]^−^ active catalysts, **[1]**^**−**^ [ν(CO) = 1,811 and 1,911 cm^−1^] and **[2]**^**−**^ [ν(CO) = 1,813 and 1,923 cm^−1^]. Consistent with its comparable voltammetry behavior, the dmphen based complex, **[3-CH**_**3**_**CN]**^**+**^ exhibits a similar IR-SEC transition to the two-electron reduced active catalyst, **[3]**^**−**^ [ν(CO) = 1,799 and 1,897 cm^−1^] via the **[3–3]**^**0**^ dimer intermediate [ν(CO) = 1,849(sh), 1,860, 1,923, and 1,968 cm^−1^] as illustrated in Figure 4. Although clean transformation of **[3-CH**_**3**_**CN]**^**+**^ to **[3]**^**−**^ was observed upon direct electrolysis at −2.00 V, it is worth noting that selective electrolysis to the intermediate **[3–3]**^**0**^ dimer species did exhibit evidence of minor decomposition to an unidentified side-product [ν(CO) = 1,919 and 2,026 cm^−1^] with concurrent growth of a broad weak infrared absorption consistent with free CO (Figure S13). This is consistent with an earlier report of poor stability of the simpler phen-based dimer, **[2–2]**^**0**^ (Stanbury et al., 2017).

**Table 2:** ν(CO) infrared stretches for **[1-CH**_**3**_**CN]**^**+**^, **[2-CH**_**3**_**CN]**^**+**^, **[3-CH**_**3**_**CN]**^**+**^, and **[4-CH**_**3**_**CN]**^**+**^ precatalysts, and their one-electron reduced, dimeric, and two-electron reduced derivatives, obtained by solution phase FTIR and IR-SEC spectroscopy.

	***v*(CO), cm^**−1**^**
**[1-CH_**3**_**CN]**^+^^a^**	1,958; 2,050
**[1–1]^0^^b^**	1,857; 1,879; 1,933; 1,976
**[1]^–^^b^**	1,811; 1,911
**[2-CH_**3**_**CN]**^+^^a^**	1,958; 2,050
**[2–2]^0^^b^**	1,857; 1,880; 1,935; 1,977
**[2]^–^^b^**	1,813; 1,923
**[3-CH_**3**_**CN]**^+^^a^**	1,944(sh); 1,959; 2,046
**[3–3]^0^^b^**	1,849(sh); 1,860; 1,923; 1,968
**[3]^–^^b^**	1,799; 1,897
**[4-CH_**3**_**CN]**^+^^a^**	1,959; 2,047
**[4]^–^^b^**	1,828; 1,925

a Recorded in neat CH_3_CN.

b *Recorded in 0.1 M [Bu_4_N][PF_6_] acetonitrile electrolyte*.

**Figure 5:**
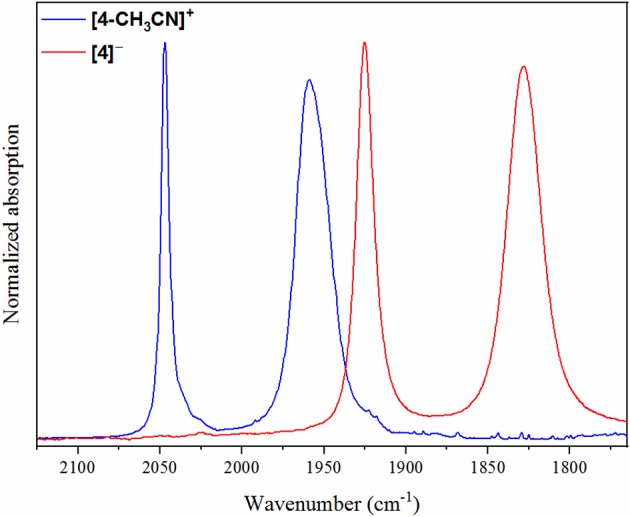
IR-SEC spectra recorded on **[4-CH**_**3**_**CN]**^**+**^ at the resting potential (blue) and upon two-electron reduction (red).

As a concerted two-electron reduction event occurs for **[4-CH**_**3**_**CN]**^**+**^, the potential was set at −1.65 V vs. Fc^+/0^ for quantitative *in-situ* formation of the two-electron reduced product. Indeed, no evidence for an intermediate one-electron reduced monomer or the Mn^0^-Mn^0^ dimer was observed. Instead, consistent with the observed voltammetry (vide supra) only a transition to two lower-frequency stretching modes at ν(CO) = 1,828 and 1,925 cm^−1^ was observed, which are attributed to the two-electron reduced [*fac-*Mn^0^(bqn^•−^)(CO)_3_]^−^ species, **[4]**^**−**^ (Figure 5).

### Voltammetry Under 1 Atm CO_2_ in the Presence of 0.3 % H_2_O

**Figure 6:**
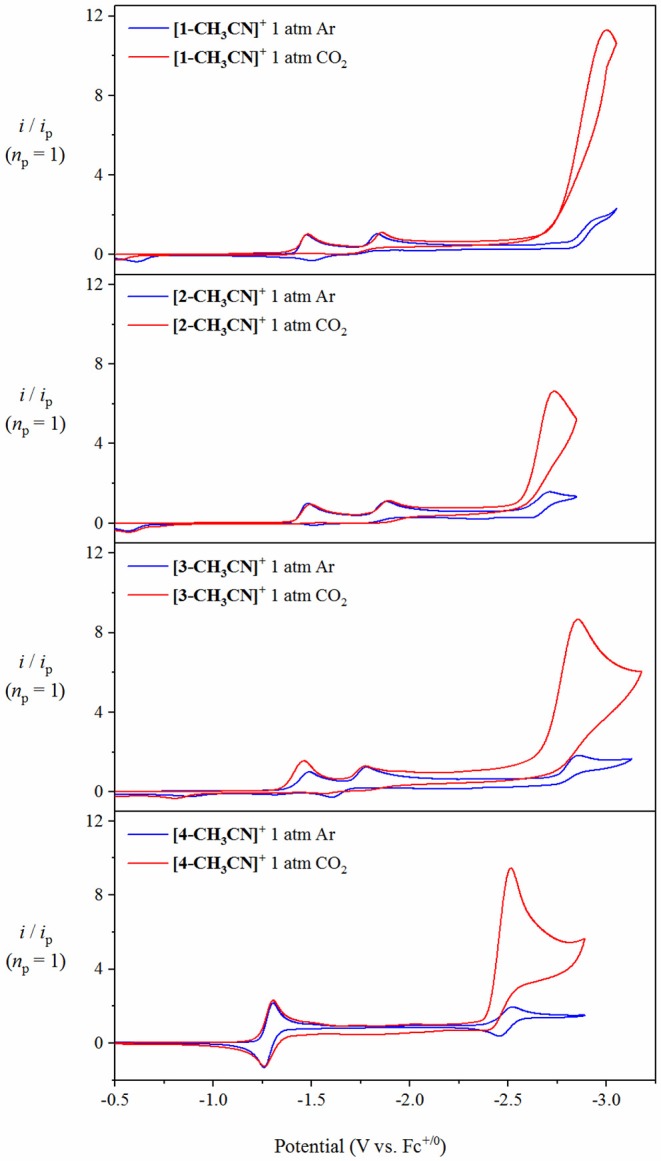
Cyclic voltammograms demonstrating catalytic activity of **[1-CH**_**3**_**CN]**^**+**^, **[2-CH**_**3**_**CN]**^**+**^, **[3-CH**_**3**_**CN]**^**+**^ and **[4-CH**_**3**_**CN]**^**+**^ under 1 atm CO_2_ (red) at υ = 0.1 V s^−1^ with 0.1 M [Bu_4_N][PF_6_] acetonitrile supporting electrolyte containing residual 0.17 M (0.3%) H_2_O as a Brønsted acid source. Cyclic voltammograms recorded under 1 atm of argon are also shown (blue). The current (y-axis) data are normalized with respect to the non-catalytic Faradaic response (*i*_p_).

Prior studies of Mn(I) polypyridyl based electrocatalysts for CO_2_ reduction have only reported catalytic current upon addition of excess weak Brønsted acid with, for example, 2.71 M (5%) addition of H_2_O (Bourrez et al., 2011). In contrast to their Re counterparts, which have been established for some time to promote CO_2_ reduction in the absence of a proton donor, Mn(I) polypyridyl based electrocatalysts typically require the presence of a proton donor to promote intermediate metallocarboxylic acid formation due to the poorer nucleophilicity of Mn vs. Re catalysts (Riplinger and Carter, 2015). Cyclic voltammograms for pre-catalysts **[1-CH**_**3**_**CN]**^**+**^, **[2-CH**_**3**_**CN]**^**+**^, **[3-CH**_**3**_**CN]**^**+**^ and **[4-CH**_**3**_**CN]**^**+**^ recorded under 1 atm CO_2_ at 0.1 V s^−1^ in 0.1 M [Bu_4_N][PF_6_] acetonitrile electrolyte exhibit clear evidence of catalytic behavior for each complex (Figure 6). Although excess Brønsted acid was not added in these experiments, the reagent grade acetonitrile that was used still contains 0.17 M (0.3 %) residual H_2_O, which was recently demonstrated to be sufficient to facilitate proton-coupled CO_2_ reduction to form CO (McKinnon et al., 2019). This observation is logical due to the relatively larger overpotential applied, especially as the active catalyst under these experimental conditions is predicted by computation (vide infra) to be the more nucleophilic three-electron reduced dianion.

Without knowledge of the *in-situ* pH of the electrolyte solution, the half-wave potential of the catalytic wave (*E*_cat/2_) can be used to make a relative comparison of overpotentials under these experimental conditions. As expected, π-extension with the bqn ligand in pre-catalyst **[4-CH**_**3**_**CN]**^**+**^ gives rise to a significant positive shift of catalytic current with *E*_cat/2_ = −2.42 V, whereas the bpy, phen and dmphen pre-catalysts exhibit *E*_cat/2_ = −2.85, −2.64, and −2.74 V, respectively. One difficulty encountered under these experimental conditions was in attempting to establish steady-state catalytic conditions with respect to the rate-limiting consumption of CO_2_ within the electrochemical double-layer. As discussed later, this became less problematic once excess Brønsted acid was added. As such, TOF_max_ could not be satisfactorily determined under these experimental conditions. Thus, the TOF value is reported for each catalyst in Table 3 using Equation 3 and the *i*_cat_*/i*_p_ ratio determined at a scan rate of 0.1 V s^−1^, to aid their side-by-side comparison,

(3) $$\begin{array}{r} \text{TOF}=0.1992\left(\frac{F\upsilon }{RT}\right)\left(\frac{n_{p}^{3}}{n_{cat}^{2}}\right){\left(\frac{i_{cat}}{i_{p}}\right)}^{2} \end{array}$$

where *F* is the Faraday constant (96,485 s A mol^−1^), υ is the scan rate (V s^−1^), *R* is the universal gas constant (8.3145 V A s K^−1^ mol^−1^), *T* is the temperature (K), *n*_p_ is the number of electrons involved in the non-catalytic Faradaic current response (responsible for the non-catalytic Faradaic current, *i*_p_, as described by the Randles-Sevcik equation (Bard and Faulkner, 2001), and *n*_cat_ is the number of electrons required for catalysis (two electrons for the reduction of CO_2_ to CO as shown in Equations 1 and 2). In our calculations of TOF, the first one-electron reduction wave (*n*_p_ = 1) was used for reference to determine the non-catalytic Faradaic current (*i*_p_) for **[1-CH**_**3**_**CN]**^**+**^, **[2-CH**_**3**_**CN]**^**+**^ and **[3-CH**_**3**_**CN]**^**+**^. In contrast, the first reduction wave of **[4-CH**_**3**_**CN]**^**+**^ exhibits a concerted two-electron event under non-catalytic conditions such that *n*_p_ = 2 at *E*_pc_ = −1.30 V. The significance of this distinction is important when determining TOF from cyclic voltammetry analysis using Equation 3 as the ratio of $\frac{n_{p}^{3}}{n_{cat}^{2}}$ can differ by a factor of 8 (1/4 vs. 8/4 for *n*_*p*_ = 1 or 2, respectively). However, this should, with ideal Randles-Sevcik behavior (*i*_p_ $\propto n_{p}^{3/2}$) of the two-electron reduction event, be completely offset by the 8 × smaller (*i*_cat_/*i*_p_)^2^ ratio when *n*_p_ = 2. Thus, in theory, kinetic analysis of **[4-CH**_**3**_**CN]**^**+**^ can be conducted using either the concerted two-electron reduction event at *E*_pc_ = −1.30 V (*n*_p_ = 2) or the subsequent one-electron reduction event at *E*_pc_ = −2.51 V (*n*_p_ = 1) for reference in determining *i*_cat_/*i*_p_. Using data recorded at υ=0.1 V s^−1^, Equation 3 results in different TOF values of 25 s^−1^ (*i*_cat_/*i*_p_ = 4.0, *n*_p_ = 2) or 14 s^−1^ (*i*_cat_/*i*_p_ =8.5, *n*_p_ = 1), indicating non-ideal behavior of the concerted two-electron reduction event, which is unsurprising considering this is an *ECE* mechanism and not a pure two-electron concerted *EE* mechanism. Scan rate analysis of this two-electron reduction event for **[4-CH**_**3**_**CN]**^**+**^ clearly demonstrates an increase in peak separation between its cathodic and anodic waves (Figure S9). More importantly, the 2.83-fold increase in current for a two-electron event, predicted by the Randles-Sevcik equation (*i*_p_ $\propto n_{p}^{3/2}$) assuming similar diffusion coefficients for each redox state, is in fact found to be significantly smaller (2.13-fold) when we compare the *i*_p_ values of **[4-CH**_**3**_**CN]**^**+**^ at *E*_pc_ = −1.30 (*n*_p_ = 2) and *E*_pc_ = −2.51 V (*n*_p_ = 1). The TOF value of 25 s^−1^ (*i*_cat_/*i*_p_ = 4.0, *n*_p_ = 2) is therefore an overestimate of this catalyst's efficiency, and the value of TOF=14 s^−1^ is henceforth quoted for **[4-CH**_**3**_**CN]**^**+**^ (*i*_cat_/*i*_p_ = 8.5, *n*_p_ = 1) recorded at υ = 0.1 V s^−1^. In summary, the benchmark bpy-based pre-catalyst, **[1-CH**_**3**_**CN]**^**+**^ (*i*_cat_/*i*_p_ = 11.3) exhibits the highest TOF in this study of 25 s^−1^ at υ=0.1 V s^−1^, followed closely by **[3-CH**_**3**_**CN]**^**+**^ at 22 s^−1^ (*i*_cat_/*i*_p_ = 10.7), the bqn-based system **[4-CH**_**3**_**CN]**^**+**^ at 14 s^−1^ (*i*_cat_/*i*_p_ = 8.5) and finally **[2-CH**_**3**_**CN]**^**+**^ at just 11 s^−1^ (*i*_cat_/*i*_p_ = 7.4).

**Table 3:** Summary of electrocatalysis data derived from voltammetry experiments, in the absence of TFE and at optimum TFE concentrations.

	**[1-CH**_****3****_**CN]**^****+****^		**[2-CH**_****3****_**CN]**^****+****^		**[3-CH**_****3****_**CN]**^****+****^		**[4-CH**_****3****_**CN]**^****+****^	
[TFE] (M)	0	2.0	0	2.0	0	2.5	0	1.5
*E*_cat/2_ (V)^a^	−2.85	−1.94	−2.64	−1.97	−2.74	−1.97	−2.42	
*i*_cat_/$i_{\text{p}}^{\text{b}}$	11.3	20.2	7.4	17.1	10.7	18.5	8.5	11.6
TOF (s^−1^)	25^c^^,^ ^d^	75 ± 3^c^^,^ ^e^	11^c^^,^ ^d^	75 ± 6^c^^,^ ^e^	22^c^^,^ ^d^	67^c^^,^ ^d^	14^c^^,^ ^d^	26^c^^,^ ^d^

a *All potentials reported vs. the ferricenium/ferrocene pseudo reference recorded at υ=0.1 V s^−1^*.

b *Calculated at υ = 0.1 V s^−1^*.

c *TOF calculated using n_p_ = 1 electron*.

d Steady-state conditions not achieved, calculated at υ=0.1 V s^−1^.

e *Average TOF_max_ determined over a range of scan rates at steady-state conditions (see Supporting Information)*.

### Voltammetry Under 1 Atm CO_2_ in the Presence of Trifluoroethanol

**Figure 7:**
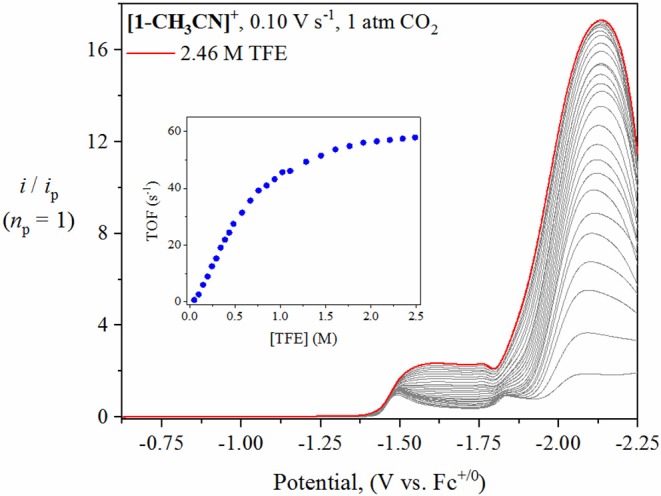
Linear sweep voltammetry of **[1-CH**_**3**_**CN]**^**+**^ recorded at υ=0.1 V s^−1^ under 1 atm CO_2_ with increasing TFE concentration (0–2.46 M). The current (y-axis) data are normalized with respect to the non-catalytic Faradaic response (*i*_p_). The inset plot of ‘TOF vs. TFE concentration’ demonstrates that zero-order conditions with respect to TFE concentration were achieved at 2.0 M TFE.

To further probe the catalytic activity of **[1-CH**_**3**_**CN]**^**+**^, **[2-CH**_**3**_**CN]**^**+**^, **[3-CH**_**3**_**CN]**^**+**^ and **[4-CH**_**3**_**CN]**^**+**^, the voltammetry conditions were altered by adding incremental amounts of a non-aqueous proton source. For the purpose of this study, 2,2,2-trifluoroethanol (TFE, p*K*_a(CH3CN)_ = 35.4(est.) Lam et al., 2015) was added as a Brønsted acid to observe and optimize proton-coupled catalytic CO_2_ reduction. The addition of at least 1.5 M TFE results in a plateau of catalytic current for **[1-CH**_**3**_**CN]**^**+**^, **[2-CH**_**3**_**CN]**^**+**^ and **[3-CH**_**3**_**CN]**^**+**^ when monitored at υ=0.1 V s^−1^ (Figure 7, Figures S16–S19). Notably, each of their catalytic waves exhibit a significant positive shift compared to experiments in the absence of excess Brønsted acid, with *E*_cat/2_ = −1.94 V for **[1-CH**_**3**_**CN]**^**+**^ and *E*_cat/2_ = −1.97 V for both **[2-CH**_**3**_**CN]**^**+**^ and **[3-CH**_**3**_**CN]**^**+**^. The predominant catalytic peak observed for **[1-CH**_**3**_**CN]**^**+**^, **[2-CH**_**3**_**CN]**^**+**^, and **[3-CH**_**3**_**CN]**^**+**^ is assigned, with the aid of computations, to the *reduction-first* pathway with the five-coordinate, two-electron reduced [*fac*-Mn^0^(N^∧^N^•−^)(CO)_3_]^−^ monoanion being the active catalyst.

Interestingly, a weak grow-in of catalytic current can also be observed at *E*_cat/2_ = −1.48 V for both **[1-CH**_**3**_**CN]**^**+**^ and **[2-CH**_**3**_**CN]**^**+**^ (a similar maximum is less discernable for **[3-CH**_**3**_**CN]**^**+**^). This lower overpotential weak catalytic current occurs directly from the one-electron reduced species and is thus attributed to catalytic activity of the Mn^0^-Mn^0^ dimer intermediate. Indirect support of this hypothesis is the fact that such weak catalytic current is not observed with the bqn pre-catalyst, **[4-CH**_**3**_**CN]**^**+**^ which we have already established, via IR-SEC studies, does not form a Mn^0^-Mn^0^ dimer intermediate. While appearance of this lower energy catalytic pathway is promising, the very weak current observed, and thus the minimal TOF (<1 s^−1^) prompted us to focus on the more efficient *reduction-first* pathway for these complexes. Furthermore, this mechanism has already been the focus of other independent studies (Bourrez et al., 2014; Neri et al., 2019).

**Figure 8:**
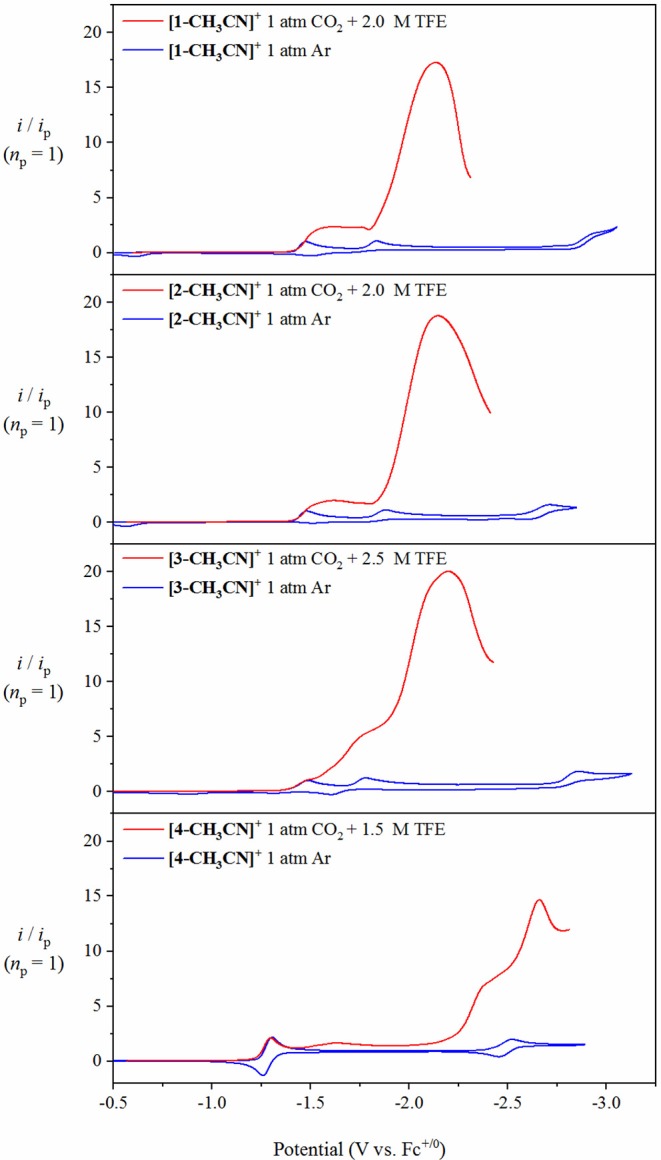
Overlay of cyclic voltammograms for **[1-CH**_**3**_**CN]**^**+**^, **[2-CH**_**3**_**CN]**^**+**^, **[3-CH**_**3**_**CN]**^**+**^ and **[4-CH**_**3**_**CN]**^**+**^ recorded under 1 atm of Ar (blue), with respective linear sweep voltammetry recorded under 1 atm CO_2_ in the presence of optimum TFE (red) at υ=0.1 V s^−1^ with 0.1 M [Bu_4_N][PF_6_] acetonitrile supporting electrolyte. The current (y-axis) data are normalized with respect to the non-catalytic Faradaic response (*i*_p_).

In contrast to the above mentioned studies in the absence of excess Brønsted acid, the presence of optimal TFE concentrations benefitted the pursuit of steady-state catalytic conditions, thus allowing for an estimation of a maximum turnover frequency (TOF_max_), at least in the case of **[1-CH**_**3**_**CN]**^**+**^ and **[2-CH**_**3**_**CN]**^**+**^. A collection of scan-rate dependent voltammetry data is provided in the Supporting Information for all pre-catalysts (Figures S20–S23) with kinetic analysis summarized in Table 3. Pre-catalysts **[1-CH**_**3**_**CN]**^**+**^ and **[2-CH**_**3**_**CN]**^**+**^ perform comparably with TOF_max_ values estimated at 75 s^−1^ under steady-state catalytic conditions. Pre-catalyst **[3-CH**_**3**_**CN]**^**+**^ still exhibits a scan-rate dependent current upon increasing scan rate >0.1 V s^−1^, thus preventing pure kinetic steady-state conditions, and hence TOF_max_, to be obtained. Thus, an estimated TOF value of 67 s^−1^ is reported for **[3-CH**_**3**_**CN]**^**+**^ recorded at a scan rate of υ=0.1 V s^−1^ (Figure S22). Results from the addition of TFE to **[4-CH**_**3**_**CN]**^**+**^ were rather complicated due to the grow-in of an additional pre-wave at approx. −2.42 V which prevented accurate determination of *E*_cat/2_ or TOF_max_ (Figure S23). At least from a qualitative perspective, it can be stated that TFE addition does give rise to a modest increase in the observed catalytic current maximum (Figure 8). Unfortunately, this phenomenon of multiple catalytic waves raises concerns about product selectivity and competitive side reactions, which are borne true following controlled potential electrolysis experiments, discussed below, which conclusively confirm hydrogen evolution as not just a competitive but a dominant process under these experimental conditions with **[4-CH**_**3**_**CN]**^**+**^. Mechanistic details of competitive hydrogen evolution vs. CO_2_ reduction are discussed in more detail below via computational analysis.

### Controlled Potential Electrolysis

Product selectivity of all four pre-catalysts in the absence and presence of TFE under 1 atmosphere of CO_2_ was investigated by controlled potential electrolysis (CPE) with *in-situ* gas chromatography analysis used for CO and H_2_ quantification over a time period of 4 h. The potential bias applied in each case corresponded to *E*_cat/2_ for specific experimental conditions as summarized in Table 3 for each catalyst. The tabulated Faradaic efficiency (FE) data in Table 4 represent the peak CO selectivity observed at a single time interval over the duration of the experiment, whereas the turnover number (TON) tabulated for both CO and H_2_ represents the total TON over the entire course of the experiment. Plots summarizing all electrolysis data are provided in Figures S28–S35. Ultimately, all pre-catalysts yielded CO product regardless of the conditions employed. Each of the pre-catalysts, **[1-CH**_**3**_**CN]**^**+**^, **[2-CH**_**3**_**CN]**^**+**^, and **[3-CH**_**3**_**CN]**^**+**^ exhibited a clear selectivity for CO production in the absence or presence of TFE with only **[4-CH**_**3**_**CN]**^**+**^ yielding significant H_2_ in the presence of 1.5 M TFE. Unfortunately, however, in most cases there is FE that is unaccounted for. This is especially true for pre-catalysts **[2-CH**_**3**_**CN]**^**+**^ and **[3-CH**_**3**_**CN]**^**+**^ which is likely due to rapid decomposition, consistent with their poor TONs for CO or H_2_ evolution. Pre-catalyst **[1-CH**_**3**_**CN]**^**+**^ performed significantly better, exhibiting percentage FE's of 62:2 CO:H_2_ in the absence of TFE, which increased to 84:2 in the presence of 2.0 M TFE. Efforts to quantify any formate (${\text{HCO}}_{2}^{-}$) production to account for the full FE of all catalysts were in vain. This is likely due to the low turnover numbers of these catalysts, which as a whole performed quite poorly under the experimental conditions employed; a recognized problem for the [*fac*-Mn(N^∧^N)(CO)_3_]^−^ class of electrocatalysts under homogeneous conditions (Grills et al., 2018). Indeed, two prior CPE studies of the [*fac*-MnBr(phen)(CO)_3_] pre-catalyst have each highlighted the poor performance of this system with the FE_CO_ ranging from 18 to 57% (Stanbury et al., 2017; Tignor et al., 2018). Interestingly, the bqn-derived pre-catalyst, **[4-CH**_**3**_**CN]**^**+**^ exhibited the greatest FE_CO_ of 98% in the absence of TFE, albeit with a very low TON of just 3. However, with such a low TON we cannot rule out catalyst decomposition as a contributing factor. As anticipated from its irregular voltammetry behavior, at least in comparison to **[1-CH**_**3**_**CN]**^**+**^, **[2-CH**_**3**_**CN]**^**+**^, and **[3-CH**_**3**_**CN]**^**+**^, upon the introduction of TFE, a dramatic shift in product selectivity was observed, with FE's of 14:69 CO:H_2_. As discussed below in the computational section, this shift in product selectivity is possibly a consequence of the reduced nucleophilicity of the two-electron reduced **[4]**^**−**^ active catalyst, in large part due to the lower lying π^*^ orbitals of the bqn ligand.

**Table 4:** Summary of controlled potential electrolysis data in the absence of TFE and at optimum TFE concentrations^a^.

	**[1-CH**_****3****_**CN]**^****+****^		**[2-CH**_****3****_**CN]**^****+****^		**[3-CH**_****3****_**CN]**^****+****^		**[4-CH**_****3****_**CN]**^****+****^	
[TFE] (M)	0	2.0	0	2.0	0	2.5	0	1.5
FE_CO_ (%)^b^	62	80	47	64	31	15	98	14
FE_H2_ (%)^b^	6	2	7	1	1	1	1	69
TON (CO:H_2_)^c^	17:9	18:12	6:2	3:2	4:1	4:2	3:1	2:10

a *Applied potential was equal to E_cat/2_ as summarized in Table 3 for each catalyst*.

b *Peak CO:H_2_ FE ratio observed over a 4 h duration*.

c *Totaled over a 4 h duration*.

### Computational Analysis

**Scheme 1:**
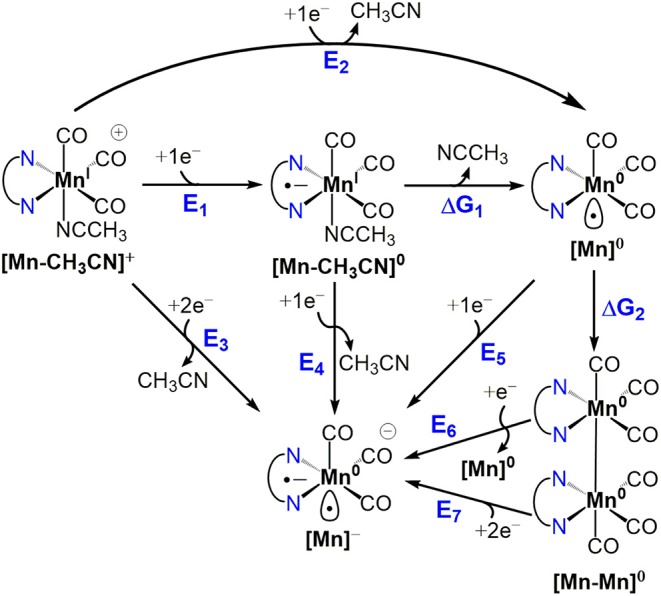
Catalyst activation and dimerization pathways for Mn^I^ polypyridyl complexes **[1-CH**_**3**_**CN]**^+^, **[2-CH**_**3**_**CN]**^+^ and **[4-CH**_**3**_**CN]**^+^.

**Scheme 2:**
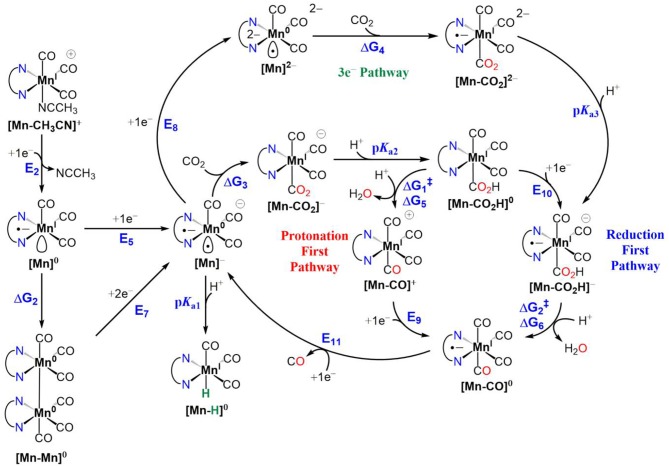
Electrocatalytic reduction of CO_2_ to CO for Mn^I^ polypyridyl complexes of **[1-CH**_**3**_**CN]**^+^, **[2-CH**_**3**_**CN]**^+^ and **[4-CH**_**3**_**CN]**^+^, illustrating active catalyst generation, protonation-first and reduction-first pathways vs. the three-electron reduction pathway. Also included alongside are competitive Mn^0^-Mn^0^ dimer formation and Mn^I^-H formation side reactions. For the C–OH bond cleavage steps, TFE is used as the Brønsted acid. The free energy changes (Δ*G*) and activation free energies (Δ*G*) are in units of kcal/mol and reduction potentials are in units of volts (V) vs. Fc^+/0^.

Density functional theory (DFT) calculations at the M06 level of theory (Zhao and Truhlar, 2008a,b, 2010) in conjunction with the SMD continuum solvation model (Marenich et al., 2009) for acetonitrile were performed to examine the catalyst activation and electrocatalytic CO_2_ reduction mechanisms of **[1-CH**_**3**_**CN]**^+^, **[2-CH**_**3**_**CN]**^+^, and **[4-CH**_**3**_**CN]**^+^. Mechanistic calculations on **[3-CH**_**3**_**CN]**^**+**^ were omitted for the sake of brevity due to its similarity in behavior to **[2-CH**_**3**_**CN]**^+^. The results are summarized in Schemes 1 and 2 along with tabulated energetics (Tables 5, 6). Further details on the electronic structures of selected reaction intermediates are provided in Supporting Information.

**Table 5:** Computed reduction potentials (V vs. Fc^+/0^) and free energy changes (Δ*G*, kcal/mol) of catalyst activation and dimerization pathways for Mn^I^ polypyridyl complexes **[1-CH**_**3**_**CN]**^+^, **[2-CH**_**3**_**CN]**^+^, and **[4-CH**_**3**_**CN]**^+^.

	**[1-CH_**3**_CN]^**+**^**	**[2-CH_**3**_CN]^**+**^**	**[4-CH_**3**_CN]^**+**^**
***E***_**1**_	−1.74 V	−1.77 V	−1.23 V
**Δ*****G***_**1**_	−6.6 kcal/mol	−6.6 kcal/mol	−0.4 kcal/mol
***E***_**2**_	−1.45 V	−1.49 V	−1.21 V
***E***_**3**_	−1.62 V	−1.66 V	−1.36 V
***E***_**4**_	−1.50 V	−1.54 V	−1.48 V
***E***_**5**_	−1.79 V	−1.83 V	−1.50 V
**Δ*****G***_**2**_	−11.5 kcal/mol	−13.4 kcal/mol	−1.2 kcal/mol
***E***_**6**_	−2.29 V	−2.41 V	−1.55 V
***E***_**7**_	−2.04 V	−2.12 V	−1.52 V

**Table 6:** Computed reduction potentials (V vs. Fc^+/0^), free energy changes (Δ*G*, kcal/mol) and activation free energies (Δ*G*^‡^, kcal/mol), and p*K*_a_'s relevant for electrocatalytic reduction of CO_2_ to CO for Mn^I^ polypyridyl complexes **[1-CH**_**3**_**CN]**^+^, **[2-CH**_**3**_**CN]**^+^, and **[4-CH**_**3**_**CN]**^+^, shown in Scheme 2, comparing the *protonation-first* and *reduction-first* pathways vs. the *three-electron* reduction pathway.

	**[1-CH_**3**_CN]^**+**^**	**[2-CH_**3**_CN]^**+**^**	**[4-CH_**3**_CN]^**+**^**
***E***_**2**_	−1.45 V	−1.49 V	−1.21 V
**Δ*****G***_**2**_	−11.5 kcal/mol	−13.4 kcal/mol	−1.2 kcal/mol
***E***_**5**_	−1.79 V	−1.83 V	−1.50 V
***E***_**7**_	−2.04 V	−2.12 V	−1.52 V
**p*****K***_****a1****_	24.8	25.2	17.3
**Δ*****G***_**3**_	8.6 kcal/mol	8.7 kcal/mol	–
**p*****K***_****a2****_	23.7	24.3	–
***E***_**8**_	−2.71 V	−2.53 V	−2.25 V
**Δ*****G***_**4**_	1.5 kcal/mol	5.2 kcal/mol	10.8 kcal/mol
**p*****K***_****a3****_	30.0	29.6	29.4
**Δ**$G_{1}^{‡}$	23.8 kcal/mol	22.6 kcal/mol	28.8 kcal/mol
**Δ*****G***_**5**_	17.6 kcal/mol	16.8 kcal/mol	21.1 kcal/mol
***E***_**9**_	−1.61 V	−1.70 V	−1.12 V
***E***_**10**_	−2.03 V	−2.07 V	−1.57 V
**Δ**$G_{2}^{‡}$	18.6 kcal/mol	–	20.9 kcal/mol
**Δ*****G***_**6**_	8.0 kcal/mol	8.5 kcal/mol	10.6 kcal/mol
***E***_**11**_	−1.91 V	−1.88 V	−1.82 V

#### Catalyst Activation and Dimer Formation

The activation of each catalyst starts with one-electron reduction of the solvent-coordinated **[Mn-CH**_**3**_**CN]**^**+**^ species. For all considered pathways in the present study, **[1-CH**_**3**_**CN]**^+^ and **[2-CH**_**3**_**CN]**^+^ exhibit similar energetics (Scheme 1), so henceforth we will focus on the activation of **[1-CH**_**3**_**CN]**^+^ to highlight the distinct behavior of **[4-CH**_**3**_**CN]**^+^. The one-electron reduction of **[Mn-CH**_**3**_**CN]**^**+**^ (*E*_1_) results in the formation of a ligand-based radical anion for all of the complexes investigated (Figure S36). Typically observed as an *EC* mechanism in voltammetry (*E*_1_Δ*G*_1_ in Scheme 1), subsequent dissociation of the acetonitrile ligand (Δ*G*_1_) leads to localization of the unpaired spin on the Mn center, generating a formally Mn^0^ species (**[Mn]**^**0**^) (Figure S36). The extensive π-conjugation of **[4-CH**_**3**_**CN]**^+^ introduces an ~+0.5 V anodic shift (*E*_1_ = −1.23 V) compared to that of **[1-CH**_**3**_**CN]**^+^ (*E*_1_ = −1.74 V), in line with the assumption of an initial ligand-based reduction. The computed energetics indicate that acetonitrile dissociation is very favorable upon reduction of **[1-CH**_**3**_**CN]**^+^ (Δ*G*_1_ = −6.6 kcal/mol) to generate pentacoordinate **[1]**^**0**^, further reduction of which (*E*_5_ = −1.79 V) will form **[1]**^−^. In contrast, theory predicts that **[4-CH**_**3**_**CN]**^**0**^ and **[4]**^**0**^ will coexist based on acetonitrile dissociation being nearly isoergic (Δ*G*_1_ = −0.4 kcal/mol). This distinct behavior is responsible for the experimentally observed concerted two-electron reduction of **[4-CH**_**3**_**CN]**^**+**^ to **[4]**^−^ (*E*_3_ = −1.36 V), which requires a lower potential than the sequential one-electron reduction pathway, i.e., **[4-CH**_**3**_**CN]**^**0**^ to **[4]**^−^ conversion (*E*_4_ = −1.48 V) in contrast to **[1-CH**_**3**_**CN]**^+^ (*E*_3_ = −1.62 V and *E*_4_ = −1.50 V).

Another intriguing difference is in the energetics of dimerization of **[Mn]**^**0**^ to **[Mn-Mn]**^**0**^, which is quite favorable for **[1]**^**0**^ (Δ*G*_2_ = −11.5 kcal/mol), in contrast to a nearly isoergic driving force in the case of **[4]**^**0**^ (Δ*G*_2_ = −1.2 kcal/mol). Closer inspection of the dimer geometries indicates that staggered conformations are more favorable and Mn–Mn distances are predicted as 2.96 Å and 3.16 Å for **[1–1]**^**0**^ and **[4–4]**^**0**^, respectively (Figure S37). This difference is partly attributed to ligand-induced steric effects that lead the Mn center to be out-of-plane with respect to the ligand in the case of **[4]**^**0**^ (for **[4]**^**0**^, C_bridge_-N-Mn-N is 23.2°, compared to 1.1° for **[1]**^**0**^). It should be noted that dimerization of **[4]**^**0**^ is even further suppressed due to the predicted equilibrium between hexacoordinate **[4-CH**_**3**_**CN]**^**0**^ and pentacoordinate **[4]**^**0**^, even if the latter forms during electrocatalysis. As a result of the computational analysis described above, **[1-CH**_**3**_**CN]**^+^ and **[2-CH**_**3**_**CN]**^+^ are predicted to form **[1]**^**0**^ and **[2]**^**0**^, respectively, via an *EC* process followed by fast dimerization to form **[1–1]**^**0**^ and **[2–2]**^**0**^, respectively, which can then be reduced via sequential one-electron processes, or a concerted two-electron reduction process, to generate the catalytically active **[1]**^−^ and **[2]**^−^ species. We should note that two-electron reduction of the **[Mn-Mn]**^**0**^ dimer practically proceeds via two sequential one-electron reduction steps as the dimer is expected to decompose into **[Mn]**^**0**^ and **[Mn]**^**−**^ upon the first reduction, and the computed reduction potential of **[Mn]**^**0**^ is more anodic than that of **[Mn-Mn]**^**0**^ (e.g., *E*_5_ **=** −1.79 V for **[1]**^**0**^ vs. *E*_6_ **=** −2.29 V for **[1–1]**^**0**^).

#### CO_2_ Binding and CO Evolution

Next, we turn our attention to CO_2_ binding to the two-electron reduced **[Mn]**^−^ catalyst and subsequent steps of electrocatalytic CO_2_ reduction (Scheme 2). Similar to earlier reports (Riplinger et al., 2014), CO_2_ binding to **[1]**^−^ is computed to be uphill (Δ*G*_3_ = 8.6 kcal/mol) and is driven by proton transfer from a Brønsted acid to generate **[1-CO**_**2**_**H]**^**0**^ (p*K*_a1_ = 24.8). Interestingly, we could not locate an optimized structure of CO_2_-bound **[4-CO**_**2**_**]**^−^, indicating that since the potential to generate **[4]**^−^ is nearly +0.5 V more positive compared to that of **[1]**^−^, the pentacoordinate **[4]**^−^ does not possess enough reducing power to activate CO_2_. On the other hand, further reduction to **[4]**^**2**^^−^ (Figure S38), results in increased reactivity toward CO_2_ to generate **[4-CO**_**2**_**]**^**2**^^−^ (Δ*G*_4_ = 10.8 kcal/mol), and subsequent protonation yields **[4-CO**_**2**_**H]**^−^ (p*K*_a3_ = 29.4) (Scheme 2). It should be noted that the spin density (Figure S38) and total electron density difference (Figure S39) plots indicate that the third reduction is predominantly ligand centered in **[4]**^**2**^^−^ and best characterized as [Mn^0^-bqn^2−^]^2−^, although the additional negative charge is shared between the metal center and the ligand in the case of **[1]**^**2**^^−^ and **[2]**^**2**^^−^ such that the electronic structure exhibits a resonance between [Mn^−^-(N^∧^N)^•−^]^2−^ and [Mn^0^-(N^∧^N)^2−^]^2−^. **[Mn-CO**_**2**_**H]**^−^ is predicted to be the common intermediate for both **[1-CH**_**3**_**CN]**^+^ and **[4-CH**_**3**_**CN]**^+^ electrocatalysts, as C–OH bond cleavage is expected to proceed predominantly via the *reduction-first* pathway (Scheme 2). Scission of the C–OH bond in **[Mn-CO**_**2**_**H]**^−^ assisted by TFE leads to **[Mn-CO]**^**0**^. CO evolution may occur spontaneously from **[Mn-CO]**^**0**^ prior to one-electron reduction (Grice et al., 2013) and generation of **[Mn]**^−^. However, this mechanism is yet to be experimentally verified for this class of Mn catalyst. In contrast to **[1-CH**_**3**_**CN]**^+^, **[4-CH**_**3**_**CN]**^+^ produces H_2_ as the dominant product in the presence of TFE as a Brønsted acid, which is attributed to less favorable interaction of **[4]**^−^ with CO_2_ compared to **[1]**^−^. However, we should also note that the p*K*_a_'s of **[1]**^−^ (p*K*_a_ = 24.8) and **[1]**^**2**^^−^ (p*K*_a_ = 36.0) are significantly higher than their counterparts, **[4]**^−^ (p*K*_a_ = 17.3) and **[4]**^**2**^^−^ (p*K*_a_ = 29.0), indicating that hydride formation is not as favorable in the latter either. In the absence of a Brønsted acid as a proton source, all the catalysts are predicted to form the three-electron reduced **[Mn]**^**2**^^−^ active catalyst before binding CO_2_ and subsequently producing CO and ${\text{CO}}_{3}^{2-}$ via interaction of a second CO_2_ molecule with **[Mn-CO**_**2**_**]**^**2**^^−^.

## Conclusions

Through a systematic variation of the polypyridyl ligand from bpy to phen to dmphen and finally bqn, both steric- and electronic-based ligand influences on the activity of a [*fac-*Mn(N^∧^N)(CO)_3_(CH_3_CN)]^+^ class of CO_2_ reduction pre-catalysts has been established, providing critical insight into the manipulation of CO_2_ binding affinities and resulting product selectivity for these catalysts. Through a combination of IR-SEC and computational studies, the combined electronic and steric influences of the π-extended bqn ligand have been probed in **[4-CH**_**3**_**CN]**^**+**^, where formation of the **[4–4]**^**0**^ dimer is hindered and a concerted two-electron *ECE* mechanism for the generation of **[4]**^**−**^ is favored. Computations have revealed how the lower lying π^*^-orbitals of the bqn ligand have rendered this pentacoordinate **[4]**^−^ intermediate inactive with respect to CO_2_, even in the presence of a Brønsted acid. In contrast to the bpy, phen, and dmphen derived catalysts, this has resulted in a shift in product selectivity for the **[4-CH**_**3**_**CN]**^**+**^ pre-catalyst to favor H_2_ evolution in the presence of excess TFE. However, electrochemical and computational investigations have established successful CO_2_ activation following *in-situ* generation of a three-electron reduced [*fac-*Mn^(−1)^(N^∧^N^•−^)(CO)_3_]^2−^ active catalyst, a first for any [*fac-*Mn(N^∧^N)(CO)_3_(CH_3_CN)]^+^ pre-catalyst. Although at the cost of additional overpotential, this *three-electron* pathway results in increased reactivity toward CO_2_ to generate the previously established [*fac-*Mn^(I)^(CO_2_H)(N^∧^N^•−^)(CO)_3_]^−^ intermediate, which subsequently propagates the catalytic cycle via the standard *reduction-first* pathway involving rate-determining, proton-coupled C–OH bond cleavage.

## Materials and Methods

Acetonitrile (ACS reagent grade, 99.5%), bromopentacarbonylmanganese(I) (98%), potassium carbonate (>99%), silver trifluoromethanesulfonate (>99%), tetrahydrofuran (anhydrous, 99.9%) and 2,2,2-trifluoroethanol (>99%) were purchased from Sigma Aldrich and used as received. Dichloromethane (ACS reagent grade, >99.9%) and diethyl ether were purchased from Pharmco-Aaper (ACS reagent grade, >99.9%) and used as received. The water content in ACS reagent grade acetonitrile was confirmed by Karl-Fisher titration to be 0.17 M (0.3%). Tetrabutylammonium hexafluorophosphate (99%, Sigma Aldrich) was recrystallized thrice from ethanol and dried under vacuum prior to electrolyte preparation. Steady-state FTIR spectra were recorded on a Thermo Nicolet 670 FTIR spectrophotometer using a liquid cell with CaF_2_ windows in spectrophotometric grade acetonitrile (99.5%, Sigma Aldrich) solvent. NMR spectra were recorded on an Agilent spectrometer operated at 399.80 MHz for ^1^H nuclei. CD_3_CN was used as received from Sigma Aldrich and its residual ^1^H solvent signal used as an internal reference for reporting the chemical shift (δ=1.96 ppm). Voltammetry and bulk electrolysis were carried out on a CH Instruments 620E potentiostat. A custom three-electrode cell was used for both voltammetry and bulk electrolysis experiments allowing airtight introduction of working, counter and reference electrodes as well as septa for gas purging. For cyclic voltammetry, glassy carbon (3 mm diameter) and Pt wire were used as working and counter electrodes, respectively, with 0.1 M [Bu_4_N][PF_6_] in ACS reagent grade acetonitrile as the supporting electrolyte. A non-aqueous reference electrode was used to minimize ohmic potential drop at the solvent interface. This consisted of a Ag wire in 0.10 M [Bu_4_N][PF_6_] acetonitrile supporting electrolyte isolated by a vycor frit and was calibrated *in-situ* using the ferricenium/ferrocene redox couple as an internal reference. Redox potentials (*E*) were determined from cyclic voltammetry as (*E*_pa_ + *E*_pc_)/2, where *E*_pa_ and *E*_pc_ are the anodic and cathodic peak potentials, respectively. Where *E* could not be calculated due to irreversible behavior, *E*_pc_ or *E*_pa_ are reported accordingly. For CO_2_ concentration dependent studies, gas cylinders were ordered from Airgas containing pre-mixed ratios of Ar:CO_2_ (100:0, 80:20, 60:40, 50:50, 40:60, 20:80, 0:100). For controlled potential bulk electrolysis experiments a vitreous carbon (Duocell) working electrode soldered to a copper wire was used. A Pt gauze counter electrode was used, isolated from the main compartment by a fine porosity vycor tube+frit to minimize mass transfer resistance. Gas chromatography data were recorded on a custom Shimadzu GC-2014 instrument where a Ni “methanizer” catalyst was used to convert CO to CH_4_ prior to quantification of CH_4_ by the thermal conductivity detector. H_2_ was simultaneously monitored by a flame ionization detector during the same injection. The GC was pre-calibrated for CO and H_2_ quantification by mimicking bulk electrolysis conditions (i.e., 5 mL supporting electrolyte in the same cell, with electrodes, under 1 atm CO_2_). Standard curves for H_2_ and CO were generated using this cell where known volumes of the analyte gas (H_2_ or CO) were injected and the solution stirred for 30 min to allow equilibration of the analyte between the electrolyte and headspace prior to GC injection.

### General Synthesis of [*fac*-Mn(N^∧^N)(CO)_3_(OTf)] Complexes 1-4

Bromopentacarbonyl manganese(I) (100 mg, 0.36 mmol) and silver trifluoromethanesulfonate (93.5 mg, 0.36 mmol) were charged to a 50-mL round bottomed flask with approximately 25 mL of degassed dichloromethane under 1 atmosphere of argon. The solution was allowed to mix in the dark for 1 h at room temperature after which the AgBr precipitate was removed by filtration through celite. The dichloromethane solvent was subsequently removed by a rotary evaporator. After confirming quantitative transformation to the [*fac-*Mn(OTf)(CO)_5_] intermediate by FTIR spectroscopy, the solid was transferred to a 10-mL microwave vial with 3 mL of tetrahydrofuran. To this solution was added a slight deficit of the appropriate ligand (0.33 mol). The vial was then sealed and reacted in a CEM Discovery microwave reactor at 70°C for 10 min. Each reaction afforded a bright yellow-orange solution. The tetrahydrofuran was reduced in volume to roughly 0.5 mL on a rotary evaporator at which point the pure product was immediately precipitated by addition of excess diethyl ether. The solid was isolated via vacuum filtration, rinsed with diethyl ether and dried under vacuum. No further purification was necessary.

**[*fac*-Mn(OTf)(bpy)(CO)**_**3**_**] (1)** FTIR (CH_3_CN) ν(CO): 2,050, 1,958 cm^−1^. ^1^H-NMR (CD_3_CN) δ: 7.70–7.73 (2H, m), 8.21–8.25 (2H, m), 8.40 (2H, d, *J*=8.0 Hz), 9.14 (2H, d, *J*=5.6 Hz) ppm. Anal. Calcd. for C_14_H_8_F_3_MnN_2_O_6_S: C, 37.85; H, 1.82; N, 6.31. Found: C, 38.27; H, 1.99; N, 6.02.

**[*fac*-Mn(OTf)(phen)(CO)**_**3**_**] (2)** FTIR (CH_3_CN) ν(CO): 2,050, 1,958 cm^−1^. ^1^H-NMR (CD_3_CN) δ: 8.04 (2H, dd, *J*_1_ = 4.0, *J*_2_ = 5.2 Hz), 8.20 (2H, s), 8.78 (2H, dd, *J*_1_ = 1.2, *J*_2_ = 8.0 Hz), 9.49 (2H, dd, *J*_1_ = 1.2, *J*_2_ = 4.8 Hz) ppm. Anal. Calcd. for C_16_H_8_F_3_MnN_2_O_6_S: C, 41.04; H, 1.72; N, 5.98. Found: C, 41.36; H, 1.88; N, 5.42.

**[*fac*-Mn(OTf)(dmphen)(CO)**_**3**_**] (3)** FTIR (CH_3_CN) ν(CO): 2,046, 1,959, 1,944(sh) cm^−1^. ^1^H-NMR (CD_3_CN) δ: 3.30 (6H, s), 7.90 (2H, d, *J*=8.0 Hz), 8.04 (2H, s), 8.57 (2H, d, *J*=8.0 Hz). Anal. Calcd. for C_18_H_12_F_3_MnN_2_O_6_S: C, 43.56; H, 2.44; N, 5.64. Found: C, 44.05; H, 2.69; N, 5.18.

**[*fac*-Mn(OTf)(bqn)(CO)**_**3**_**] (4)** FTIR (CH_3_CN) ν(CO): 2,047, 1,959 cm^−1^. ^1^H-NMR (CD_3_CN) δ: 7.90 (2H, dd, *J*_1_ = *J*_2_ = 8.0 Hz), 8.12 (2H, dd, *J*_1_ = *J*_2_ = 8.0 Hz), 8.21 (2H, d, *J*=8.0 Hz), 8.61 (2H, d, *J*=8.0 Hz), 8.83 (2H, d, *J*=8.0 Hz), 8.88 (2H, d, *J*=8.0 Hz) ppm. Anal. Calcd. for C_22_H_12_F_3_MnN_2_O_6_S: C, 48.54; H, 2.22; N, 5.15. Found: C, 49.10; H, 2.60; N, 4.91.

### Computational Methods

#### Density Functional Theory

All geometries were fully optimized at the M06 level of density functional theory (Zhao and Truhlar, 2008a,b, 2010) with the SMD continuum solvation model (Marenich et al., 2009) for acetonitrile as solvent using the Stuttgart [8s7p6d2f | 6s5p3d1f] ECP10MDF contracted pseudopotential basis set (Dolg et al., 1987) on Mn and the 6-31G(d) basis set on all other atoms (Hehre et al., 1986). Non-analytical integrals were evaluated using the integral=grid=ultrafine option as implemented in the Gaussian 16 software package (Frisch et al., 2016). The nature of all stationary points was verified by analytic computation of vibrational frequencies, which were also used for the computation of zero-point vibrational energies, molecular partition functions, and for determining the reactants and products associated with each transition-state structure (by following the normal modes associated with imaginary frequencies). Partition functions were used in the computation of 298 K thermal contributions to the free energy employing the usual ideal-gas, rigid-rotator, harmonic oscillator approximation (Cramer, 2004). Free-energy contributions were added to single-point, SMD-solvated M06 electronic energies computed at the optimized geometries obtained with the initial basis with the SDD basis set on Mn and the larger 6-311+G(2df,p) basis set on all other atoms to arrive at final, composite free energies.

#### Solvation and Standard Reduction Potentials

As mentioned above, solvation effects for acetonitrile were accounted for by using the SMD continuum solvation model. A 1 M standard state was used for all species in solution (except for acetonitrile as solvent for which the standard state was assigned as 19.14 M). Thus, the free energy in solution is computed as the 1 atm gas-phase free energy, plus an adjustment for the 1 atm to 1 M standard-state concentration change of *RT* ln (24.5), or 1.9 kcal/mol, plus the 1 M to 1 M transfer (solvation) free energy computed from the SMD model. Standard reduction potentials were calculated for various possible redox couples to assess the energetic accessibility of different intermediates at various oxidation states. For a redox reaction of the form

(4) $$\begin{array}{r} O_{\text{(soln)}}\;+\;ne_{\text{(g)}}^{-}\to \;R_{\text{(soln)}} \end{array}$$

where *O* and *R* denote the oxidized and reduced states of the redox couple, respectively, and *n* is the number of electrons involved in redox reaction, the reduction potential $E_{O|R}^{\text{o}}$ relative to SCE was computed as

(5) $$\begin{array}{r} E_{O|R}^{\text{o}}=-\frac{\Delta G_{O|R}^{\text{o}}}{nF}-\Delta E_{ref}^{\text{o}} \end{array}$$

where $\Delta G_{O|R}^{\text{o}}$ is the free energy change associated with Equation 1 (using Boltzmann statistics for the electron) and $\Delta E_{ref}^{\text{o}}$ is taken as 0.141 V (Keith et al., 2013), which is required for the conversion of calculated $E_{O|R}^{\text{o}}$ vs. normal hydrogen electrode (NHE) in aqueous solution (*E*_NHE_ = −4.281 V) (Kelly et al., 2006) to $E_{O|R}^{\text{o}}$ vs. the saturated calomel electrode (SCE) in acetonitrile (*E*_SCE_ = −4.422 V) (Isse and Gennaro, 2010). We obtained reduction potentials referenced to the ferricenium/ferrocene couple by using a shift of −0.384 V from $E_{O|R}^{\text{o}}$ vs. SCE.

## Data Availability Statement

All datasets generated for this study are included in the manuscript/Supplementary Files.

## Author Contributions

MM, VB, and JR contributed to the synthesis, spectroscopic, and electrochemical characterization of all complexes studied. KN and DG contributed IR-SEC studies. ME contributed all computational studies. MM, ME, DG, and JR contributed equally to manuscript preparation.

### Conflict of Interest

The authors declare that the research was conducted in the absence of any commercial or financial relationships that could be construed as a potential conflict of interest.

## Supplementary Material

Scan rate dependent voltammetry studies under inert and catalytic conditions; experimental and computational IR-SEC spectra; catalyst, acid and CO_2_ concentration dependence; controlled potential electrolysis plots; computational spin-density plots, total electron density difference plots, and cartesian coordinates. These materials are provided in the supporting information document.
