# Supplementary material for: An Investigation of Electrocatalytic CO2 Reduction Using a Manganese Tricarbonyl Biquinoline Complex
Source: Front Chem. 2019 Sep 24;7:628. doi: 10.3389/fchem.2019.00628 (PMC6771302; doi:10.3389/fchem.2019.00628)
Supplement: Supplementary file 1 [file Data_Sheet_1.PDF]

An investigation of electrocatalytic CO_2 reduction using a manganese tricarbonyl biquinoline complex

Supporting Information

Meaghan McKinnon[†], *Veronika Belkina*[†], *Ken T. Ngo*[†], *Mehmed Z. Ertem*[‡]*, *David C. Grills*[‡]*, *Jonathan Rochford* [†]*

[†]Department of Chemistry, University of Massachusetts Boston, 100 Morrissey Boulevard, Boston, MA 02125, USA. [‡]Chemistry Division, Brookhaven National Laboratory, Upton, NY 11793-5000, USA.

Jonathan.Rochford@umb.edu

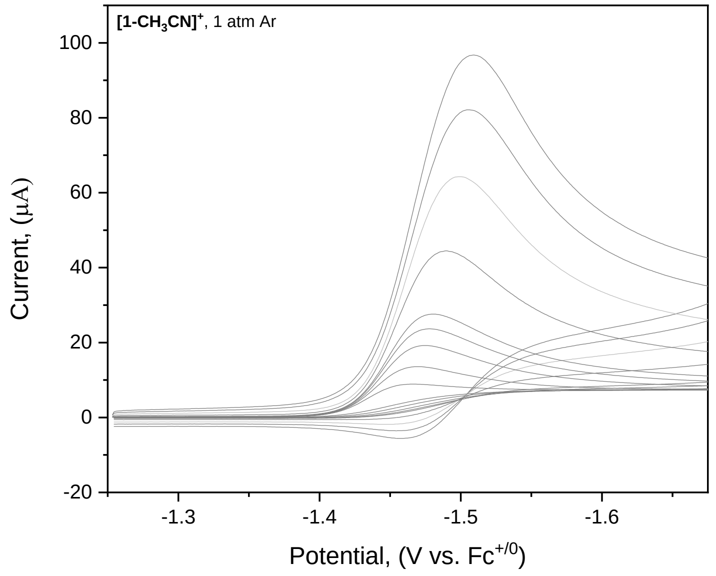

Figure S1. Scan rate dependent cyclic voltammetry data for the first irreversible, one electron reduction of **[1-CH$_3$CN]$^+$** recorded under 1 atm of argon in 0.1 M [Bu$_4$N][PF$_6$] acetonitrile supporting electrolyte. CVs were recorded at scan rates of 0.01, 0.025, 0.05, 0.075, 0.1, 0.25, 0.5, 0.75, and 1.0 V s^{-1}.

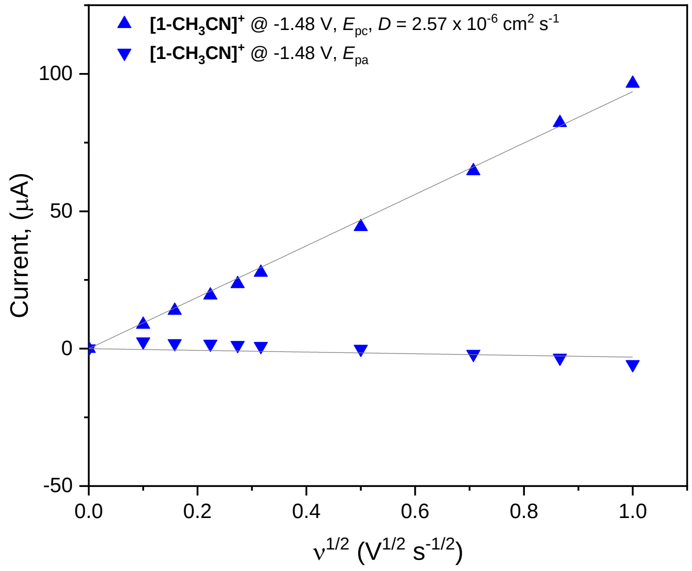

Figure S2. Randles-Sevcik plot for the first one-electron reduction of **[1-CH$_3$CN]$^+$** recorded at 1 mM catalyst concentration in 0.1 M [Bu$_4$N][PF$_6$] acetonitrile supporting electrolyte under 1 atm of argon. Scan rates of 0.01, 0.025, 0.05, 0.075, 0.1, 0.25, 0.5, 0.75, and 1.0 V s^{-1} were used.

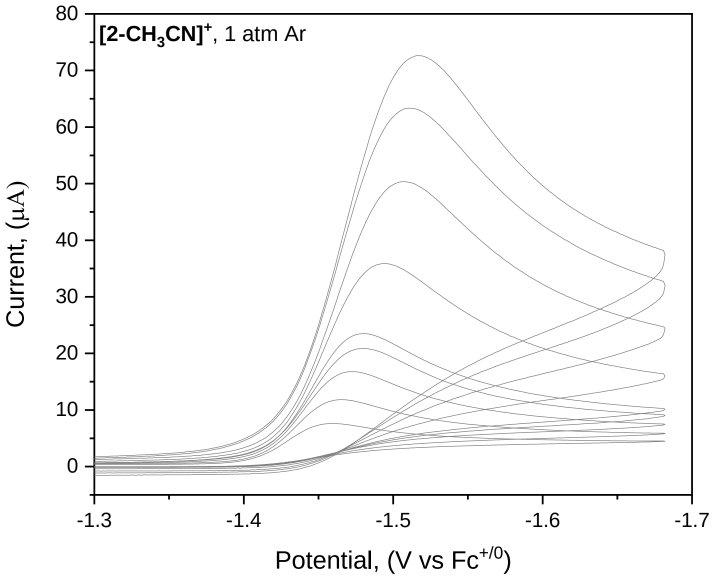

Figure S3. Scan rate dependent cyclic voltammetry data for the first irreversible, one electron reduction of **[2-CH_3CN]$^+$** recorded under 1 atm of argon in 0.1 M [Bu_4N][PF_6] acetonitrile supporting electrolyte. CVs were recorded at scan rates of 0.01, 0.025, 0.05, 0.075, 0.1, 0.25, 0.5, 0.75, and 1.0 V s^{-1}.

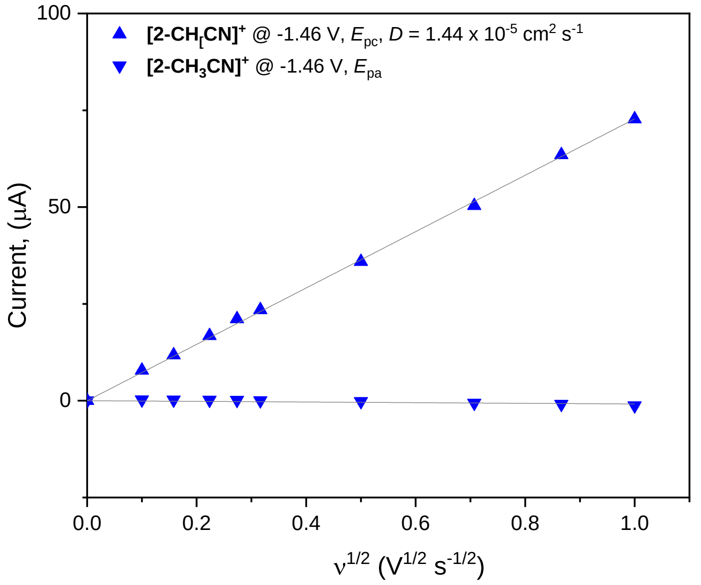

Figure S4. Randles-Sevcik plot for the first one-electron reduction of **[2-CH_3CN]$^+$** recorded at 1 mM catalyst concentration in 0.1 M [Bu_4N][PF_6] acetonitrile supporting electrolyte under 1 atm of argon. Scan rates of 0.01, 0.025, 0.05, 0.075, 0.1, 0.25, 0.5, 0.75, and 1.0 V s^{-1} were used.

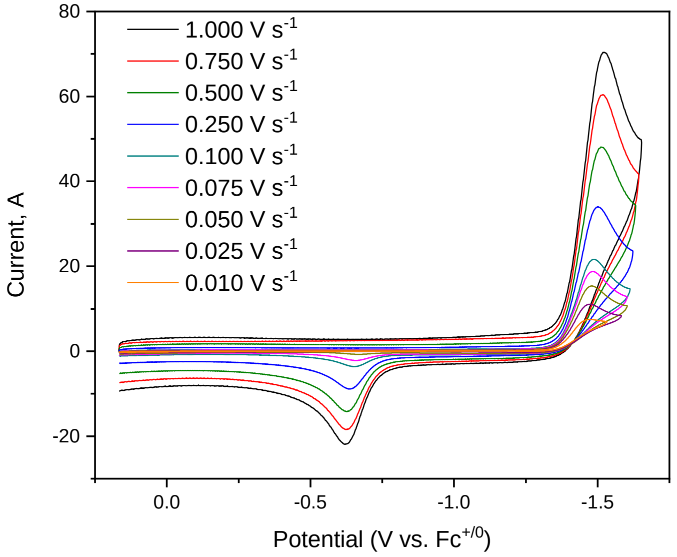

Figure S5. Scan rate dependent cyclic voltammetry data for the first irreversible, one electron reduction of **[3-CH₃CN]**$^+$, and subsequent **[3–3]**^{0} dimer oxidation, recorded under 1 atm of argon in 0.1 M $[Bu_4N][PF_6]$ acetonitrile supporting electrolyte. CVs were recorded at scan rates of 0.01, 0.025, 0.05, 0.075, 0.1, 0.25, 0.5, 0.75, and 1.0 V s^{-1}.

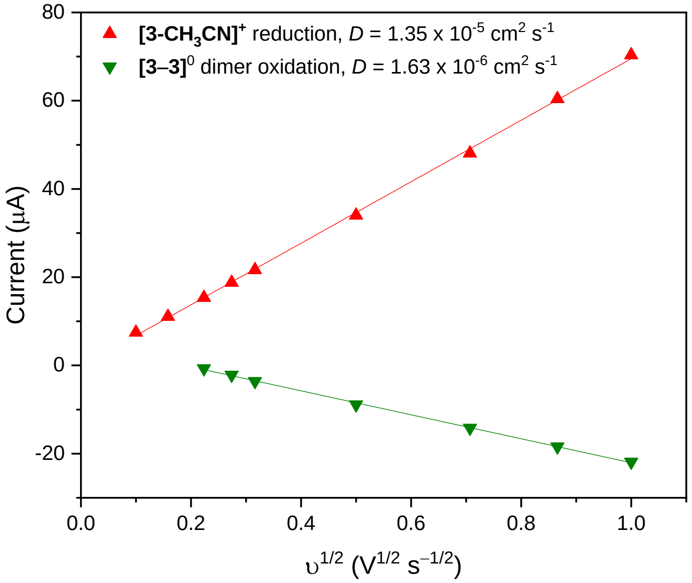

Figure S6. Randles-Sevcik plot for the first one-electron reduction of **[3-CH₃CN]**$^+$, and subsequent **[3–3]**^{0} dimer oxidation, recorded at 1 mM catalyst concentration in 0.1 M $[Bu_4N][PF_6]$ acetonitrile supporting electrolyte under 1 atm of argon. Scan rates of 0.01, 0.025, 0.05, 0.075, 0.1, 0.25, 0.5, 0.75, and 1.0 V s^{-1} were used.

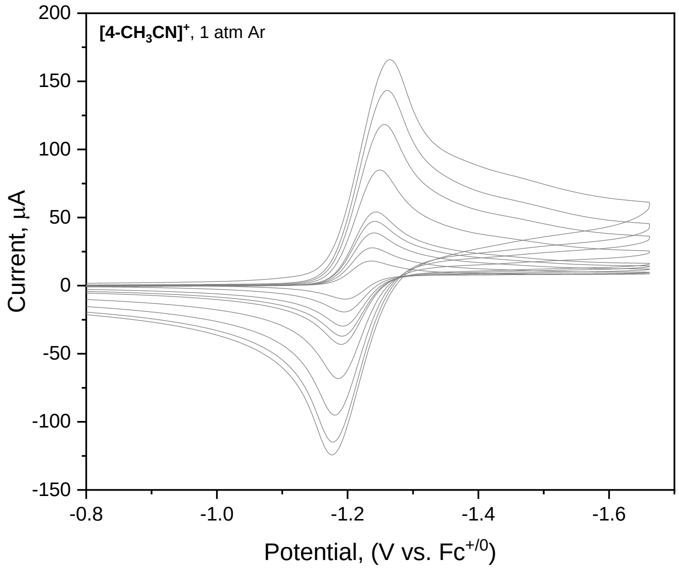

Figure S7. Scan rate dependent cyclic voltammetry data for the concerted two-electron reduction of **[4-CH_3CN]$^+$** recorded under 1 atm of argon in 0.1 M [Bu_4N][PF_6] acetonitrile supporting electrolyte. CVs were recorded at scan rates of 0.01, 0.025, 0.05, 0.075, 0.1, 0.25, 0.5, 0.75, and 1.0 V s^{-1}.

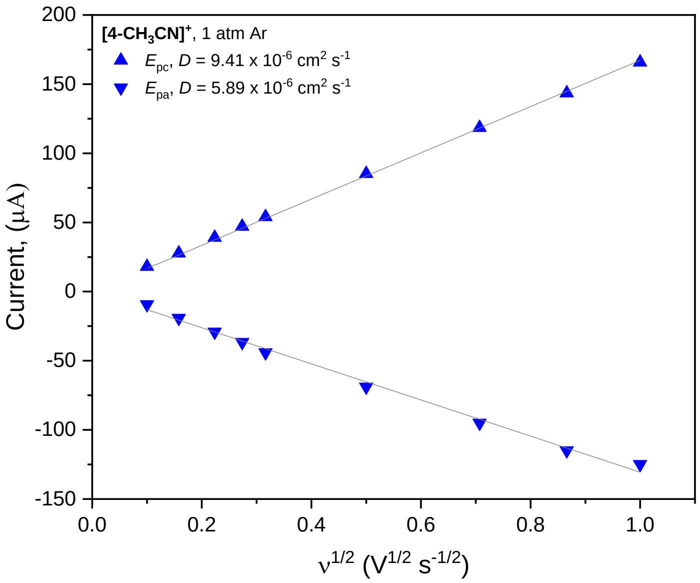

Figure S8. Randles-Sevcik plot for the concerted two-electron reduction of **[4-CH_3CN]$^+$** recorded at 1 mM catalyst concentration in 0.1 M [Bu_4N][PF_6] acetonitrile supporting electrolyte. Scan rates of 0.01, 0.025, 0.05, 0.075, 0.1, 0.25, 0.5, 0.75, and 1.0 V s^{-1} were used to measure and compare i_{pc} and i_{pa} to demonstrate redox reversibility.

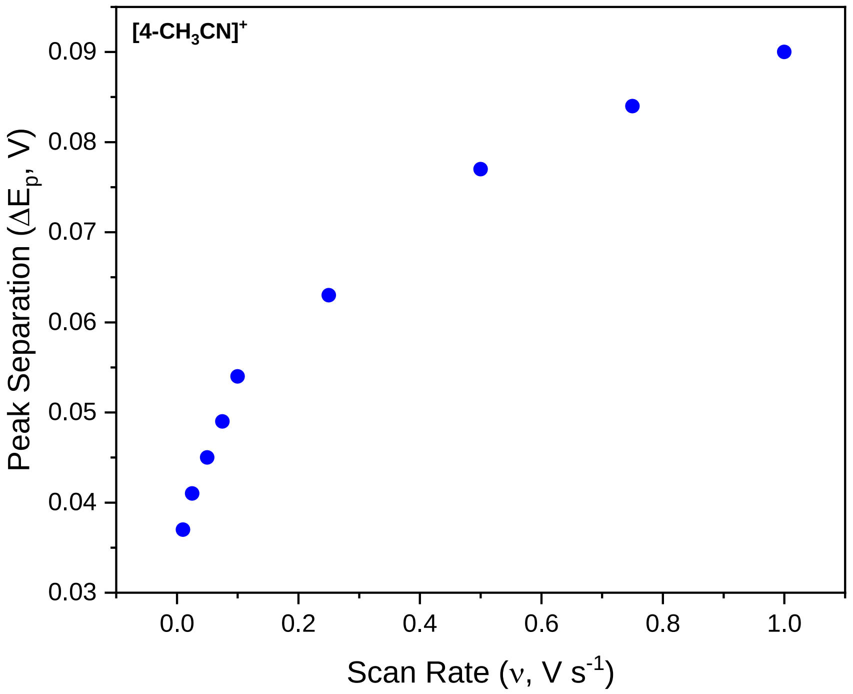

Figure S9. Peak separation dependence on scan rate for the concerted two-electron reduction of **[4-CH_3CN]$^+$** recorded at 1 mM catalyst concentration in 0.1 M [Bu_4N][PF_6] acetonitrile supporting electrolyte. Scan rates of 0.01, 0.025, 0.05, 0.075, 0.1, 0.25, 0.5, 0.75, and 1.0 V s^{-1} were used.

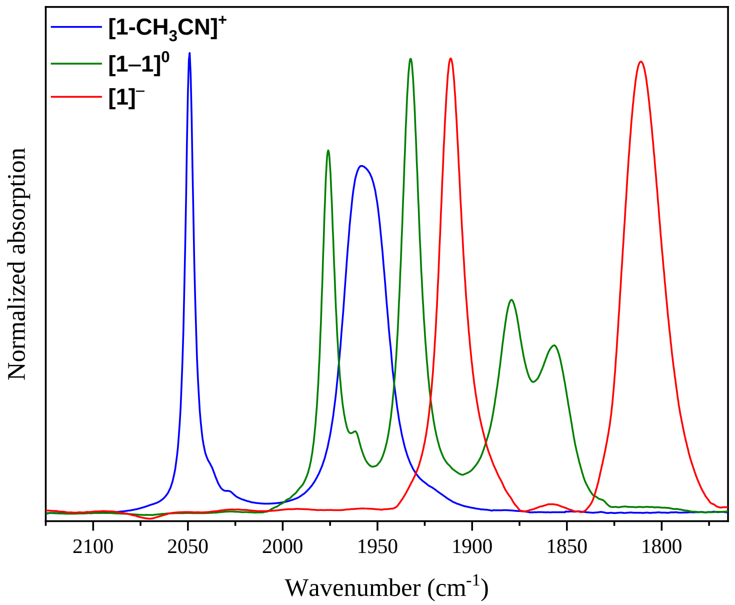

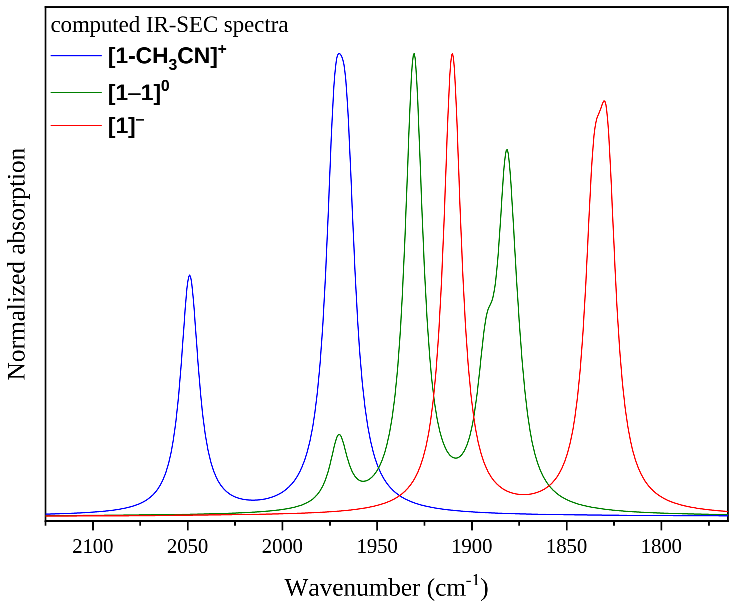

Figure S10. (top) Experimental IR-SEC spectra recorded on **$[1\text{-}CH_3CN]^+$** at the resting potential (blue), upon one-electron reduction (green) and upon two-electron reduction (red). Computed IR-SEC spectra are also provided (bottom).

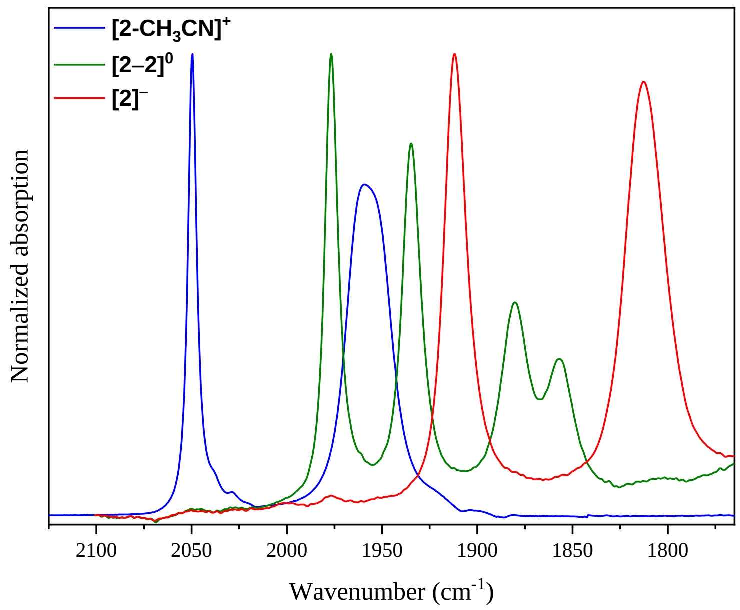

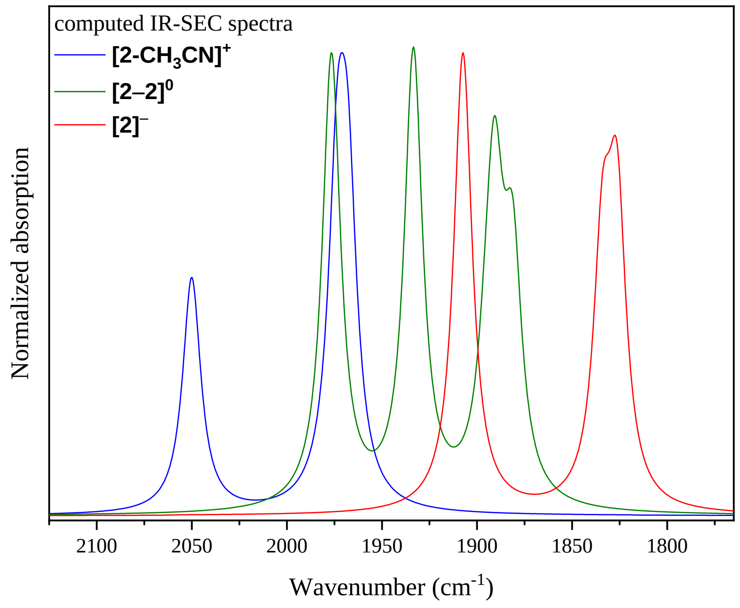

Figure S11. (top) Experimental IR-SEC spectra recorded on **[2-CH$_3$CN]$^+$** at the resting potential (blue), upon one-electron reduction (green) and upon two-electron reduction (red). Computed IR-SEC spectra are also provided (bottom).

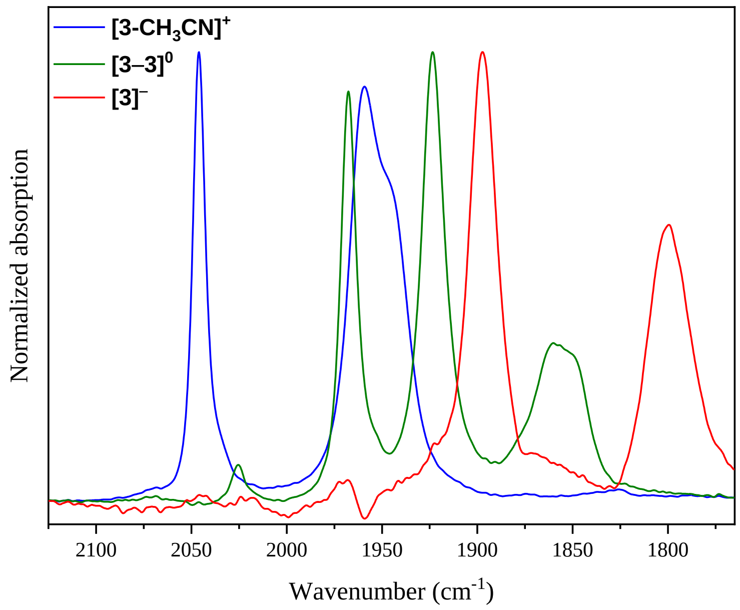

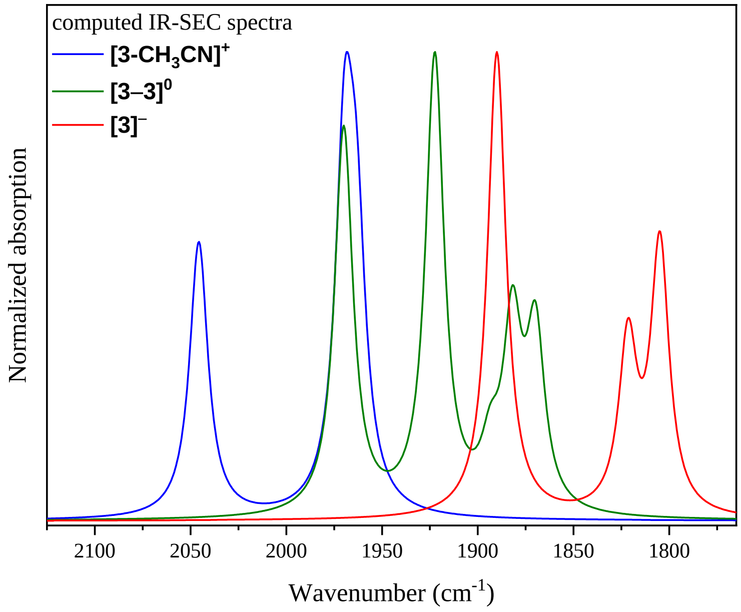

Figure S12. (top) Experimental IR-SEC spectra recorded on **[3-CH$_3$CN]$^+$** at the resting potential (blue), upon one-electron reduction (green) and upon two-electron reduction (red). Computed IR-SEC spectra are also provided (bottom).

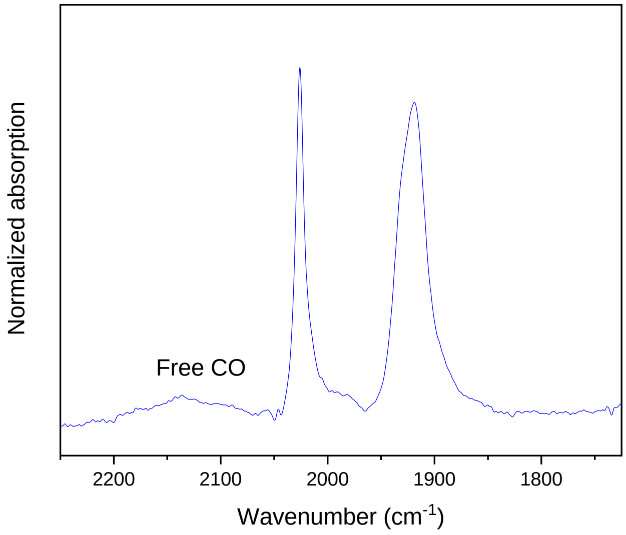

Figure S13. Minor decomposition products attributed to free CO and an unidentified species [ν(CO) = 1919 and 2026 cm^{-1}] observed from IR-SEC experiments upon one-electron reduction of **[3-CH_3CN]$^+$** to the **[3–3]^0** dimer.

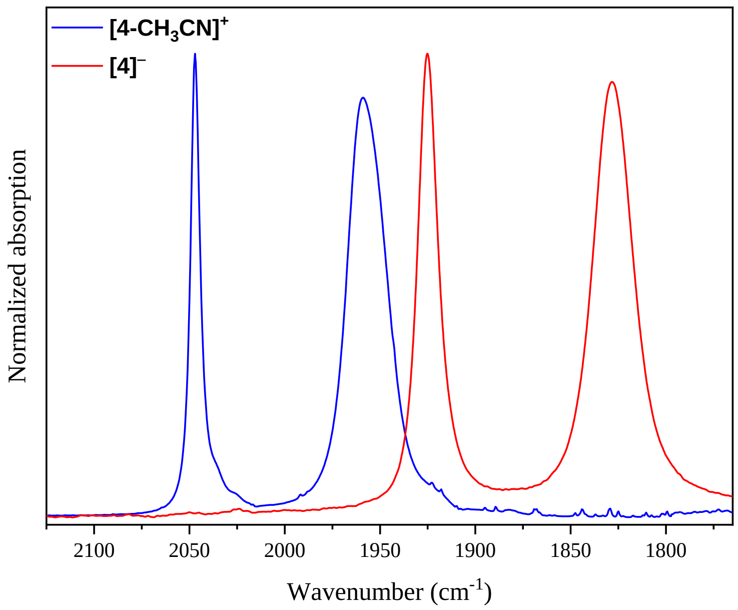

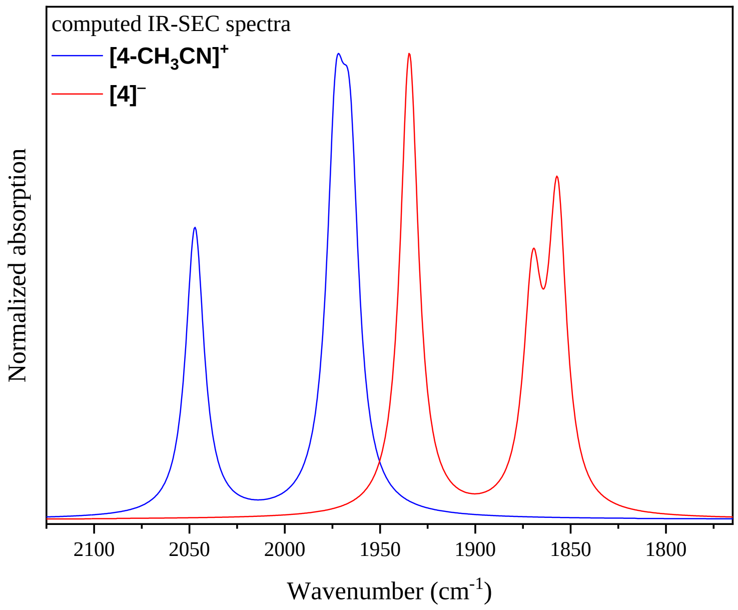

Figure S14. (top) Experimental IR-SEC spectra recorded on **[4-CH_3CN]$^+$** at the resting potential (blue) and upon two-electron reduction (red). Computed IR-SEC spectra are also provided (bottom).

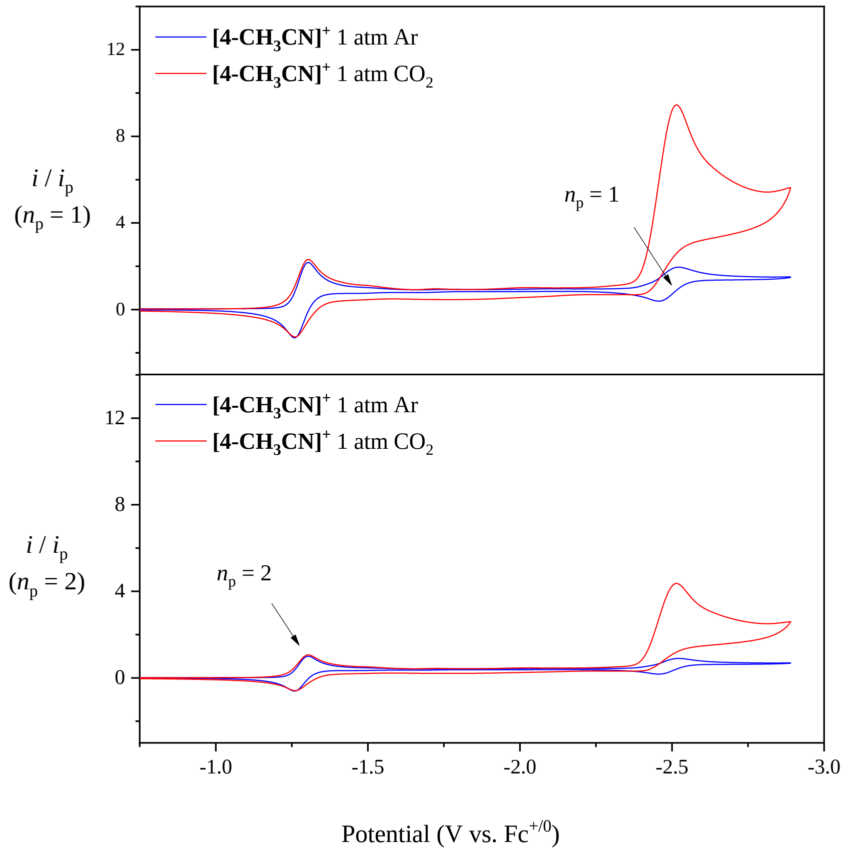

$n_p = 1$

$$\mathrm{TOF} = 0.1992\left(\frac{Fv}{RT}\right)\left(\frac{n_p^3}{n_{cat}^2}\right)\left(\frac{i_{cat}}{i_p}\right)^2 = 0.1992\left(\frac{F}{RT}\right)(0.1\ \mathrm{V\ s^{-1}})\left(\frac{1^3}{2^2}\right)(8.5)^2 = 14\ \mathrm{s^{-1}}$$

$n_p = 2$

$$\mathrm{TOF} = 0.1992\left(\frac{Fv}{RT}\right)\left(\frac{n_p^3}{n_{cat}^2}\right)\left(\frac{i_{cat}}{i_p}\right)^2 = 0.1992\left(\frac{F}{RT}\right)(0.1\ \mathrm{V\ s^{-1}})\left(\frac{2^3}{2^2}\right)(4.0)^2 = 25\ \mathrm{s^{-1}}$$

Figure S15. Cyclic voltammograms demonstrating catalytic activity of **[4-CH_3CN]$^+$** under 1 atm Ar (blue) and 1 atm CO_2 (red) at υ = 0.1 V s^{-1} with 0.1 M [Bu_4N][PF_6] acetonitrile supporting electrolyte and just residual 0.17 M (0.3%) H_2O as a Brønsted acid source. Both plots are derived from the same experimental data but with the current (y-axis) normalized with respect to the non-catalytic Faradaic response (i_p), where i_p corresponds to the one-electron reduction event ($n_p = 1$) at $E_{pc} = -2.51$ V or the concerted two-electron event ($n_p = 2$) at $E_{pc} = -1.30$ V. Calculation of TOF for **[4-CH_3CN]$^+$** using Eq. 3 is also included above using either the one-electron reduction event ($n_p = 1$) at $E_{pc} = -2.51$ V or the concerted two-electron reduction event ($n_p = 2$) at $E_{pc} = -1.30$ V for reference in determining i_{cat}/i_p. **Please see the main text for a discussion of the error in calculating TOF using $n_p = 2$ as a reference wave for the i_{cat}/i_p ratio.**

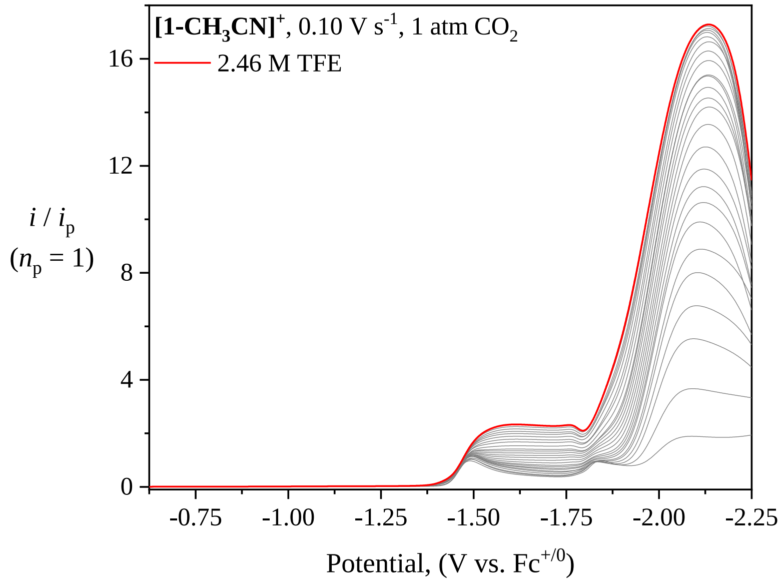

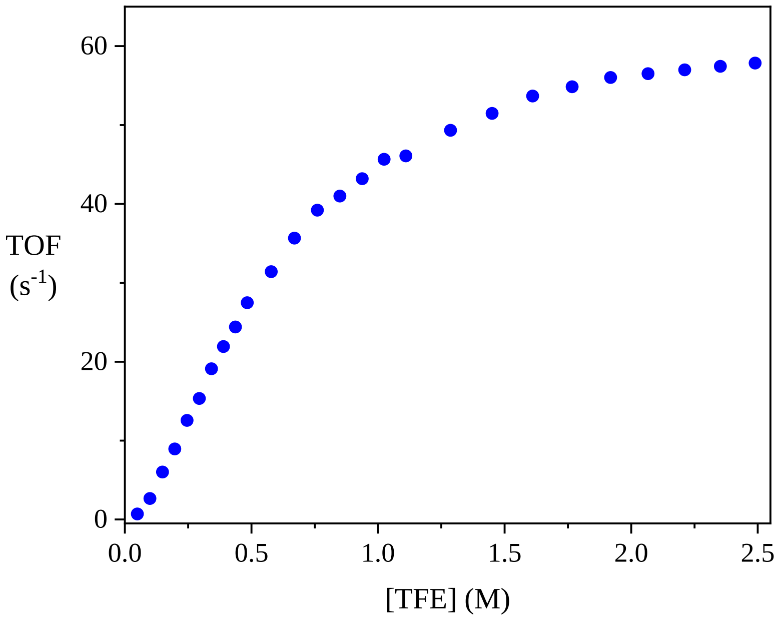

Figure S16. (top) Linear sweep voltammetry of **[1-CH3CN]**$^+$ at 0.1 V s^{-1} under 1 atm CO_2 with incremental TFE concentration up to 2.46 M TFE. (bottom) Plot of TOF vs [TFE] illustrating first-order dependence upon [TFE] as well as a plateau to zero-order dependence beyond 1.5 M TFE. (note: all voltammetry and TOF data have been corrected for [Mn] dilution due to TFE addition).

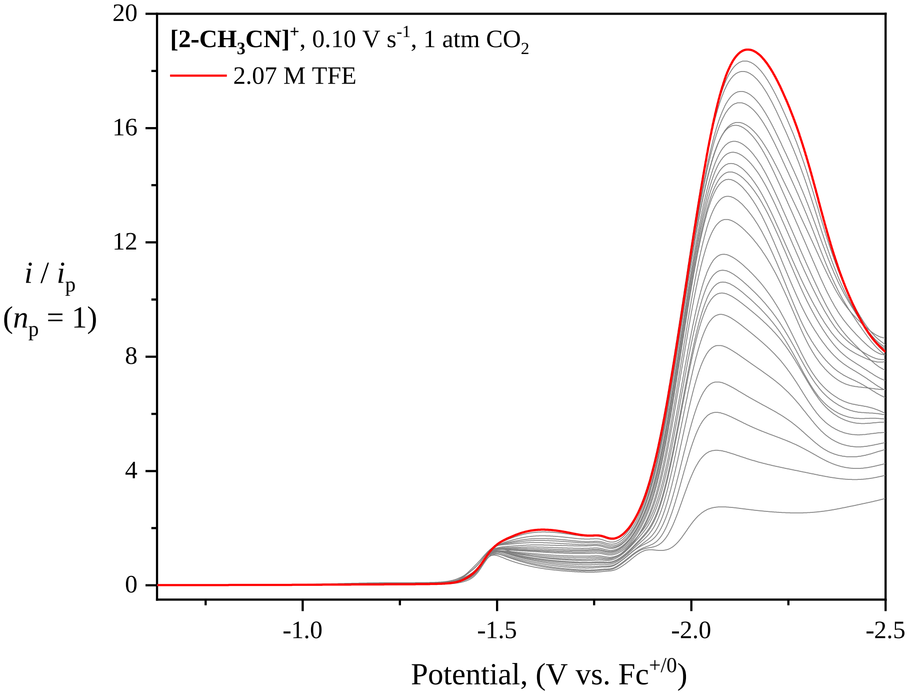

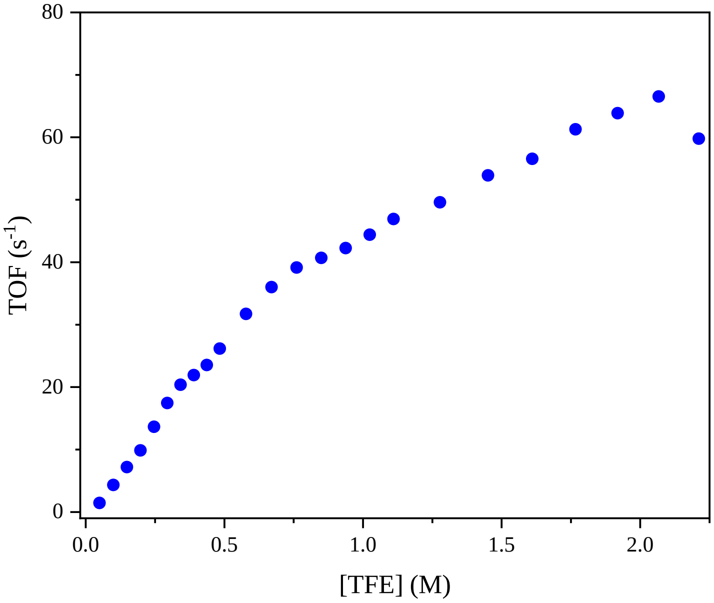

Figure S17. (top) Linear sweep voltammetry of **[2-CH_3CN]$^+$** at 0.1 V s^{-1} under 1 atm CO_2 with incremental TFE concentration up to 2.07 M TFE. (bottom) Plot of TOF vs [TFE] plateauing at *ca.* 2.0 M TFE. (note: all voltammetry and TOF data have been corrected for [Mn] dilution due to TFE addition).

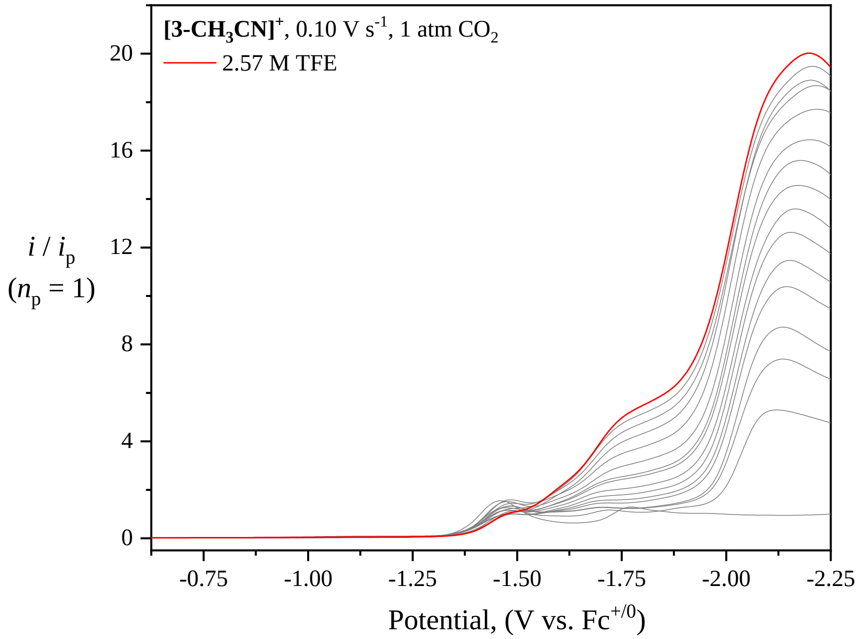

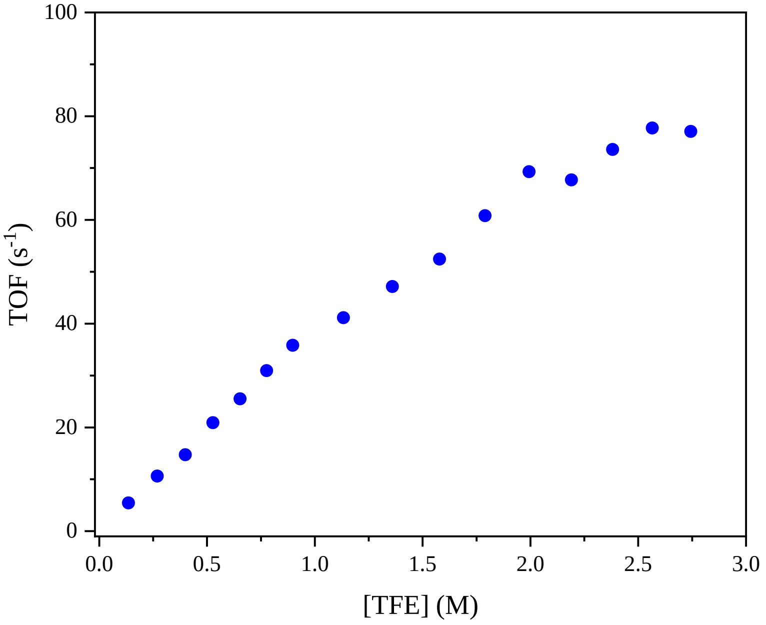

Figure S18. (top) Linear sweep voltammetry of **[3-CH3CN]**$^+$ at 0.1 V s^{-1} under 1 atm CO_2 with incremental TFE concentration up to 2.57 M TFE. (bottom) Plot of TOF vs [TFE] plateauing at *ca.* 2.5 M TFE. (note: all voltammetry and TOF data have been corrected for [Mn] dilution due to TFE addition).

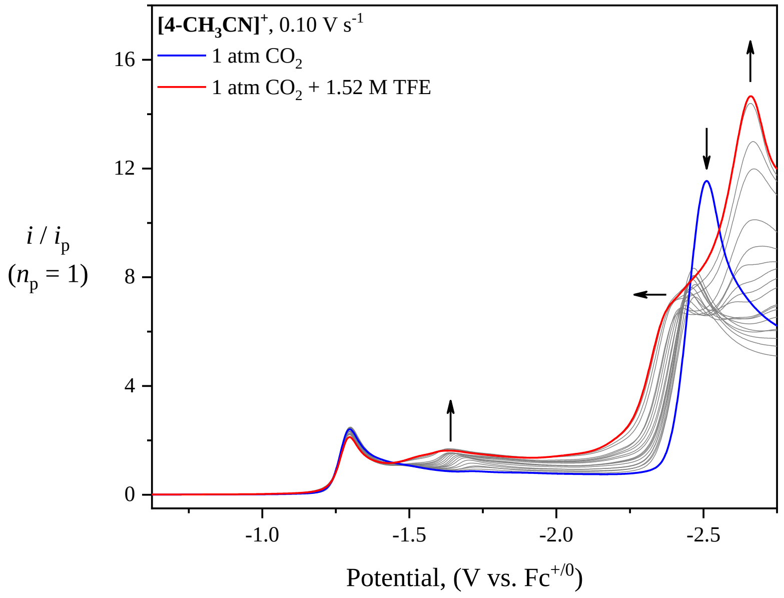

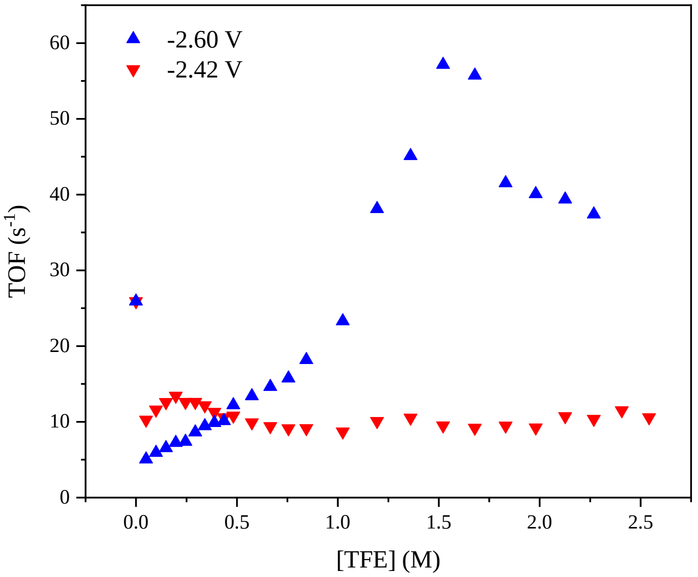

Figure S19. (top) Linear sweep voltammetry of **[4-CH3CN]**$^+$ at 0.1 V s^{-1} under 1 atm CO_2 with incremental TFE concentration up to 1.52 M TFE. (bottom) Plot of TOF vs [TFE] illustrating first-order dependence upon [TFE] at –2.60 V as well as a plateau current at *ca.* 1.5 M TFE. Also plotted are TOF data calculated at –2.42 V, which exhibit only a weak dependence upon TFE concentration. (note: all voltammetry and TOF data have been corrected for [Mn] dilution due to TFE addition).

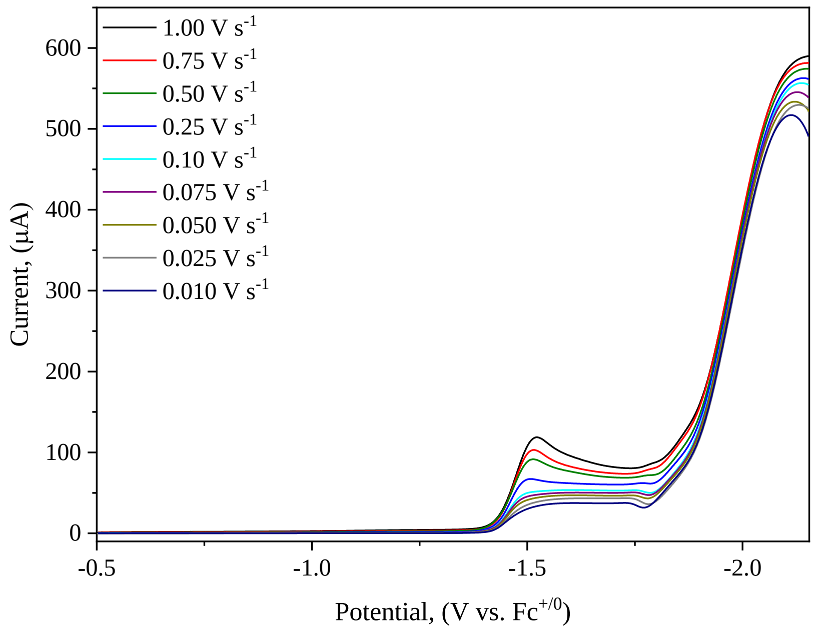

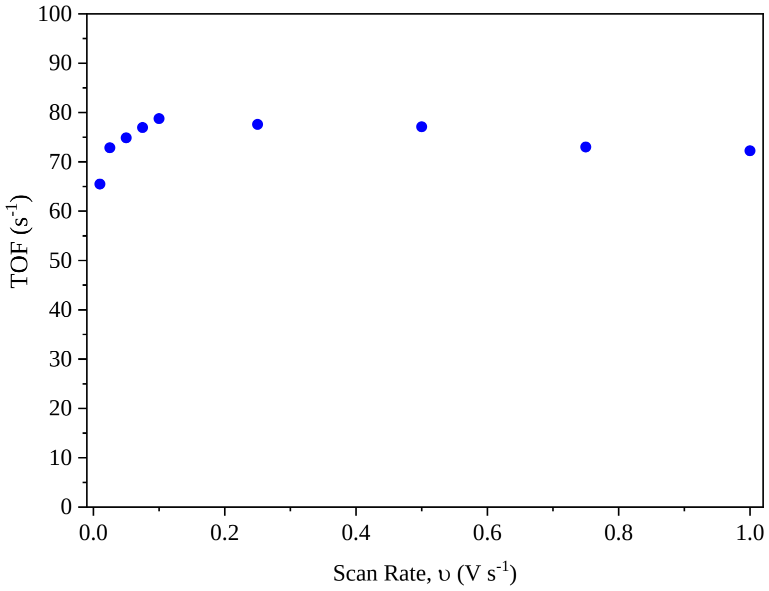

Figure S20. Scan rate studies of **[1-CH_3CN]$^+$** under 1 atm CO_2 with 2.0 M TFE to demonstrate steady state catalysis. (top) Cyclic voltammograms at varying scan rates and (bottom) a plot of 'TOF vs. scan rate' to illustrate the steady-state catalysis being achieved from 0.025 V s^{-1} up to 1.0 V s^{-1}.

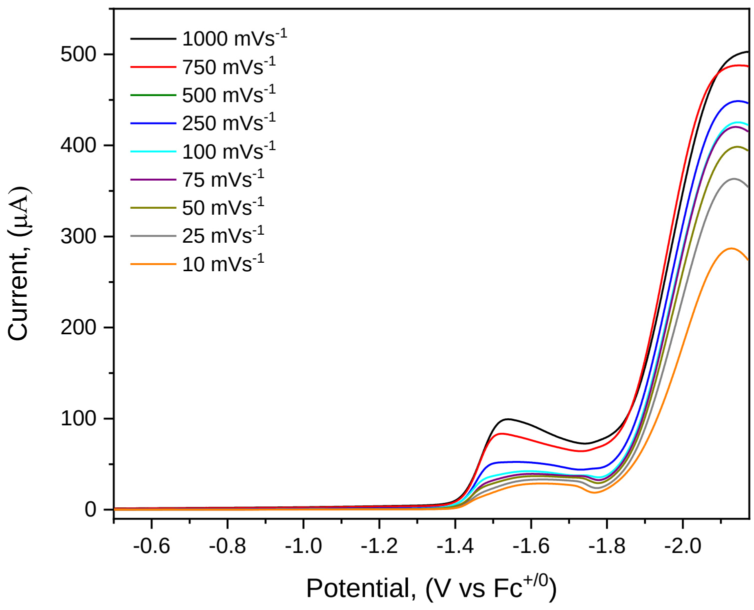

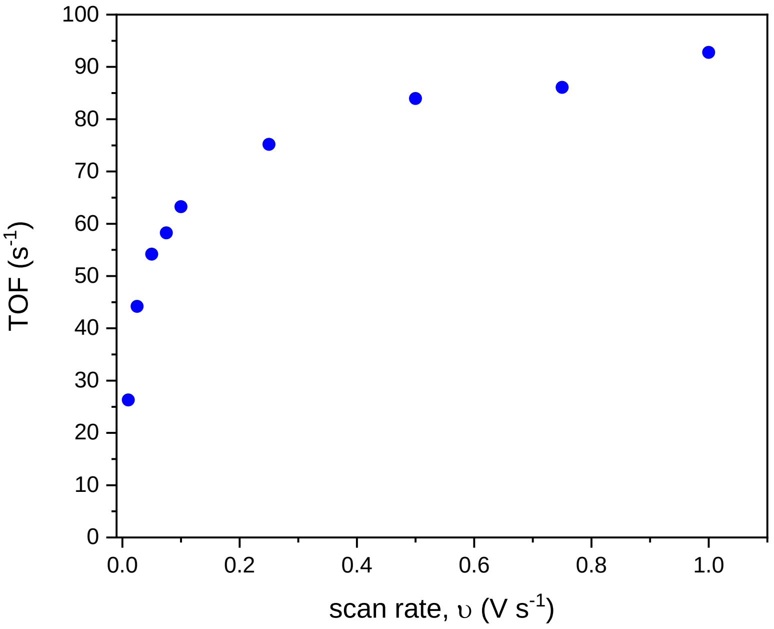

Figure S21. Scan rate studies of **[2-CH_3CN]$^+$** under 1 atm CO_2 with 2.0 M TFE to demonstrate steady state catalysis. (top) Cyclic voltammograms at varying scan rates and (bottom) a plot of 'TOF vs. scan rate' to illustrate the steady-state catalysis being achieved from *ca.* 0.25 V s^{-1} up to 1.0 V s^{-1}.

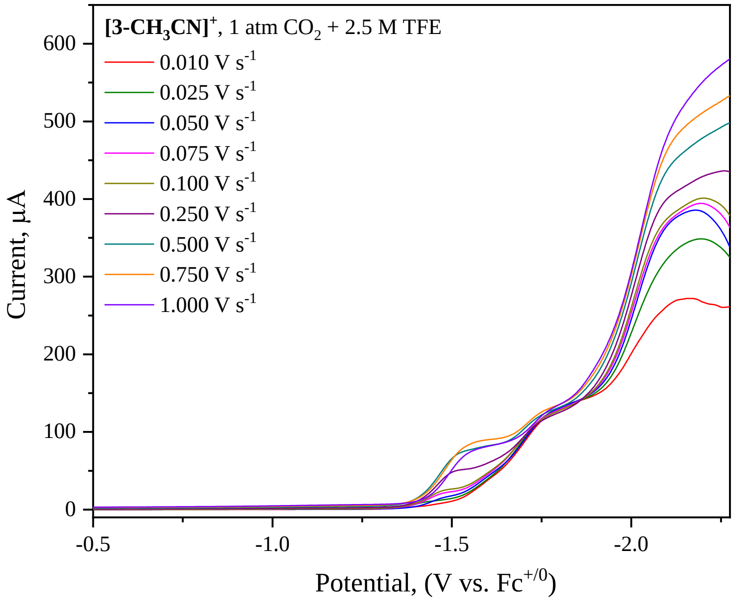

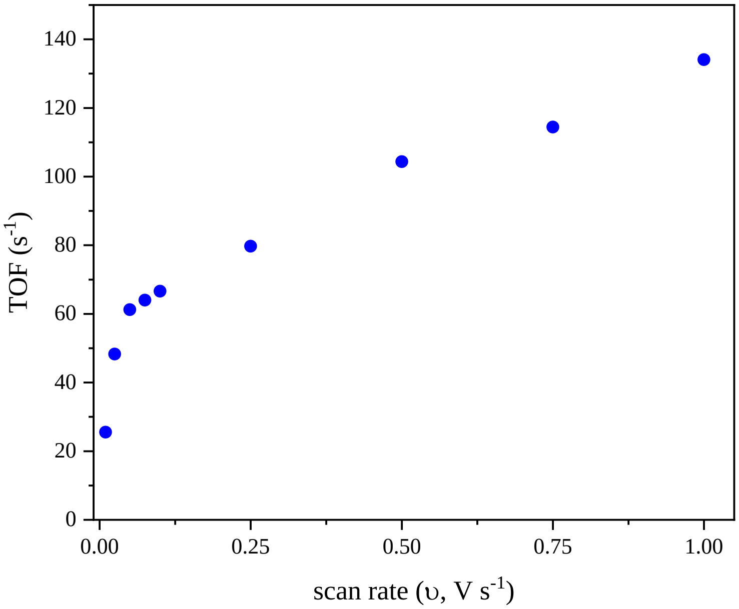

Figure S22. Scan rate studies of **[3-CH_3CN]$^+$** under 1 atm CO_2 with 2.5 M TFE. (top) Cyclic voltammograms at varying scan rates and (bottom) a plot of ‘TOF vs. scan rate’. Note, TOF appears to plateau at 67 s^{-1} (υ = 0.1 V s^{-1}), however observation of a weaker slope upon increasing the scan rate prevents confirmation of steady-state conditions and is suggestive of a secondary non-catalytic scan-rate dependent current.

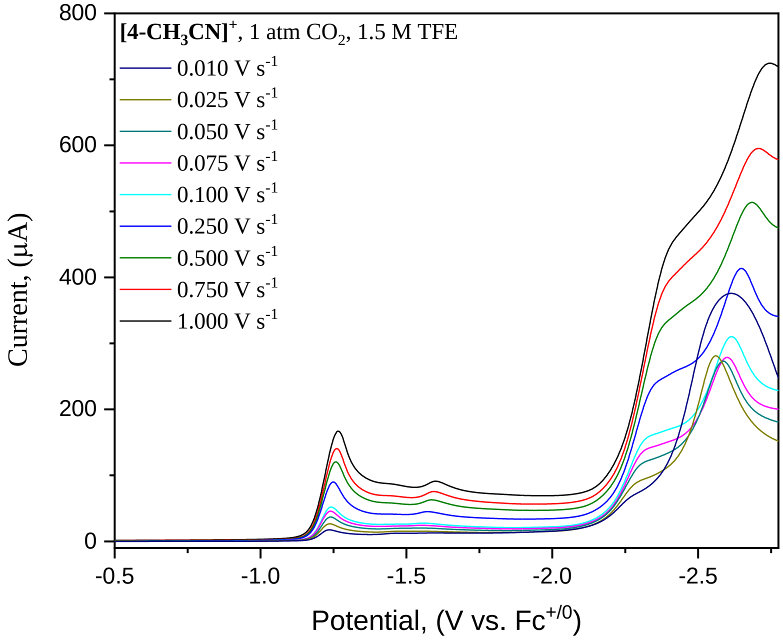

Figure S23. Cyclic voltammetry of **[4-CH_3CN]$^+$** under 1 atm CO_2 with 1.5 M TFE at varying scan rates of 0.01 – 0.1 V s^{-1}.

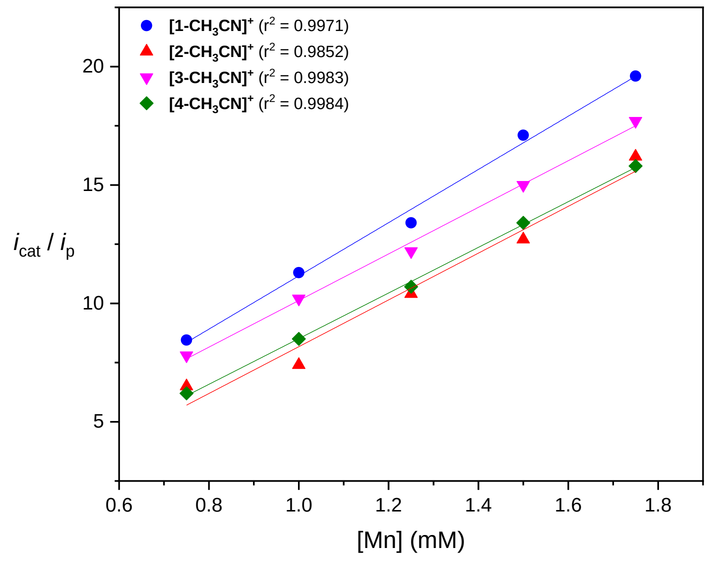

Figure S24. Catalyst concentration studies for each complex under 1 atm CO_2 with residual 0.17 M (0.3%) H_2O as a Brønsted acid source. Recorded in 0.1 M [Bu_4N][PF_6] acetonitrile supporting electrolyte at 0.1 V s^{-1}.

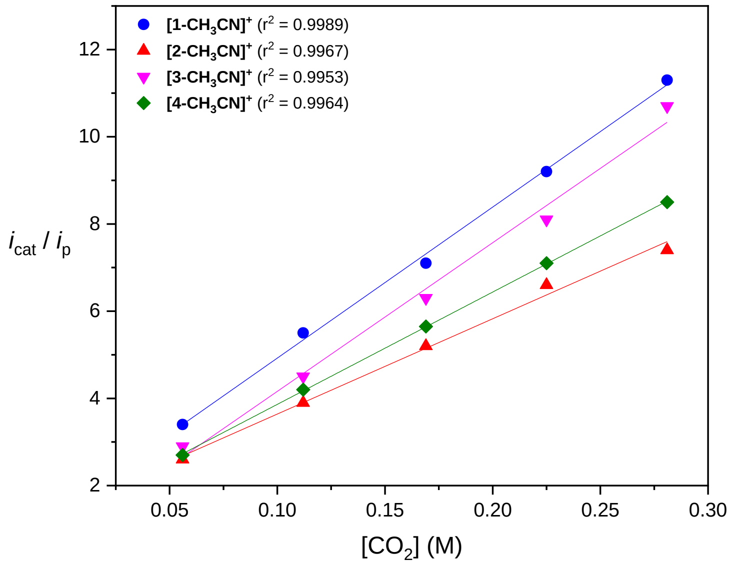

Figure S25. CO_2 concentration studies for each complex with residual 0.17 M (0.3%) H_2O as a Brønsted acid source. Recorded in 0.1 M [Bu_4N][PF_6] acetonitrile supporting electrolyte at 0.1 V s^{-1}.

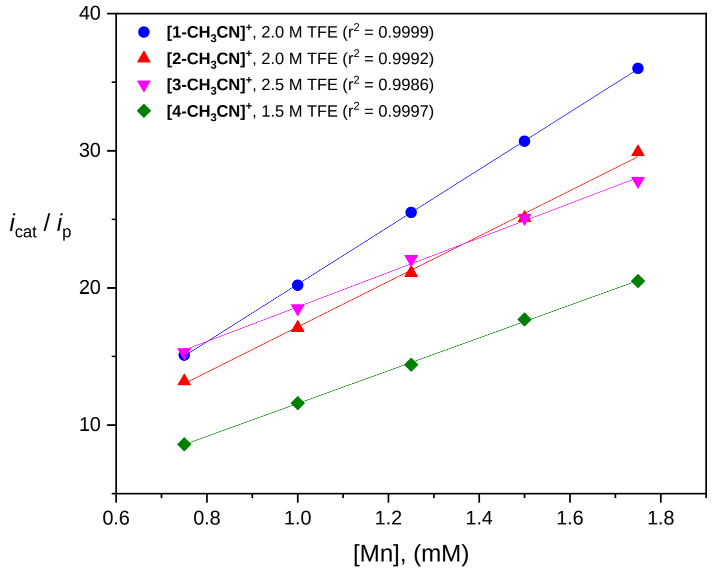

Figure S26. Catalyst concentration studies for each complex under 1 atm CO_2 with optimized TFE concentrations. Recorded in 0.1 M [Bu_4N][PF_6] acetonitrile supporting electrolyte at 0.1 V s^{-1}.

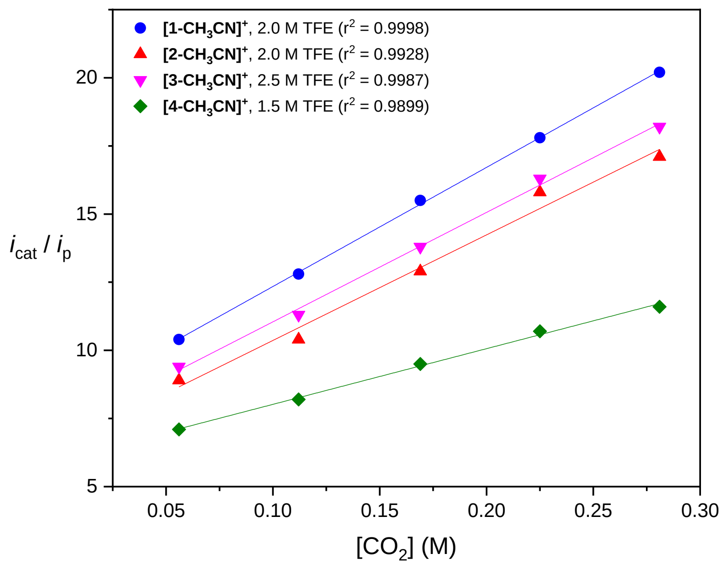

Figure S27. CO_2 concentration studies for each complex with optimized TFE concentrations. Recorded in 0.1 M [Bu_4N][PF_6] acetonitrile supporting electrolyte at 0.1 V s^{-1}.

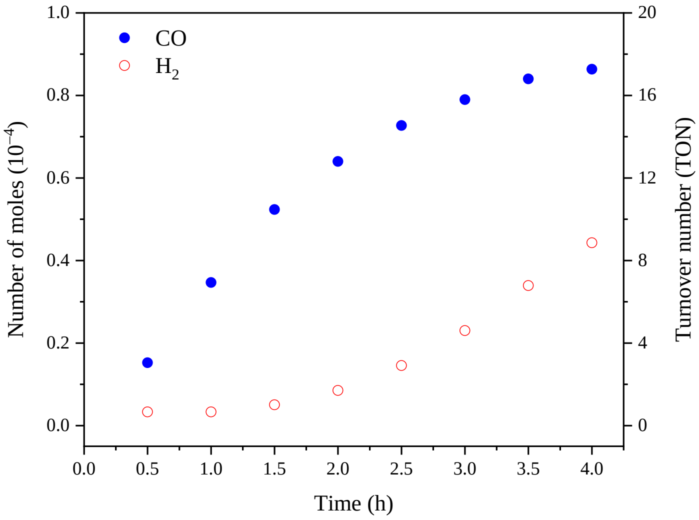

Figure S28. Controlled potential electrolysis data of **[1-CH3CN]**$^{+}$ showing the number of moles (left y-axis) and turnover number (TON, right y-axis) with residual 0.17 M (0.3%) H_2O as a Brønsted acid source.

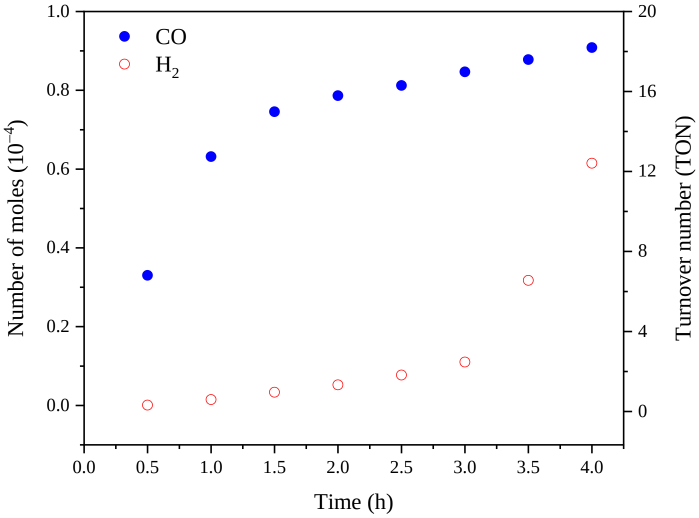

Figure S29. Controlled potential electrolysis data of **[1-CH3CN]**$^{+}$ showing the number of moles (left y-axis) and turnover number (TON, right y-axis) in the presence of 2.0 M TFE.

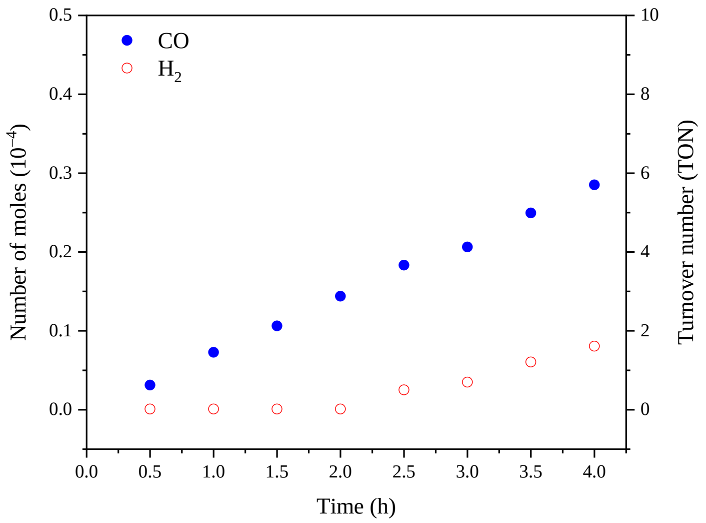

Figure S30. Controlled potential electrolysis data of **[2-CH3CN]**$^+$ showing the number of moles (left y-axis) and turnover number (TON, right y-axis) with residual 0.17 M (0.3%) H_2O as a Brønsted acid source.

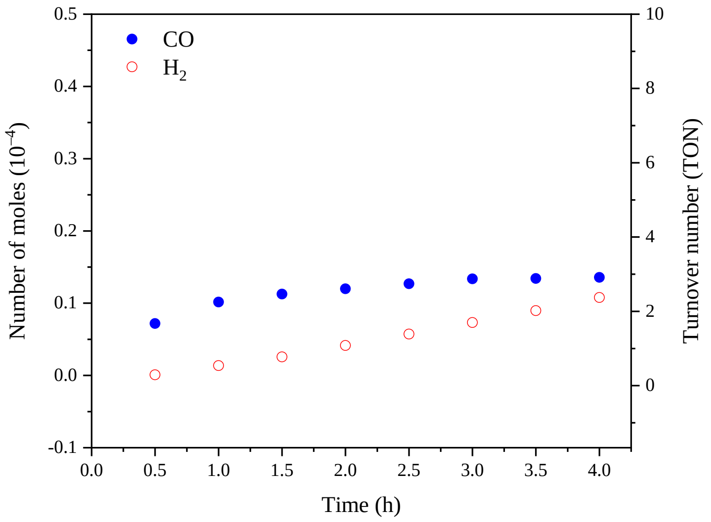

Figure S31. Controlled potential electrolysis data of **[2-CH3CN]**$^+$ showing the number of moles (left y-axis) and turnover number (TON, right y-axis) in the presence of 2.0 M TFE.

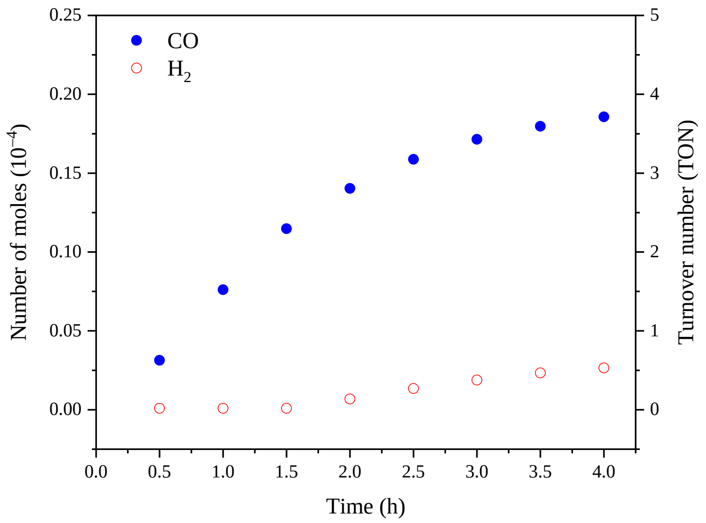

Figure S32. Controlled potential electrolysis data of **[3-CH3CN]**$^+$ showing the number of moles (left y-axis) and turnover number (TON, right y-axis) with residual 0.17 M (0.3%) H_2O as a Brønsted acid source.

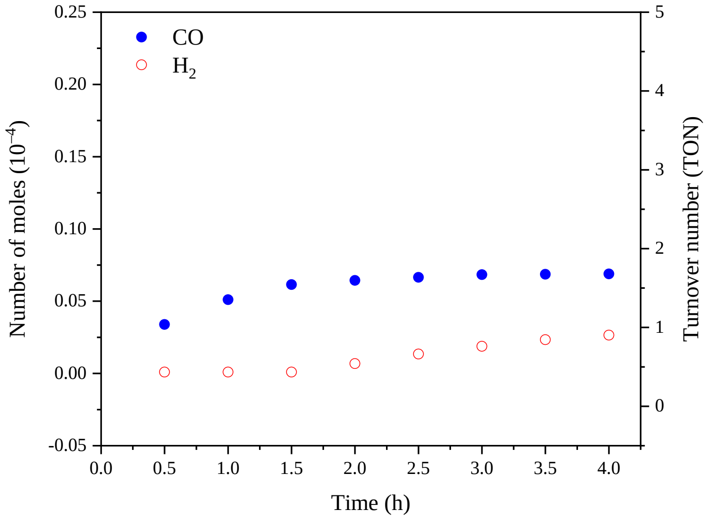

Figure S33. Controlled potential electrolysis data of **[3-CH3CN]**$^+$ showing the number of moles (left y-axis) and turnover number (TON, right y-axis) in the presence of 2.5 M TFE.

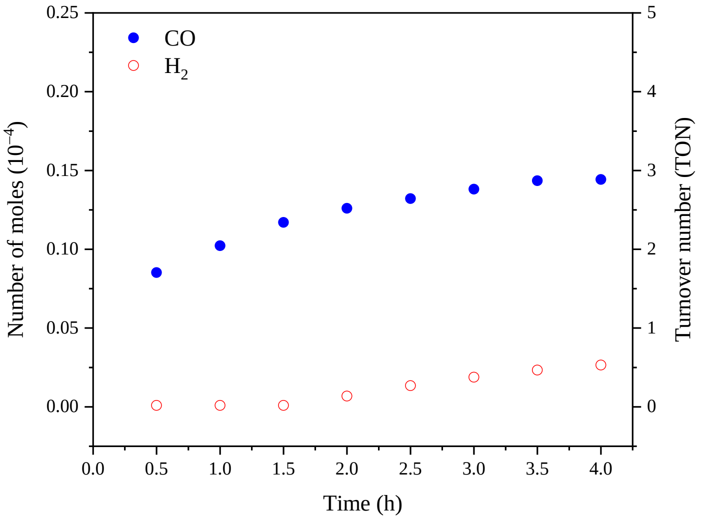

Figure S34. Controlled potential electrolysis data of **[4-CH3CN]**$^+$ showing the number of moles (left y-axis) and turnover number (TON, right y-axis) with residual 0.17 M (0.3%) H_2O as a Brønsted acid source.

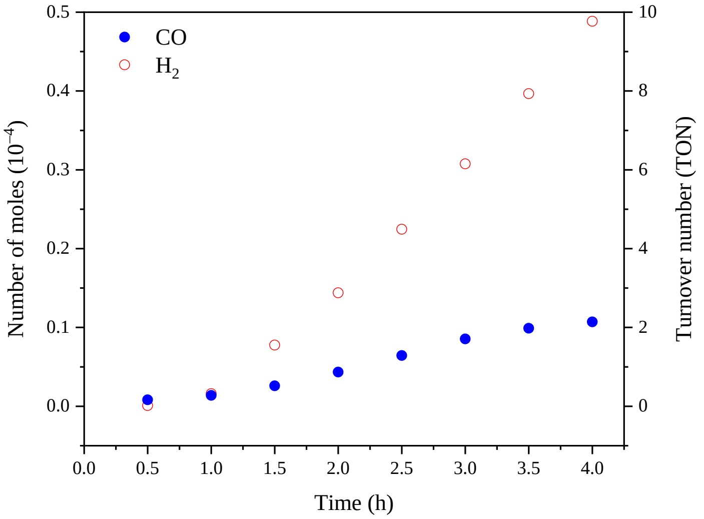

Figure S35. Controlled potential electrolysis data of **[4-CH3CN]**$^+$ showing the number of moles (left y-axis) and turnover number (TON, right y-axis) in the presence of 1.5 M TFE.

[1-CH_3CN]^0 **[1]^0**

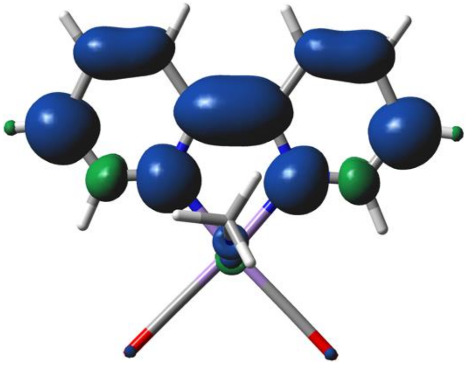

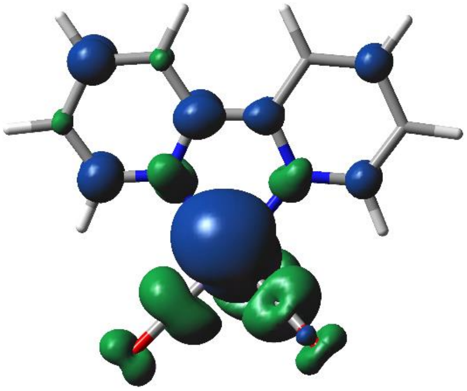

[2-CH_3CN]^0 **[2]^0**

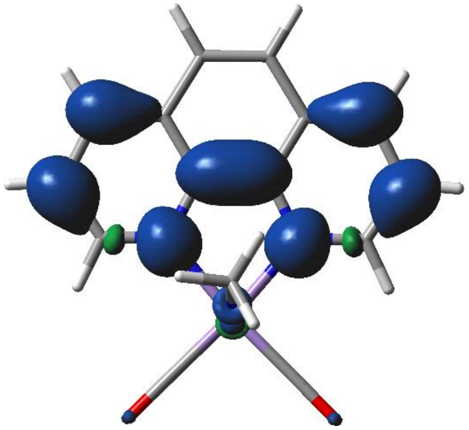

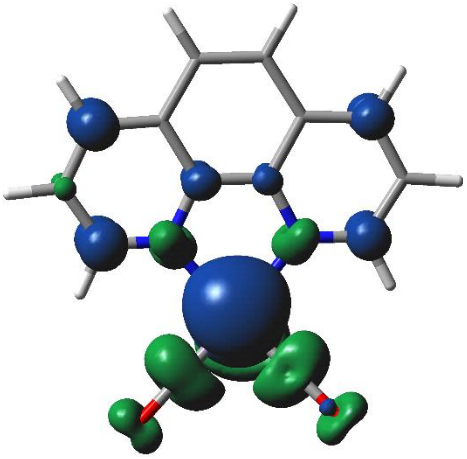

[4-CH_3CN]^0 **[4]^0**

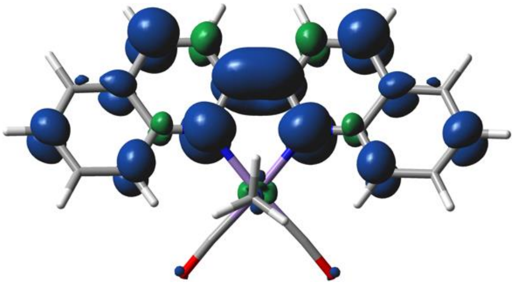

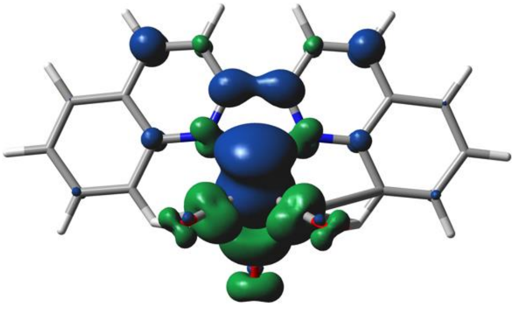

Figure S36. Spin density (σ) plots (isovalue = 0.002) for **[Mn-CH_3CN]^0** and **[Mn]^0** complexes at the M06 level of theory. Alpha spin density (σ_α) in blue color and beta spin density (σ_β) in green color.

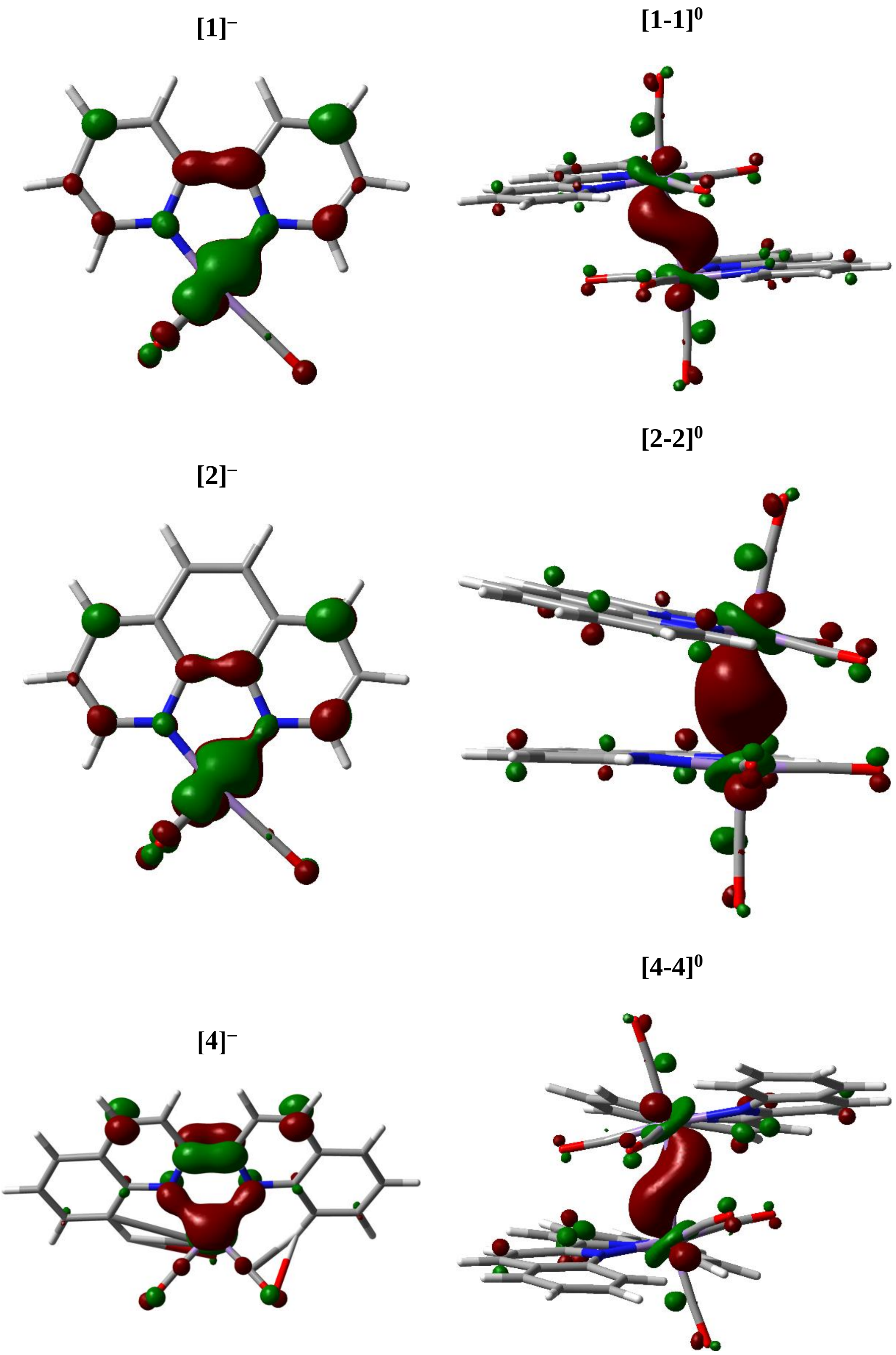

Figure S37. Plots (isovalue = 0.05) of highest occupied molecular orbitals (HOMOs) for **[Mn]$^{-}$** and **[Mn-Mn]^{0}** complexes at the M06 level of theory.

[1]$^{2-}$

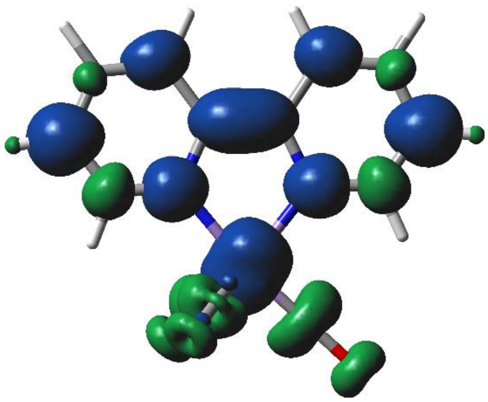

[2]$^{2-}$

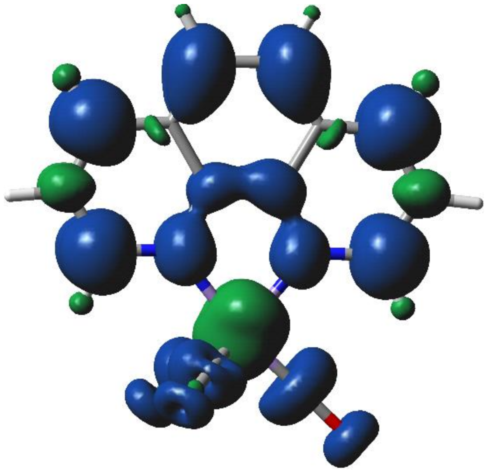

[4]$^{2-}$

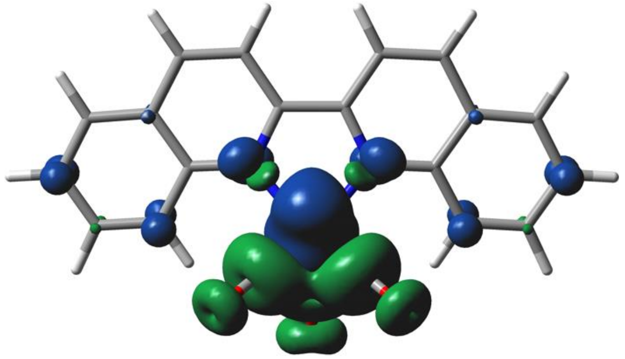

Figure S38. Spin density (σ) plots (isovalue = 0.002) for **[Mn]$^{2-}$** complexes at the M06 level of theory. Alpha spin density (σ_{α}) in blue color and beta spin density (σ_{β}) in green color.

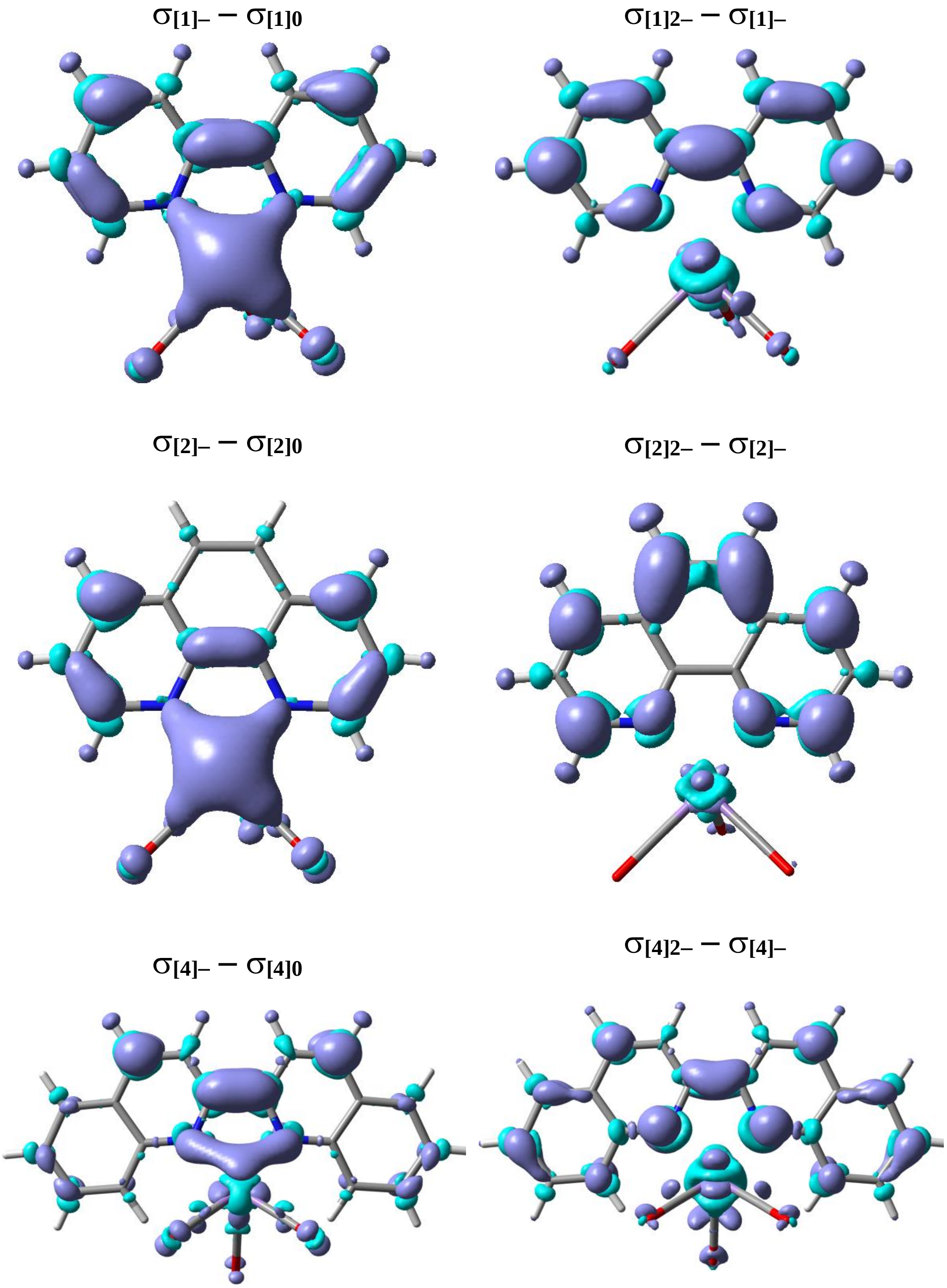

Figure S39. Total electron density (σ_{tot}) difference plots (isovalue = 0.002) for **[Mn]**$^{-}$ and **[Mn]**^{0} complexes ($\sigma_{[Mn]-} - \sigma_{[Mn]0}$), and **[Mn]**$^{2-}$ and **[Mn]**$^{-}$ complexes ($\sigma_{[Mn]2-} - \sigma_{[Mn]-}$), at the M06 level of theory.

Optimized Coordinates at M06 Level of Theory

CH_3CN

N	1.43544	-0.00005	-0.00001
C	0.27401	0.00011	0.00004
C	-1.17343	-0.00001	0.00001
H	-1.55041	0.53729	-0.87726
H	-1.55035	-1.02848	-0.02688
H	-1.55078	0.49087	0.90390

CO_2

C	0.00000	0.00000	-0.00000
O	0.00000	0.00000	-1.16466
O	0.00000	0.00000	1.16466

CO

C	0.00000	0.00000	-0.64986
O	0.00000	0.00000	0.48739

CF_3CH_2OH (TFE)

O	1.91042	-0.22307	0.00005
H	2.76280	0.23833	-0.00036
C	0.90106	0.74355	0.00005
H	0.91582	1.39033	0.89180
H	0.91593	1.39037	-0.89168
C	-0.41299	0.01505	-0.00000
F	-0.55435	-0.76678	-1.07631
F	-1.42545	0.89100	0.00018
F	-0.55424	-0.76711	1.07608

$CF_3CH_2O^-$ (TFE-H)

O	2.01745	-0.18603	0.00028
C	1.03313	0.67000	-0.00087
H	0.94062	1.39315	0.88604
H	0.94102	1.39105	-0.88945
C	-0.34709	0.02088	-0.00010
F	-0.55021	-0.76711	-1.07510
F	-1.35841	0.92478	-0.00231
F	-0.55111	-0.76225	1.07817

$[1\text{-}CH_3CN]^+$

$[1\text{-}CH_3CN]^+$

Mn	-1.06586	-0.01136	-0.30735
C	-1.07244	-0.04703	-2.10131
C	-2.35557	1.24477	-0.28415
C	-2.34184	-1.27937	-0.22892
O	-1.05585	-0.06879	-3.25477
O	-3.19204	2.04108	-0.26776
O	-3.16872	-2.08394	-0.17496
N	-0.90087	0.02954	1.70528
C	-0.78958	0.05194	2.85569
C	-0.65363	0.07934	4.29142
H	0.24517	-0.47150	4.58984
H	-0.57116	1.11583	4.63654
H	-1.53060	-0.38518	4.75552
C	1.75226	-0.72890	-0.15791
C	0.45001	-2.64637	-0.27143
C	2.91280	-1.49187	-0.07082
C	1.74338	0.74043	-0.17238
C	1.56516	-3.46658	-0.18840
H	-0.54551	-3.07512	-0.35833
C	2.81746	-2.87646	-0.08401
H	3.88608	-1.01508	0.00211
C	2.89258	1.51897	-0.07619
H	1.44110	-4.54566	-0.20755
H	3.71583	-3.48564	-0.01784
C	0.42158	2.63933	-0.35162
C	2.78211	2.90168	-0.12387
H	3.86826	1.05520	0.03670
C	1.52585	3.47431	-0.26948
H	-0.57719	3.05420	-0.46508
H	3.67174	3.52281	-0.05103
H	1.39008	4.55108	-0.31781
N	0.52103	1.30530	-0.29992
N	0.53482	-1.31038	-0.25382

$[1\text{-}CH_3CN]^0$

Mn	1.06698	-0.00064	-0.31696
C	1.03527	-0.00206	-2.09380
C	2.33923	-1.26665	-0.27897
C	2.34011	1.26454	-0.28093
O	0.97911	-0.00300	-3.24976
O	3.17191	-2.07261	-0.26354
O	3.17338	2.06991	-0.26691
N	0.91800	0.00119	1.73485
C	0.80535	0.00186	2.88577
C	0.66650	0.00241	4.32296
H	-0.23120	0.56097	4.60996
H	0.57999	-1.02663	4.68866
H	1.54314	0.47296	4.78151
C	-1.74381	0.70943	-0.15015
C	-0.42755	2.65251	-0.31279
C	-2.90513	1.52120	-0.06333
C	-1.74453	-0.70822	-0.15021
C	-1.52432	3.48354	-0.23381
H	0.57165	3.07509	-0.41787
C	-2.80153	2.88677	-0.10113
H	-3.88123	1.04968	0.03271
C	-2.90690	-1.51880	-0.06633
H	-1.39479	4.56176	-0.27208

H	-3.69492	3.50540	-0.03380
C	-0.42971	-2.65267	-0.30787
C	-2.80441	-2.88453	-0.10178
H	-3.88304	-1.04630	0.02435
C	-1.52732	-3.48265	-0.22944
H	0.56930	-3.07629	-0.41049
H	-3.69862	-3.50223	-0.03694
H	-1.39861	-4.56105	-0.26527
N	-0.50089	-1.31067	-0.26369
N	-0.49979	1.31064	-0.26605

[1]^0

Mn	1.12023	0.16211	-0.08776
C	1.80153	-0.21751	1.54339
C	2.21039	1.54778	-0.30130
C	2.29923	-0.81869	-1.00868
O	2.28882	-0.49574	2.56704
O	2.94232	2.45039	-0.43427
O	3.08930	-1.45765	-1.58645
C	-1.61110	-0.87177	-0.06803
C	-0.07849	-2.61374	-0.03962
C	-2.67359	-1.77498	-0.05026
C	-1.77449	0.58386	-0.06516
C	-1.08419	-3.56379	-0.01639
H	0.96923	-2.90789	-0.03032
C	-2.40847	-3.13479	-0.02740
H	-3.70056	-1.41984	-0.05111
C	-3.01326	1.22206	-0.06677
H	-0.82772	-4.61948	0.00771
H	-3.22636	-3.85170	-0.01404
C	-0.67758	2.62711	-0.02199
C	-3.06517	2.60701	-0.04554
H	-3.93201	0.64237	-0.08374
C	-1.87295	3.32445	-0.01918
H	0.27203	3.15693	-0.00311
H	-4.02429	3.11972	-0.04781
H	-1.86213	4.41095	0.00048
N	-0.61175	1.28385	-0.04752
N	-0.32377	-1.29269	-0.07148

[1_2]^0

Mn	0.902489	-0.449840	-1.171233
C	1.660087	-0.525924	-2.758693
C	-0.590712	0.222469	-1.850270
C	0.186350	-2.063150	-1.376297
O	2.088407	-0.594066	-3.842794
O	-1.528832	0.651728	-2.401770
O	-0.277266	-3.111939	-1.601856
C	3.413508	-0.034128	0.235335
C	2.956084	-2.302589	0.064904
C	4.632078	-0.305203	0.861241
C	2.930748	1.310418	-0.054002
C	4.144986	-2.638480	0.683977
H	2.269489	-3.076327	-0.270610
C	5.002963	-1.617468	1.093049
H	5.284745	0.509988	1.162782
C	3.616538	2.477069	0.289244
H	4.392263	-3.685610	0.838766
H	5.947834	-1.844867	1.581563
C	1.218272	2.555872	-1.003809
C	3.067151	3.708899	-0.020622
H	4.572968	2.417934	0.802437
C	1.841770	3.747894	-0.685571
H	0.265347	2.553698	-1.529707
H	3.586576	4.626390	0.246781
H	1.368809	4.688948	-0.956275
N	1.730586	1.350050	-0.688381
N	2.575482	-1.028960	-0.155896
Mn	-0.902216	-0.447588	1.172300
C	-1.659209	-0.522085	2.760148
C	0.590409	0.227503	1.849748
C	-0.183824	-2.059572	1.379582
O	-2.086930	-0.589141	3.844562
O	1.528406	0.658622	2.400026
O	0.281390	-3.107286	1.606896
C	-3.413227	-0.037328	-0.235915
C	-2.952916	-2.304910	-0.061905
C	-4.630939	-0.310926	-0.862402
C	-2.932428	1.308285	0.051738
C	-4.140713	-2.643273	-0.681742
H	-2.265620	-3.077251	0.275383
C	-4.999789	-1.623993	-1.092845
H	-5.284484	0.502979	-1.165481
C	-3.619473	2.473532	-0.293802
H	-4.386387	-3.690950	-0.835391
H	-5.943943	-1.853340	-1.581834
C	-1.222859	2.557426	1.001909
C	-3.072366	3.706550	0.015403
H	-4.575165	2.412395	-0.808145
C	-1.847916	3.748160	0.681873
H	-0.270476	2.557286	1.528764
H	-3.592856	4.622945	-0.253683
H	-1.376796	4.690229	0.952242
N	-1.732909	1.350469	0.687183
N	-2.574295	-1.030469	0.157608

[1]$^−$

Mn	1.11128	0.15310	-0.00469
C	1.97741	-0.54561	1.36213
C	2.20091	1.54357	-0.02918
C	2.02103	-0.60733	-1.31016
O	2.60985	-1.04584	2.22037

O	2.93394	2.46314	-0.04541
O	2.67521	-1.14681	-2.12728
C	-1.62344	-0.81826	-0.00640
C	-0.09757	-2.59097	-0.00364
C	-2.69353	-1.73811	-0.00483
C	-1.75442	0.60054	-0.00683
C	-1.10083	-3.52569	-0.00225
H	0.94974	-2.89160	-0.00224
C	-2.44279	-3.08789	-0.00334
H	-3.71744	-1.36915	-0.00498
C	-2.98382	1.28971	-0.00227
H	-0.85100	-4.58414	-0.00039
H	-3.26181	-3.80439	-0.00262
C	-0.60951	2.63625	-0.00862
C	-3.01263	2.66179	-0.00085
H	-3.91151	0.72014	0.00082
C	-1.77756	3.34926	-0.00417
H	0.34754	3.15432	-0.01119
H	-3.95588	3.20365	0.00301
H	-1.73712	4.43654	-0.00324
N	-0.54107	1.26801	-0.01022
N	-0.31834	-1.24805	-0.00706

[1-H]^0

Mn	1.15721	-0.00659	-0.18861
C	1.40396	0.01638	1.60818
C	2.38894	1.22603	-0.51350
C	2.38013	-1.25628	-0.47955
O	1.62653	0.03202	2.74741
O	3.19953	2.02170	-0.76649
O	3.18500	-2.06462	-0.70965
C	-1.67340	-0.72853	-0.10005
C	-0.36415	-2.63874	-0.09380
C	-2.83679	-1.49584	-0.08894
C	-1.66761	0.73784	-0.09744
C	-1.47832	-3.46244	-0.07376
H	0.63684	-3.06420	-0.09717
C	-2.73942	-2.87864	-0.07673
H	-3.81331	-1.01965	-0.09253
C	-2.82427	1.51394	-0.05212
H	-1.34855	-4.54123	-0.05957
H	-3.63803	-3.49121	-0.06787
C	-0.34419	2.63813	-0.12486
C	-2.71643	2.89600	-0.04813
H	-3.80355	1.04496	-0.01589
C	-1.45149	3.47035	-0.08488
H	0.65970	3.05586	-0.15083
H	-3.60979	3.51532	-0.01409
H	-1.31337	4.54818	-0.08097
N	-0.43814	1.30082	-0.13410
N	-0.44777	-1.30082	-0.11238
H	0.95081	-0.02533	-1.79708

[1]$^{2-}$

Mn	1.17931	-0.01163	-0.00618
C	1.86977	-0.70413	1.46671
C	2.43231	1.21902	-0.05785
C	1.88170	-0.84212	-1.40127
O	2.45398	-1.22627	2.36186
O	3.27405	2.05216	-0.08879
O	2.44854	-1.45990	-2.24504
C	-1.71191	-0.60756	0.00031
C	-0.44432	-2.58997	0.01969
C	-2.90368	-1.39890	-0.00384
C	-1.66725	0.79429	-0.00692
C	-1.56429	-3.39927	0.01814
H	0.55415	-3.03388	0.02850
C	-2.83690	-2.76467	0.00419
H	-3.87254	-0.90020	-0.01565
C	-2.81416	1.64739	-0.00709
H	-1.46067	-4.48187	0.02580
H	-3.75082	-3.35872	-0.00089
C	-0.29390	2.70106	-0.01557
C	-2.67643	3.00765	-0.00920
H	-3.80767	1.19922	-0.00317
C	-1.36871	3.56766	-0.01254
H	0.72282	3.09983	-0.01986
H	-3.55592	3.65143	-0.00771
H	-1.20409	4.64299	-0.01368
N	-0.38021	1.35583	-0.01362
N	-0.46749	-1.24709	0.00908

[1-CO$_2$]$^-$

Mn	-1.02786	-0.01365	-0.28655
C	-1.33760	-0.05577	-2.04721
C	-2.25516	1.21751	0.03695
C	-2.21651	-1.26115	0.10965
O	-1.66881	-0.08955	-3.16994
O	-3.07509	2.02464	0.24652
O	-3.00919	-2.07921	0.37447
C	1.76569	-0.70844	-0.08866
C	0.49005	-2.63081	-0.32676
C	2.93048	-1.47531	0.02944
C	1.74883	0.74577	-0.09515
C	1.59835	-3.44617	-0.21157
H	-0.50010	-3.05837	-0.47512
C	2.84899	-2.85369	-0.02129
H	3.89511	-0.99120	0.16007
C	2.89543	1.53938	0.02187
H	1.48162	-4.52551	-0.26795
H	3.74566	-3.46244	0.07409
C	0.43140	2.63726	-0.35124

C	2.78383	2.91521	-0.03877
H	3.86971	1.07746	0.16034
C	1.52079	3.47783	-0.23795
H	-0.56734	3.04216	-0.50506
H	3.66619	3.54456	0.05606
H	1.37978	4.55386	-0.30193
N	0.52102	1.29595	-0.27166
N	0.55076	-1.28774	-0.25674
C	-0.78078	0.02274	1.98949
O	-1.82253	0.05206	2.65380
O	0.41068	0.01169	2.31380

[1-CO_2]$^{2-}$

Mn	1.02709	-0.16967	0.28095
C	1.12026	-0.55060	2.03445
C	2.43856	0.88233	0.35147
C	2.06114	-1.46866	-0.29453
O	1.33681	-0.92878	3.12743
O	3.38710	1.56977	0.41707
O	2.76690	-2.32660	-0.68209
C	-1.88925	-0.44226	0.08941
C	-0.86491	-2.54827	0.05183
C	-3.16289	-1.07534	0.00309
C	-1.68199	0.95598	0.14165
C	-2.07441	-3.22070	-0.03619
H	0.07223	-3.10864	0.07168
C	-3.25587	-2.44163	-0.05864
H	-4.06490	-0.46524	-0.01442
C	-2.71589	1.93391	0.08895
H	-2.09814	-4.30666	-0.08426
H	-4.23252	-2.92080	-0.12445
C	-0.09140	2.67372	0.24793
C	-2.41006	3.27017	0.11235
H	-3.75403	1.61192	0.01879
C	-1.05448	3.66935	0.19137
H	0.96504	2.93894	0.30554
H	-3.20465	4.01473	0.06768
H	-0.76244	4.71662	0.20958
N	-0.35610	1.36350	0.23592
N	-0.74193	-1.22069	0.11520
C	0.96263	0.12656	-1.91172
O	1.47129	1.20344	-2.27775
O	0.38785	-0.74610	-2.58467

[1-CO_2H]^{0}

Mn	-1.03142	0.01444	-0.24975
C	-0.97505	0.05216	-2.07139
C	-2.28832	1.26806	-0.15875
C	-2.31372	-1.21623	-0.21657
O	-0.98195	0.07766	-3.22965
O	-3.12198	2.07225	-0.06894
O	-3.16465	-2.00559	-0.16682
C	1.77569	-0.74929	-0.06752
C	0.44232	-2.64048	-0.17038
C	2.92371	-1.53310	0.01942
C	1.79134	0.71790	-0.06005
C	1.54160	-3.48130	-0.08910
H	-0.56223	-3.05054	-0.24764
C	2.80636	-2.91500	0.00985
H	3.90395	-1.07161	0.09939
C	2.95759	1.47692	0.00425
H	1.39682	-4.55818	-0.10192
H	3.69360	-3.54033	0.07806
C	0.49529	2.63703	-0.10752
C	2.86816	2.86091	0.01866
H	3.93066	0.99500	0.04081
C	1.61305	3.45401	-0.03623
H	-0.50183	3.06870	-0.15814
H	3.76964	3.46721	0.06986
H	1.49028	4.53365	-0.02734
N	0.57258	1.30004	-0.11850
N	0.54664	-1.30534	-0.15741
C	-1.11329	-0.02885	1.79920
O	-2.10978	-0.05134	2.50722
O	0.10134	-0.03393	2.43163
H	-0.10208	-0.05635	3.39123

[1-CO_2H]$^{-}$

Mn	-1.02998	0.00290	-0.25868
C	-0.84548	0.00220	-2.06767
C	-2.28492	1.25507	-0.27898
C	-2.29129	-1.24313	-0.28084
O	-0.76561	0.00362	-3.22633
O	-3.12320	2.06467	-0.27071
O	-3.13235	-2.04976	-0.27617
C	1.78866	-0.71469	-0.04920
C	0.46401	-2.65293	-0.03736
C	2.95296	-1.52947	0.00454
C	1.79349	0.70119	-0.05104
C	1.56170	-3.49151	0.02749
H	-0.54215	-3.07404	-0.05935
C	2.84526	-2.89501	0.04276
H	3.93500	-1.05986	0.01804
C	2.96316	1.50825	0.00286
H	1.42579	-4.56938	0.05966
H	3.74108	-3.51329	0.08455
C	0.48179	2.64813	-0.04478
C	2.86437	2.87458	0.03888
H	3.94209	1.03225	0.01912
C	1.58493	3.47958	0.02061
H	-0.52154	3.07586	-0.06897
H	3.76422	3.48693	0.08141

H	1.45623	4.55838	0.05094
N	0.54832	1.31162	-0.08308
N	0.53919	-1.31684	-0.07827
C	-1.22211	0.00631	1.78308
O	-2.25951	-0.00936	2.43710
O	-0.05306	0.02942	2.49974
H	-0.33320	0.02695	3.43988

$[1\text{-}CO_2H]^0$ C-OH Bond Breakage with TFE as proton donor

Mn	1.03986	-0.97234	0.42247
C	1.99609	-0.85196	1.97347
C	1.78019	-2.55050	0.00208
C	-0.25789	-1.88779	1.29502
O	2.62226	-0.77066	2.93717
O	2.23389	-3.57780	-0.27432
O	-0.98233	-2.51903	1.93201
C	1.03154	1.94515	0.33305
C	-0.96568	1.16683	1.23183
C	0.64150	3.26551	0.53297
C	2.28449	1.55819	-0.32879
C	-1.41809	2.46127	1.44976
H	-1.60393	0.30240	1.41316
C	-0.59403	3.52566	1.11044
H	1.28963	4.08435	0.23376
C	3.21209	2.47200	-0.82081
H	-2.40869	2.61742	1.86845
H	-0.91409	4.55225	1.27370
C	3.56856	-0.21940	-1.09111
C	4.34786	2.00509	-1.46683
H	3.04913	3.53982	-0.70379
C	4.52927	0.63547	-1.60932
H	3.67955	-1.29757	-1.18043
H	5.08126	2.70680	-1.85717
H	5.39980	0.22371	-2.11225
N	2.47186	0.22512	-0.46394
N	0.24117	0.91315	0.70862
C	0.13581	-0.89498	-1.31110
O	-1.11746	-2.64191	-1.36784
O	-0.07190	-0.39243	-2.32866
O	-2.50224	-1.07827	-0.15577
H	-1.84528	-1.97233	-0.81526
C	-2.99814	-0.14162	-1.00329
H	-2.36746	0.77633	-1.11336
H	-3.17603	-0.50133	-2.04301
C	-4.33176	0.36742	-0.51783
F	-5.26019	-0.59909	-0.45794
F	-4.82098	1.33070	-1.32133
F	-4.24957	0.89758	0.71615
H	-1.42352	-2.65122	-2.28847

$[1\text{-}CO_2H]^-$ C-OH Bond Breakage with TFE as proton donor

Mn	1.03424	-0.97501	0.43519
C	2.01027	-0.78946	1.95390
C	1.75959	-2.56353	0.05254
C	-0.26364	-1.84310	1.34280
O	2.66250	-0.64275	2.89581
O	2.21058	-3.60401	-0.19273
O	-0.98629	-2.44854	2.01246
C	1.10762	1.93360	0.30189
C	-0.95752	1.21312	1.17970
C	0.72117	3.28152	0.52879
C	2.30637	1.53014	-0.33669
C	-1.38757	2.50835	1.39283
H	-1.62078	0.36378	1.35565
C	-0.50341	3.56628	1.07447
H	1.40229	4.08588	0.25729
C	3.29664	2.41463	-0.84227
H	-2.38509	2.69200	1.78310
H	-0.79762	4.60092	1.24497
C	3.56885	-0.29343	-1.12750
C	4.40090	1.92433	-1.48750
H	3.16685	3.48808	-0.71661
C	4.55019	0.52437	-1.64788
H	3.65088	-1.37660	-1.22160
H	5.15513	2.60683	-1.87611
H	5.40686	0.09198	-2.15776
N	2.47837	0.16092	-0.48898
N	0.25473	0.91039	0.68664
C	0.12560	-0.90038	-1.29708
O	-1.16750	-2.63570	-1.34204
O	-0.08756	-0.40420	-2.31803
O	-2.61991	-1.14717	-0.13061
H	-1.92599	-1.96465	-0.75950
C	-3.05341	-0.18295	-0.98196
H	-2.37970	0.70496	-1.07091
H	-3.22777	-0.52818	-2.02735
C	-4.37072	0.38168	-0.51679
F	-5.34216	-0.54364	-0.47504
F	-4.80516	1.36613	-1.32687
F	-4.28837	0.90607	0.71898
H	-1.47172	-2.61810	-2.26250

$[1\text{-}CO]^+$

Mn	-1.09183	0.00069	0.00065
C	-1.02293	-0.04091	-1.86568
C	-2.37879	1.27378	-0.04647
C	-2.37995	-1.27175	0.02033
O	-0.97497	-0.06519	-3.00836
O	-3.20732	2.07309	-0.08165
O	-3.20897	-2.07126	0.03196

C	1.72780	-0.73455	-0.00297
C	0.40506	-2.64404	0.05686
C	2.88398	-1.50735	-0.03490
C	1.72876	0.73292	-0.00502
C	1.51734	-3.47080	0.03740
H	-0.59551	-3.06683	0.09134
C	2.77758	-2.89074	-0.01473
H	3.86211	-1.03741	-0.07915
C	2.88645	1.50408	0.00828
H	1.38358	-4.54855	0.05855
H	3.67309	-3.50698	-0.03885
C	0.40755	2.64440	-0.00750
C	2.78142	2.88770	0.00856
H	3.86482	1.03279	0.02388
C	1.52104	3.46970	-0.00082
H	-0.59295	3.06872	-0.00845
H	3.67810	3.50264	0.01821
H	1.38819	4.54776	0.00040
N	0.50335	1.30811	-0.01026
N	0.50209	-1.30803	0.03402
C	-1.04933	0.04197	1.86793
O	-1.01369	0.06633	3.01102

[1-CO]^{0}

Mn	-1.11084	0.00001	0.00000
C	-0.99626	-0.00904	-1.85714
C	-2.38525	1.27501	-0.00644
C	-2.38523	-1.27502	0.00619
O	-0.87733	-0.01535	-2.99714
O	-3.21229	2.08184	-0.01100
O	-3.21224	-2.08188	0.01055
C	1.71232	-0.70777	-0.00012
C	0.38559	-2.65751	0.00833
C	2.87639	-1.52101	-0.00614
C	1.71233	0.70775	0.00003
C	1.48634	-3.48515	0.00629
H	-0.61787	-3.08142	0.01291
C	2.76985	-2.88603	-0.00259
H	3.85656	-1.04875	-0.01580
C	2.87641	1.52097	0.00584
H	1.35501	-4.56362	0.01003
H	3.66592	-3.50413	-0.00755
C	0.38563	2.65750	-0.00784
C	2.76990	2.88598	0.00251
H	3.85658	1.04868	0.01518
C	1.48640	3.48513	-0.00593
H	-0.61782	3.08144	-0.01208
H	3.66598	3.50407	0.00730
H	1.35509	4.56360	-0.00945
N	0.46428	1.31440	-0.00470
N	0.46426	-1.31441	0.00494
C	-0.99650	0.00909	1.85718
O	-0.87760	0.01544	2.99717

[2-CH_3CN]$^{+}$

[2-CH_3CN]$^{+}$

Mn	1.37760	0.00043	-0.30770
C	1.37676	0.00104	-2.10246
C	2.65180	-1.27038	-0.26498
C	2.65151	1.27151	-0.26423
O	1.35617	0.00141	-3.25598
O	3.47527	-2.07942	-0.23619
O	3.47476	2.08078	-0.23547
N	1.21499	-0.00051	1.70473
C	1.09916	-0.00184	2.85490
C	0.95619	-0.00379	4.29021
H	0.05356	0.54850	4.57412
H	0.87614	-1.03427	4.65344
H	1.82875	0.47267	4.75051
C	-1.42070	0.71282	-0.17977
C	-0.18218	2.65567	-0.28568
C	-1.42054	-0.71264	-0.18046
C	-2.63610	1.41623	-0.09362
C	-1.34203	3.43862	-0.20536
H	0.79458	3.12841	-0.36592
C	-2.63579	-1.41639	-0.09510
C	-3.85986	0.67978	-0.00094
C	-2.56917	2.82248	-0.10688
H	-1.25121	4.52112	-0.22212
C	-2.56856	-2.82262	-0.10982
C	-3.85971	-0.68031	-0.00167
C	-0.18160	-2.65511	-0.28826
H	-4.79158	1.23903	0.06768
H	-3.48788	3.40360	-0.04245
C	-1.34129	-3.43840	-0.20885
H	-3.48716	-3.40399	-0.04604
H	-4.79132	-1.23984	0.06632
H	0.79529	-3.12753	-0.36883
H	-1.25024	-4.52086	-0.22673
N	-0.21378	1.32854	-0.27094
N	-0.21349	-1.32800	-0.27213

[2-CH_3CN]^{0}

Mn	1.37579	0.00047	-0.32189
C	1.33119	0.00099	-2.09827
C	2.63888	-1.27378	-0.29613
C	2.63863	1.27498	-0.29551
O	1.26796	0.00134	-3.25383
O	3.46154	-2.08981	-0.28939
O	3.46119	2.09111	-0.28864
N	1.24204	-0.00042	1.73468

C	1.13314	-0.00172	2.88599
C	0.99804	-0.00372	4.32354
H	0.09995	0.55266	4.61358
H	0.91440	-1.03348	4.68785
H	1.87486	0.46787	4.78066
C	-1.41866	0.69722	-0.15788
C	-0.17753	2.66907	-0.28592
C	-1.41847	-0.69710	-0.15850
C	-2.63797	1.43289	-0.07940
C	-1.32019	3.44811	-0.20946
H	0.80131	3.14101	-0.37346
C	-2.63760	-1.43313	-0.08075
C	-3.85917	0.67893	0.01421
C	-2.57572	2.81919	-0.10166
H	-1.23048	4.53117	-0.23283
C	-2.57505	-2.81939	-0.10442
C	-3.85899	-0.67954	0.01356
C	-0.17687	-2.66854	-0.28855
H	-4.79662	1.23180	0.08143
H	-3.49339	3.40383	-0.04119
C	-1.31933	-3.44789	-0.21293
H	-3.49257	-3.40430	-0.04452
H	-4.79630	-1.23271	0.08025
H	0.80207	-3.14016	-0.37658
H	-1.22939	-4.53091	-0.23745
N	-0.18976	1.32711	-0.25559
N	-0.18941	-1.32656	-0.25671

[2]^0

Mn	1.48091	0.04043	-0.11211
C	2.04346	-0.17334	1.59621
C	2.66896	1.31644	-0.45635
C	2.63798	-1.12668	-0.80836
O	2.45988	-0.33314	2.67450
O	3.46423	2.14696	-0.66962
O	3.41598	-1.88412	-1.24201
C	-1.31891	-0.71126	-0.07581
C	-0.03990	-2.62805	-0.08756
C	-2.52403	-1.43906	-0.03907
C	-1.34267	0.71129	-0.07527
C	-1.18390	-3.43190	-0.05221
H	0.95135	-3.07761	-0.10329
C	-2.43094	-2.84168	-0.03082
C	-2.57237	1.39631	-0.03817
H	-1.07079	-4.51273	-0.04453
H	-3.33949	-3.44165	-0.00578
C	-0.13116	2.67109	-0.09143
C	-2.52819	2.80172	-0.03010
C	-1.30243	3.43453	-0.05555
H	0.84173	3.15838	-0.11192
H	-3.45684	3.36998	-0.00319
H	-1.22701	4.51867	-0.05018
N	-0.13393	1.33736	-0.10224
N	-0.09117	-1.29616	-0.10354
C	-3.76371	-0.72253	-0.00912
C	-3.78676	0.63733	-0.00852
H	-4.68862	-1.29702	0.01534
H	-4.73067	1.18008	0.01650

[2$_2$]^0

Mn	-0.965895	-1.355775	1.117768
C	-1.902973	-1.515120	2.600477
C	0.517392	-1.924959	1.911594
C	-1.351933	-3.018072	0.619618
O	-2.481098	-1.674305	3.602233
O	1.436588	-2.309868	2.522935
O	-1.637751	-4.112834	0.334075
C	-2.521482	0.863503	0.124541
C	-3.452278	-1.089700	-0.668160
C	-3.526898	1.655904	-0.470534
C	-1.492909	1.470267	0.889510
C	-4.473534	-0.382640	-1.305124
H	-3.420319	-2.175191	-0.727250
C	-4.517811	0.996740	-1.211677
C	-1.468181	2.869087	1.065322
H	-5.226539	-0.933525	-1.863189
H	-5.306958	1.572030	-1.693702
C	0.411017	1.176824	2.153960
C	-0.425832	3.409006	1.835377
C	0.511616	2.552128	2.380781
H	1.145633	0.496225	2.583720
H	-0.368990	4.485405	1.992611
H	1.336572	2.926973	2.982259
N	-0.566048	0.626012	1.425817
N	-2.476954	-0.495976	0.028191
Mn	0.968579	-1.354926	-1.117277
C	1.906713	-1.512508	-2.599486
C	-0.513092	-1.927310	-1.911779
C	1.356829	-3.016341	-0.617926
O	2.485775	-1.670284	-3.600913
O	-1.431071	-2.314504	-2.523501
O	1.643799	-4.110448	-0.331043
C	2.520101	0.868022	-0.125321
C	3.455055	-1.083116	0.667452
C	3.524543	1.662537	0.468478
C	1.489647	1.472422	-0.889562
C	4.475681	-0.373951	1.303069
H	3.424999	-2.168630	0.727150
C	4.517415	1.005463	1.208886
C	1.461460	2.871257	-1.065054
H	5.230238	-0.923209	1.860641
H	5.305951	1.582404	1.689944

C	-0.413849	1.174629	-2.153652
C	0.417296	3.408808	-1.834290
C	-0.518046	2.549756	-2.379906
H	-1.146615	0.492263	-2.583745
H	0.357611	4.485133	-1.991013
H	-1.344124	2.922750	-2.980998
N	0.564622	0.626069	-1.425674
N	2.478012	-0.491462	-0.028251
C	3.472286	3.082799	0.287445
C	2.484703	3.661527	-0.447434
H	4.249015	3.688400	0.753026
H	2.451425	4.741852	-0.584670
C	-2.492613	3.657156	0.446848
C	-3.477845	3.076278	-0.289477
H	-2.461974	4.737527	0.584328
H	-4.255262	3.680192	-0.756108

[2]$^{-}$

Mn	1.47074	0.06994	-0.00847
C	2.24904	-0.63260	1.40867
C	2.64146	1.38956	-0.08494
C	2.34268	-0.79889	-1.27190
O	2.83077	-1.14224	2.29722
O	3.42230	2.26742	-0.13908
O	2.96750	-1.41270	-2.05971
C	-1.31138	-0.68161	-0.01313
C	-0.00321	-2.59271	-0.00991
C	-2.50988	-1.44139	-0.01027
C	-1.33642	0.71645	-0.01227
C	-1.12711	-3.40177	-0.00796
H	0.99692	-3.02601	-0.00729
C	-2.40318	-2.82985	-0.00919
C	-2.56932	1.41815	-0.00764
H	-0.99915	-4.48200	-0.00551
H	-3.30006	-3.44824	-0.00803
C	-0.12066	2.68506	-0.00954
C	-2.53421	2.81018	-0.00442
C	-1.28057	3.43475	-0.00460
H	0.84943	3.17946	-0.01039
H	-3.45820	3.38662	-0.00157
H	-1.20006	4.52005	-0.00197
N	-0.09389	1.32427	-0.01522
N	-0.05793	-1.24284	-0.01558
C	-3.75132	-0.71981	-0.00829
C	-3.77798	0.63963	-0.00679
H	-4.67853	-1.29312	-0.00752
H	-4.72837	1.17393	-0.00479

[2-H]^{0}

Mn	1.50216	-0.00001	-0.19213
C	1.73928	0.00014	1.60632
C	2.72200	1.25008	-0.49203
C	2.72201	-1.25017	-0.49169
O	1.95442	0.00028	2.74701
O	3.52259	2.06088	-0.72710
O	3.52263	-2.06104	-0.72642
C	-1.30799	-0.71222	-0.09409
C	-0.06594	-2.64982	-0.12515
C	-2.52624	-1.41754	-0.05380
C	-1.30799	0.71220	-0.09408
C	-1.22581	-3.43458	-0.08692
H	0.91466	-3.12125	-0.15227
C	-2.45944	-2.82250	-0.05247
C	-2.52625	1.41751	-0.05377
H	-1.13143	-4.51721	-0.08514
H	-3.37902	-3.40519	-0.02302
C	-0.06596	2.64981	-0.12510
C	-2.45946	2.82247	-0.05241
C	-1.22583	3.43456	-0.08684
H	0.91464	3.12124	-0.15222
H	-3.37905	3.40515	-0.02293
H	-1.13146	4.51719	-0.08503
N	-0.09462	1.32104	-0.13181
N	-0.09461	-1.32105	-0.13181
C	-3.75294	-0.68000	-0.01594
C	-3.75294	0.67996	-0.01592
H	-4.68719	-1.23894	0.01291
H	-4.68720	1.23889	0.01295
H	1.30063	-0.00010	-1.80132

[2]$^{2-}$

Mn	1.47958	0.05842	-0.00649
C	2.21736	-0.60752	1.46051
C	2.65638	1.37011	-0.10968
C	2.32663	-0.82239	-1.28690
O	2.79210	-1.10044	2.36898
O	3.44459	2.24609	-0.18421
O	2.94642	-1.44885	-2.07570
C	-1.31225	-0.68659	-0.01366
C	0.00268	-2.61888	-0.00887
C	-2.53062	-1.43820	-0.01244
C	-1.33518	0.72865	-0.01436
C	-1.12863	-3.41812	-0.00882
H	1.00660	-3.04168	-0.00750
C	-2.40614	-2.85781	-0.01209
C	-2.58114	1.43323	-0.01011
H	-1.00270	-4.50126	-0.00705
H	-3.30031	-3.48182	-0.01284
C	-0.09653	2.70519	-0.01461
C	-2.51400	2.85556	-0.00652
C	-1.25789	3.45996	-0.00811

H	0.88355	3.17863	-0.01940
H	-3.43116	3.44510	-0.00283
H	-1.16989	4.54700	-0.00592
N	-0.10354	1.32934	-0.01887
N	-0.07104	-1.24754	-0.01425
C	-3.74254	-0.72492	-0.01182
C	-3.76766	0.67647	-0.00982
H	-4.67842	-1.28761	-0.01166
H	-4.72279	1.20570	-0.00761

$[2\text{-}CO_2]^-$

Mn	-1.33247	-0.04457	-0.29189
C	-1.60778	-0.23332	-2.04936
C	-2.58447	1.19136	-0.13040
C	-2.49153	-1.27472	0.22302
O	-1.92635	-0.38606	-3.16627
O	-3.41857	2.00454	-0.03370
O	-3.26920	-2.08068	0.56456
C	1.46264	-0.68592	-0.11329
C	0.27169	-2.65335	-0.21864
C	2.69396	-1.36757	-0.00387
C	1.42866	0.73213	-0.14507
C	1.43903	-3.40977	-0.10652
H	-0.69697	-3.14354	-0.30732
C	2.66066	-2.76867	0.00599
C	2.62715	1.47319	-0.06424
H	1.36952	-4.49476	-0.10649
H	3.58893	-3.33183	0.09499
C	0.15038	2.63907	-0.33032
C	2.53288	2.87050	-0.11389
C	1.28234	3.44895	-0.25266
H	-0.83779	3.08424	-0.43494
H	3.43478	3.47797	-0.05078
H	1.16304	4.52874	-0.29863
N	0.19911	1.30313	-0.27218
N	0.26437	-1.31891	-0.21569
C	3.89943	-0.59593	0.08890
C	3.86711	0.76232	0.05998
H	4.84521	-1.12956	0.17887
H	4.78646	1.34349	0.12597
C	-1.15541	0.13862	1.98414
O	0.00123	-0.06194	2.36394
O	-2.19290	0.43523	2.58495

$[2\text{-}CO_2]^{2-}$

Mn	-1.32292	-0.00144	-0.28445
C	-1.43471	-0.01865	-2.08507
C	-2.54395	1.25257	-0.10712
C	-2.54749	-1.24542	-0.06905
O	-1.76888	-0.04398	-3.21296
O	-3.35968	2.08867	0.00482
O	-3.37408	-2.06713	0.07162
C	1.49623	-0.68976	-0.09513
C	0.26386	-2.65946	-0.13267
C	2.72137	-1.42696	-0.02106
C	1.49192	0.70341	-0.09040
C	1.41035	-3.44798	-0.05763
H	-0.71881	-3.13424	-0.17488
C	2.66398	-2.81315	-0.00370
C	2.71446	1.44181	-0.01666
H	1.31901	-4.53154	-0.04135
H	3.58689	-3.39173	0.05383
C	0.25453	2.67361	-0.11984
C	2.65740	2.82747	0.00285
C	1.39453	3.46022	-0.04887
H	-0.72980	3.14502	-0.15941
H	3.57861	3.40841	0.05922
H	1.30119	4.54386	-0.03243
N	0.25870	1.33450	-0.14317
N	0.26661	-1.32752	-0.15543
C	3.94292	-0.67237	0.03881
C	3.93964	0.68633	0.03991
H	4.88397	-1.22272	0.08848
H	4.87864	1.24017	0.09028
C	-1.42185	-0.01282	1.92983
O	-0.29594	-0.08053	2.45277
O	-2.54179	0.05008	2.47530

$[2\text{-}CO_2H]^0$

Mn	-1.34064	-0.00013	-0.24896
C	-1.26473	-0.00036	-2.07085
C	-2.60241	1.25070	-0.20385
C	-2.60214	-1.25121	-0.20333
O	-1.26217	-0.00049	-3.22936
O	-3.43801	2.05551	-0.14688
O	-3.43750	-2.05625	-0.14597
C	1.45976	-0.71237	-0.07767
C	0.21765	-2.65019	-0.11143
C	2.67655	-1.41700	-0.01377
C	1.45966	0.71254	-0.07774
C	1.37667	-3.43535	-0.04810
H	-0.76273	-3.12128	-0.15026
C	2.60915	-2.82227	-0.00090
C	2.67635	1.41735	-0.01388
H	1.28218	-4.51790	-0.03738
H	3.52826	-3.40455	0.04715
C	0.21727	2.65019	-0.11167
C	2.60875	2.82260	-0.00110
C	1.37618	3.43552	-0.04836
H	-0.76318	3.12113	-0.15055
H	3.52777	3.40502	0.04693
H	1.28155	4.51805	-0.03771

N	0.24821	1.32266	-0.12693
N	0.24840	-1.32266	-0.12676
C	3.90304	-0.67965	0.04019
C	3.90294	0.68017	0.04015
H	4.83667	-1.23866	0.08422
H	4.83649	1.23932	0.08414
C	-1.44867	0.00031	1.79804
O	-0.24177	-0.00057	2.44520
O	-2.45384	0.00133	2.49408
H	-0.45714	-0.00013	3.40254

$[2\text{-}CO_2H]^-$

Mn	-1.34337	-0.00003	-0.25471
C	-1.17360	-0.00025	-2.06566
C	-2.59196	1.25787	-0.26520
C	-2.59203	-1.25788	-0.26480
O	-1.10366	-0.00034	-3.22482
O	-3.42126	2.07617	-0.25117
O	-3.42131	-2.07618	-0.25051
C	1.46364	-0.69679	-0.05592
C	0.22030	-2.66650	-0.06224
C	2.68580	-1.43426	-0.00152
C	1.46366	0.69668	-0.05601
C	1.36424	-3.45129	-0.00451
H	-0.76359	-3.13780	-0.08785
C	2.62383	-2.81997	0.02215
C	2.68582	1.43416	-0.00166
H	1.27150	-4.53437	0.01682
H	3.54435	-3.40264	0.06352
C	0.22033	2.66638	-0.06253
C	2.62385	2.81988	0.02190
C	1.36429	3.45119	-0.00481
H	-0.76355	3.13769	-0.08819
H	3.54437	3.40254	0.06324
H	1.27155	4.53427	0.01645
N	0.23207	1.32954	-0.09064
N	0.23204	-1.32963	-0.09045
C	3.91018	-0.67928	0.03307
C	3.91019	0.67919	0.03301
H	4.85028	-1.23164	0.06514
H	4.85030	1.23154	0.06504
C	-1.52205	0.00026	1.78866
O	-0.34747	0.00110	2.49656
O	-2.55486	-0.00019	2.44977
H	-0.62028	0.00094	3.43889

$[2\text{-}CO_2H]^0$ C-OH Bond Breakage with TFE as proton donor

Mn	-1.91308	-0.28818	0.13275
C	-2.81526	0.68995	1.38974
C	-2.77674	-1.76697	0.66530
C	-3.17547	0.02790	-1.11554
O	-3.38521	1.29561	2.18522
O	-3.34522	-2.71317	1.01044
O	-3.99409	0.22450	-1.90423
C	0.32182	1.51697	0.40943
C	-1.10005	2.36333	-1.19039
C	1.21441	2.59547	0.28185
C	0.59959	0.47220	1.33541
C	-0.26928	3.47404	-1.38866
H	-2.01800	2.25623	-1.76333
C	0.88832	3.59399	-0.65248
C	1.77046	0.51061	2.11483
H	-0.55645	4.22490	-2.11976
H	1.55309	4.44701	-0.78212
C	-0.06974	-1.53669	2.25253
C	1.99765	-0.56724	2.98984
C	1.07779	-1.59057	3.05410
H	-0.80920	-2.33441	2.29578
H	2.89604	-0.57893	3.60541
H	1.22054	-2.44015	3.71626
N	-0.30950	-0.53184	1.41762
N	-0.81748	1.40765	-0.31470
C	2.39443	2.62162	1.09167
C	2.66231	1.62124	1.97317
H	3.07450	3.46508	0.98103
H	3.56272	1.63903	2.58549
C	-0.92079	-1.42734	-1.11102
O	-0.83935	-0.22920	-2.95857
O	-0.46748	-2.42992	-1.45464
O	1.29619	0.18071	-1.91445
H	0.19485	0.01247	-2.53145
C	2.18112	-0.77147	-2.29556
H	1.72421	-1.66992	-2.77441
H	2.96778	-0.42721	-3.00878
C	2.95234	-1.30958	-1.11524
F	3.64438	-0.34825	-0.48200
F	3.84500	-2.24603	-1.49345
F	2.16335	-1.88431	-0.19070
H	-0.68496	-0.94622	-3.59221

$[2\text{-}CO]^+$

Mn	1.41502	0.00002	-0.00004
C	1.35793	0.00139	-1.86755
C	2.69499	-1.28052	-0.00147
C	2.69501	1.28054	0.00125
O	1.31489	0.00223	-3.01061
O	3.51805	-2.08592	-0.00262
O	3.51812	2.08589	0.00235
C	-1.38771	0.71169	0.00015
C	-0.14164	2.65594	0.00059
C	-2.60509	1.41646	0.00004

C	-1.38771	-0.71168	-0.00012
C	-1.30423	3.43751	0.00039
H	0.83715	3.12965	0.00080
C	-2.53584	2.82239	0.00013
C	-2.60508	-1.41646	-0.00010
H	-1.21105	4.51979	0.00044
H	-3.45597	3.40454	-0.00002
C	-0.14163	-2.65593	-0.00015
C	-2.53582	-2.82240	-0.00002
C	-1.30421	-3.43750	0.00001
H	0.83717	-3.12962	-0.00013
H	-3.45594	-3.40455	0.00005
H	-1.21102	-4.51978	0.00015
N	-0.17646	-1.32793	-0.00028
N	-0.17646	1.32794	0.00049
C	-3.83225	0.68007	-0.00014
C	-3.83224	-0.68009	-0.00014
H	-4.76601	1.23977	-0.00022
H	-4.76600	-1.23980	-0.00019
C	1.35817	-0.00138	1.86741
O	1.31526	-0.00227	3.01048

$[2\text{-CO}]^0$

Mn	1.43319	0.00003	-0.00006
C	1.31832	0.00008	-1.85813
C	2.69892	-1.28275	0.00020
C	2.69902	1.28273	-0.00020
O	1.19773	0.00026	-2.99786
O	3.51800	-2.09732	0.00061
O	3.51826	2.09714	-0.00039
C	-1.37291	0.69617	0.00005
C	-0.12292	2.67326	0.00015
C	-2.59396	1.43373	0.00014
C	-1.37287	-0.69621	-0.00003
C	-1.26864	3.44940	0.00024
H	0.85911	3.14524	0.00021
C	-2.52896	2.81919	0.00021
C	-2.59389	-1.43377	-0.00001
H	-1.17796	4.53244	0.00034
H	-3.44811	3.40429	0.00027
C	-0.12283	-2.67339	-0.00020
C	-2.52892	-2.81924	-0.00006
C	-1.26849	-3.44946	-0.00015
H	0.85921	-3.14535	-0.00029
H	-3.44806	-3.40435	-0.00003
H	-1.17783	-4.53250	-0.00020
N	-0.14128	-1.33010	-0.00012
N	-0.14136	1.33012	-0.00000
C	-3.81847	0.67913	0.00015
C	-3.81843	-0.67915	0.00008
H	-4.75796	1.23224	0.00021
H	-4.75791	-1.23229	0.00010
C	1.31848	0.00002	1.85787
O	1.19818	0.00001	2.99763

$[4\text{-CH}_3\text{CN}]^+$

$[4\text{-CH}_3\text{CN}]^+$

Mn	0.00318	-0.93352	-0.64779
C	1.23039	-2.10371	-1.24260
C	-1.20806	-2.08913	-1.29841
C	0.03604	-0.13049	-2.25531
O	1.96606	-2.87913	-1.68136
O	0.05606	0.39875	-3.27985
O	-1.93040	-2.85340	-1.77754
C	-0.73222	1.77058	0.03590
C	-2.66505	0.47519	0.23098
C	-1.47149	2.96728	0.11563
C	0.74313	1.76688	0.02869
C	-3.47301	1.64918	0.28969
C	-2.83626	2.90177	0.20293
H	-0.97236	3.93047	0.10446
C	1.49381	2.95886	0.04394
H	-3.43788	3.80889	0.23779
C	2.66550	0.46387	0.26556
C	2.85847	2.88474	0.13072
H	1.00385	3.92424	-0.02808
C	3.48378	1.63190	0.27847
H	3.46865	3.78673	0.11678
C	4.63501	-0.87165	0.70511
H	5.08596	-1.84106	0.90851
C	5.45133	0.27933	0.65877
H	6.52553	0.18619	0.80353
C	-4.87246	1.53420	0.46005
H	-5.46580	2.44744	0.48400
C	-5.45530	0.30217	0.60905
H	-6.53092	0.21366	0.74577
C	-4.64970	-0.85703	0.61239
H	-5.11000	-1.82975	0.77499
C	-3.29078	-0.77824	0.42745
H	-2.68573	-1.67755	0.48448
C	4.88153	1.51017	0.45770
H	5.48424	2.41751	0.44700
C	3.27760	-0.78743	0.51214
H	2.66186	-1.67724	0.60190
C	-0.06001	-2.10402	2.29653
C	-0.09268	-2.61737	3.64422
H	-0.98755	-3.23383	3.78424
H	0.79742	-3.22859	3.83004
H	-0.11324	-1.78600	4.35750
N	-0.03508	-1.69370	1.21611
N	1.31413	0.55913	0.06510

N	-1.31258	0.56670	0.03224

[4-CH_3CN]^0

Mn	-0.00028	-0.90682	-0.67153
C	1.22230	-2.04724	-1.32344
C	-1.22335	-2.04770	-1.32172
C	-0.00132	-0.05371	-2.23764
O	1.94522	-2.80292	-1.82328
O	-0.00195	0.52102	-3.24107
O	-1.94677	-2.80371	-1.82032
C	-0.71425	1.77719	0.05983
C	-2.66132	0.45064	0.26679
C	-1.49438	2.96415	0.09836
C	0.71348	1.77741	0.05991
C	-3.48369	1.61593	0.31589
C	-2.85028	2.88875	0.19032
H	-1.00668	3.93448	0.05130
C	1.49312	2.96464	0.10037
H	-3.46414	3.78891	0.20592
C	2.66107	0.45136	0.26538
C	2.84903	2.88966	0.19228
H	1.00499	3.93485	0.05542
C	3.48298	1.61692	0.31579
H	3.46250	3.79006	0.20949
C	4.64953	-0.89394	0.67011
H	5.09560	-1.87359	0.83686
C	5.45853	0.25025	0.66799
H	6.53363	0.16422	0.81440
C	-4.87008	1.48889	0.50286
H	-5.47191	2.39831	0.52267
C	-5.45852	0.24880	0.67041
H	-6.53353	0.16249	0.81723
C	-4.64902	-0.89504	0.67380
H	-5.09464	-1.87465	0.84198
C	-3.28239	-0.79916	0.48229
H	-2.66771	-1.69365	0.53786
C	4.86948	1.49027	0.50226
H	5.47089	2.39995	0.52314
C	3.28280	-0.79843	0.47911
H	2.66861	-1.69328	0.53378
C	0.00286	-2.16017	2.26333
C	0.00555	-2.69406	3.60483
H	-0.92998	-3.23224	3.79351
H	0.84701	-3.38467	3.72867
H	0.10172	-1.87805	4.32950
N	0.00117	-1.73204	1.18916
N	1.30820	0.54481	0.05264
N	-1.30852	0.54435	0.05380

[4]^0

Mn	-0.00118	-1.10044	-0.28107
C	-1.20798	-2.07336	-1.15477
C	1.18672	-2.06551	-1.18934
C	0.01846	-2.15940	1.18861
O	-1.97090	-2.72914	-1.74863
O	0.02999	-2.89890	2.09145
O	1.93737	-2.71542	-1.80490
C	0.73282	1.65182	-0.22245
C	2.66208	0.41033	0.23359
C	1.48669	2.83318	-0.39753
C	-0.73300	1.64922	-0.23213
C	3.47592	1.56581	0.06458
C	2.84800	2.78200	-0.28379
H	0.99388	3.77364	-0.62352
C	-1.49121	2.82056	-0.45080
H	3.45664	3.67313	-0.43145
C	-2.66002	0.41179	0.24226
C	-2.85311	2.76647	-0.34383
H	-1.00128	3.75323	-0.71276
C	-3.47743	1.55959	0.04212
H	-3.46496	3.64899	-0.52589
C	-4.62666	-0.83328	0.90364
H	-5.07693	-1.75568	1.26594
C	-5.44208	0.29268	0.66481
H	-6.51603	0.22770	0.82768
C	4.87234	1.47549	0.26422
H	5.47434	2.37160	0.11611
C	5.44608	0.28921	0.64901
H	6.52059	0.22322	0.80761
C	4.63453	-0.84538	0.85794
H	5.08841	-1.77631	1.19289
C	3.27590	-0.79134	0.65291
H	2.65393	-1.66419	0.83761
C	-4.87334	1.46975	0.24555
H	-5.47896	2.35907	0.07326
C	-3.26866	-0.78053	0.69431
H	-2.64240	-1.64528	0.90199
N	-1.30100	0.46415	0.03474
N	1.30303	0.46370	0.02531

[4_2]^0

Mn	-0.452822	-0.959736	1.265940
C	-0.608212	-1.639611	2.880005
C	-2.047696	-0.168887	1.352805
C	0.047862	0.605101	1.923151
O	-0.732800	-2.030334	3.971678
O	-3.043550	0.410037	1.542750
O	0.257008	1.614980	2.473730
C	1.399137	-2.707557	-0.088084
C	2.645745	-1.252819	1.250993
C	2.565261	-3.143536	-0.761056
C	0.094243	-3.302695	-0.334933
C	3.849573	-1.643739	0.598596

C	3.769021	-2.573588	-0.464961
C	-0.054457	-4.440944	-1.163706
H	4.676763	-2.859870	-0.995397
C	-2.179864	-3.329122	0.208670
C	-1.291730	-4.974142	-1.360561
C	-2.396833	-4.438686	-0.657252
H	-1.441252	-5.828429	-2.019504
N	-0.941003	-2.714201	0.287053
N	1.413073	-1.705367	0.807467
Mn	-0.510303	0.937830	-1.262905
C	-0.706988	1.611224	-2.875293
C	-2.067724	0.074325	-1.340031
C	0.057748	-0.601301	-1.925235
O	-0.855904	1.997003	-3.965671
O	-3.036815	-0.550084	-1.524052
O	0.310975	-1.599673	-2.478264
C	1.268127	2.768288	0.079294
C	2.571104	1.362911	-1.258001
C	2.416552	3.255006	0.748009
C	-0.059596	3.308257	0.328925
C	3.758871	1.803495	-0.607895
C	3.642058	2.733517	0.452437
C	-0.251409	4.446819	1.148299
H	4.538043	3.057255	0.981314
C	-2.335688	3.232453	-0.200709
C	-1.508922	4.929505	1.347108
C	-2.594283	4.340222	0.656312
H	-1.691192	5.783579	1.998086
N	-1.071906	2.671165	-0.283016
N	1.321321	1.764609	-0.812882
H	2.317363	4.009844	1.522156
H	0.598204	4.922460	1.627704
H	2.496449	-3.899164	-1.537700
H	0.810917	-4.875584	-1.653547
C	2.713332	0.561101	-2.411047
C	5.018816	1.347562	-1.054532
C	5.121876	0.520189	-2.147078
C	3.955511	0.146516	-2.839963
C	-3.404456	2.755933	-0.991608
C	-3.901649	4.862358	0.765907
C	-4.665888	3.297942	-0.884747
H	-5.464820	2.913756	-1.516677
C	-4.930638	4.344772	0.017342
H	-5.935521	4.754179	0.099850
C	-3.261853	-2.906681	1.012281
C	-3.681557	-5.015146	-0.762909
C	-4.726076	-4.549425	-0.001771
H	-5.713111	-5.000657	-0.081466
C	-4.499661	-3.501304	0.909208
H	-5.309479	-3.158125	1.550784
C	2.756919	-0.446029	2.404171
C	5.090977	-1.139120	1.045044
C	5.162100	-0.310054	2.138702
C	3.982092	0.017293	2.832129
H	4.035335	0.624807	3.734044
H	5.906327	1.687002	-0.519927
H	1.838433	0.314010	-3.000986
H	4.032188	-0.458494	-3.741878
H	6.095999	0.177534	-2.491199
H	5.990587	-1.441949	0.508649
H	6.122068	0.070192	2.482972
H	1.873134	-0.232884	2.994303
H	-3.107962	-2.127940	1.749911
H	-3.814132	-5.851280	-1.449295
H	-3.223431	1.977121	-1.722944
H	-4.064933	5.698975	1.445038

[4]⁻

Mn	-0.00012	-1.07544	0.22061
C	0.00006	-2.09907	-1.20657
C	1.19069	-2.05119	1.09392
C	-1.19099	-2.05138	1.09358
O	-0.00004	-2.83260	-2.12226
O	-1.94159	-2.70341	1.71286
O	1.94116	-2.70302	1.71364
C	0.70390	1.69507	0.12686
C	2.65423	0.38828	-0.18504
C	1.49818	2.86577	0.28491
C	-0.70376	1.69504	0.12699
C	3.47797	1.53429	-0.00846
C	2.85337	2.79013	0.27408
H	1.00795	3.82536	0.43316
C	-1.49804	2.86567	0.28576
H	3.47553	3.67242	0.42110
C	-2.65416	0.38836	-0.18510
C	-2.85322	2.79004	0.27511
H	-1.00774	3.82512	0.43465
C	-3.47783	1.53431	-0.00805
H	-3.47541	3.67220	0.42275
C	-4.64767	-0.88501	-0.76594
H	-5.09045	-1.82195	-1.10143
C	-5.46172	0.22587	-0.51623
H	-6.54196	0.15597	-0.63312
C	4.87158	1.42312	-0.15751
H	5.47783	2.31608	0.00139
C	5.46183	0.22556	-0.51598
H	6.54208	0.15556	-0.63274
C	4.64774	-0.88539	-0.76530
H	5.09050	-1.82246	-1.10043
C	3.27403	-0.80124	-0.61079
H	2.64975	-1.66061	-0.84390
C	-4.87144	1.42326	-0.15722

H	-5.47766	2.31617	0.00202
C	-3.27396	-0.80098	-0.61130
H	-2.64978	-1.66035	-0.84478
N	-1.27309	0.44296	0.02038
N	1.27326	0.44298	0.02046

[4-H]^{0}

Mn	-0.00124	-1.13258	-0.35264
C	0.01717	-1.81064	1.33165
C	-1.21761	-2.24949	-0.98982
C	1.19919	-2.24601	-1.02449
O	0.02826	-2.33243	2.36985
O	1.94437	-2.97225	-1.54535
O	-1.97505	-2.97988	-1.48655
C	-0.73495	1.65037	-0.25061
C	-2.66206	0.41427	0.19013
C	-1.48534	2.82975	-0.44865
C	0.73470	1.65251	-0.24094
C	-3.47767	1.56750	-0.00231
C	-2.84880	2.77742	-0.36006
H	-0.99083	3.76653	-0.68475
C	1.48069	2.84196	-0.38978
H	-3.45652	3.66473	-0.53245
C	2.66435	0.41218	0.17885
C	2.84352	2.79303	-0.29378
H	0.98319	3.78634	-0.58549
C	3.47608	1.57363	0.02232
H	3.44794	3.68920	-0.42754
C	4.64886	-0.83665	0.78980
H	5.10708	-1.76695	1.12042
C	5.45628	0.30178	0.58147
H	6.53193	0.23824	0.73267
C	-4.87629	1.48155	0.18887
H	-5.47598	2.37524	0.01895
C	-5.45243	0.30527	0.59603
H	-6.52772	0.24231	0.75008
C	-4.64084	-0.82353	0.83873
H	-5.09563	-1.74456	1.19869
C	-3.28255	-0.77633	0.63776
H	-2.66942	-1.64500	0.85980
C	4.87496	1.48766	0.21141
H	5.47074	2.38880	0.06939
C	3.29019	-0.78832	0.59122
H	2.68164	-1.66629	0.78658
N	1.30804	0.46530	-0.02517
N	-1.30584	0.46660	-0.01154
H	-0.01622	-0.60826	-1.88388

[4]$^{2-}$

Mn	0.00001	-1.11689	0.24897
C	1.19436	-2.07439	1.14310
C	-1.19432	-2.07411	1.14346
C	-0.00019	-2.20765	-1.20765
O	1.94480	-2.74193	1.76122
O	-0.00028	-3.07212	-2.00845
O	-1.94476	-2.74146	1.76178
C	-0.69600	1.70808	0.17816
C	-2.66082	0.42197	-0.23283
C	-1.49913	2.86933	0.38769
C	0.69605	1.70807	0.17821
C	-3.49375	1.57426	-0.05614
C	-2.85265	2.81262	0.30662
H	-1.01332	3.81753	0.61556
C	1.49919	2.86931	0.38777
H	-3.46856	3.69755	0.47224
C	2.66086	0.42195	-0.23278
C	2.85270	2.81259	0.30675
H	1.01338	3.81752	0.61565
C	3.49380	1.57423	-0.05605
H	3.46862	3.69751	0.47239
C	4.68013	-0.83244	-0.83166
H	5.12659	-1.77322	-1.15624
C	5.48672	0.28401	-0.61487
H	6.56583	0.23061	-0.75277
C	-4.87616	1.47984	-0.23936
H	-5.47644	2.37898	-0.08229
C	-5.48669	0.28402	-0.61485
H	-6.56580	0.23062	-0.75272
C	-4.68011	-0.83245	-0.83159
H	-5.12659	-1.77325	-1.15609
C	-3.30176	-0.76557	-0.65427
H	-2.68340	-1.64012	-0.84967
C	4.87621	1.47981	-0.23930
H	5.47650	2.37894	-0.08221
C	3.30178	-0.76556	-0.65431
H	2.68341	-1.64008	-0.84977
N	1.30587	0.46115	-0.01649
N	-1.30583	0.46117	-0.01659

[4-CO$_2$]$^{2-}$

Mn	-0.00061	-1.09682	0.03218
C	0.07579	-1.83947	-1.60624
C	1.15637	-2.20344	0.75411
C	-1.18669	-2.25033	0.62828
O	0.14598	-2.53138	-2.55540
O	-1.91003	-3.07048	1.05395
O	1.86820	-2.98560	1.26378
C	0.71639	1.71892	-0.05861
C	2.66826	0.45826	-0.43607
C	1.49255	2.89381	0.14203
C	-0.70709	1.71881	-0.05875
C	3.49421	1.61031	-0.24790

C	2.85096	2.84270	0.08031
H	0.99780	3.84002	0.34988
C	-1.48573	2.88350	0.18586
H	3.45904	3.73260	0.24453
C	-2.65681	0.46559	-0.46959
C	-2.84480	2.82871	0.13461
H	-0.99301	3.82160	0.43189
C	-3.48569	1.60726	-0.23703
H	-3.45499	3.70858	0.33991
C	-4.66738	-0.78160	-1.07207
H	-5.11988	-1.70870	-1.42375
C	-5.47664	0.32905	-0.79722
H	-6.55734	0.26836	-0.91599
C	4.88626	1.51391	-0.40370
H	5.48503	2.41214	-0.24255
C	5.48943	0.31955	-0.76198
H	6.56994	0.25885	-0.88224
C	4.68428	-0.80528	-0.98574
H	5.14013	-1.74549	-1.29593
C	3.31107	-0.74077	-0.82814
H	2.69572	-1.61477	-1.02563
C	-4.87782	1.51077	-0.39212
H	-5.48028	2.39898	-0.19345
C	-3.29420	-0.71756	-0.91414
H	-2.67398	-1.57723	-1.15546
N	-1.30133	0.50982	-0.29838
N	1.31178	0.50642	-0.27012
C	-0.06653	-0.36757	2.13769
O	1.03505	-0.10379	2.65264
O	-1.21211	-0.24112	2.60783

[4-CO_2H]^0

Mn	-0.00592	-1.10091	0.07499
C	0.00188	-1.67994	-1.65543
C	1.19018	-2.29450	0.61792
C	-1.21522	-2.28274	0.61438
O	0.01256	-2.10689	-2.73300
O	-1.95843	-3.07182	1.03236
O	1.91844	-3.10496	1.02193
C	0.72557	1.69087	-0.04359
C	2.65742	0.45125	-0.44669
C	1.47082	2.87456	0.14231
C	-0.74651	1.68706	-0.03288
C	3.47092	1.60434	-0.24325
C	2.83599	2.81972	0.08172
H	0.97281	3.81749	0.34353
C	-1.49668	2.85938	0.19969
H	3.44086	3.70968	0.25052
C	-2.67475	0.44505	-0.44092
C	-2.86214	2.79708	0.15216
H	-1.00232	3.79650	0.43441
C	-3.49324	1.58791	-0.20248
H	-3.47072	3.67699	0.35668
C	-4.66305	-0.79593	-1.05351
H	-5.12138	-1.71304	-1.41889
C	-5.47588	0.32062	-0.76197
H	-6.55493	0.25111	-0.88323
C	4.87384	1.51484	-0.39885
H	5.47051	2.40887	-0.22050
C	5.45777	0.33498	-0.78329
H	6.53650	0.26847	-0.90888
C	4.64944	-0.79261	-1.04220
H	5.11045	-1.71611	-1.38757
C	3.28668	-0.74126	-0.87623
H	2.67874	-1.60816	-1.11559
C	-4.89667	1.49379	-0.35071
H	-5.49764	2.37920	-0.14573
C	-3.29973	-0.74111	-0.89441
H	-2.68767	-1.59831	-1.15790
N	-1.31580	0.50589	-0.27903
N	1.29945	0.50709	-0.26926
C	-0.05114	-0.43230	2.01180
O	1.17621	-0.28685	2.60284
O	-1.03498	-0.13267	2.66954
H	1.00389	0.05862	3.50546

[4-CO_2H]$^-$

Mn	-0.00671	-1.09953	0.09676
C	-0.02666	-1.67081	-1.62841
C	1.20653	-2.28226	0.61384
C	-1.20286	-2.28181	0.65304
O	-0.03274	-2.08951	-2.71259
O	-1.92976	-3.08434	1.08371
O	1.94211	-3.09699	1.00580
C	0.69672	1.69386	-0.01936
C	2.64582	0.44820	-0.48177
C	1.47525	2.85795	0.22613
C	-0.73038	1.69281	-0.01759
C	3.47131	1.59126	-0.25197
C	2.83281	2.80466	0.14630
H	0.98494	3.79212	0.48936
C	-1.50797	2.86301	0.20124
H	3.44446	3.68375	0.34903
C	-2.68068	0.43746	-0.44623
C	-2.86546	2.80858	0.12490
H	-1.01649	3.80487	0.43275
C	-3.50561	1.58530	-0.23762
H	-3.47609	3.69354	0.30368
C	-4.68648	-0.80761	-1.05546
H	-5.13885	-1.73707	-1.39974
C	-5.49376	0.31034	-0.80513
H	-6.57274	0.25191	-0.93707

C	4.86074	1.50472	-0.43674
H	5.46189	2.39407	-0.24159
C	5.45527	0.33129	-0.86647
H	6.53310	0.27705	-1.00925
C	4.64686	-0.78133	-1.13576
H	5.09682	-1.70200	-1.50585
C	3.27716	-0.72721	-0.95035
H	2.66416	-1.59043	-1.19648
C	-4.89647	1.49392	-0.40908
H	-5.49632	2.38755	-0.23031
C	-3.31538	-0.74853	-0.88343
H	-2.70455	-1.61702	-1.11495
N	-1.32592	0.48807	-0.26016
N	1.29280	0.49311	-0.28344
C	-0.01773	-0.41687	2.03332
O	1.22236	-0.24348	2.60168
O	-0.98516	-0.15416	2.73355
H	1.05068	0.08799	3.50899
H	-4.07175	-3.62951	0.73978
C	-2.50780	-2.17847	0.74594
H	-1.78963	-2.83733	0.26461
C	5.14519	1.39797	0.97306
H	5.62674	2.30309	1.34306
C	3.86151	-0.91214	0.06204
H	3.36595	-1.82415	-0.26147
N	1.69947	0.06050	0.56100
N	-0.82327	-0.44032	0.83114
O	0.02658	2.01647	-1.31659
H	0.22405	0.24519	-1.26841
C	-0.92230	2.15642	-2.24187
H	-0.84410	3.08223	-2.87487
H	-1.01165	1.31604	-2.99458
C	-2.32132	2.23739	-1.66067
F	-3.25367	2.49633	-2.60518
F	-2.44230	3.20027	-0.72971
F	-2.70157	1.08859	-1.06713

[4-CO_2H]^0 C-OH Bond Breakage with TFE as proton donor

Mn	0.50906	-1.20956	-0.54732
C	0.90269	-2.84427	0.11243
C	-0.73424	-1.73561	-1.68076
C	1.60476	-1.23269	-1.92346
O	1.16566	-3.95621	0.36496
O	2.28917	-1.18852	-2.87170
O	-1.53657	-2.04045	-2.47588
C	-0.42580	0.73640	1.32769
C	-2.09758	-0.87675	1.11334
C	-1.29920	1.60367	2.02605
C	0.99679	1.02044	1.17166
C	-3.02321	-0.05185	1.81267
C	-2.59271	1.22587	2.23973
H	-0.94483	2.56688	2.38096
C	1.59807	2.19296	1.68649
H	-3.29346	1.87990	2.75767
C	3.07199	0.16010	0.53784
C	2.94858	2.35629	1.58331
H	0.98728	2.96541	2.14425
C	3.73520	1.31970	1.02832
H	3.43728	3.25911	1.94803
C	5.23346	-0.81737	0.03855
H	5.82212	-1.66013	-0.32021
C	5.88608	0.35133	0.48100
H	6.97211	0.41349	0.44571
C	-4.33050	-0.52293	2.06701
H	-5.02345	0.13576	2.59067
C	-4.71102	-1.78310	1.67389
H	-5.71834	-2.14276	1.87529
C	-3.78178	-2.61876	1.02230

[4-CO_2H]$^-$ C-OH Bond Breakage with TFE as proton donor

Mn	-0.735971	0.294643	-1.089895
C	-1.408271	1.918080	-1.571213
C	0.480608	0.311829	-2.430444
C	-1.717849	-0.630148	-2.265387
O	-1.855344	2.950351	-1.826055
O	-2.272884	-1.255596	-3.068325
O	1.106484	0.348365	-3.401179
C	-0.093693	0.860679	1.665003
C	1.553245	1.913051	0.334425
C	0.701557	1.045539	2.829354
C	-1.421989	0.344074	1.709545
C	2.379901	2.127567	1.478963
C	1.923800	1.634865	2.737692
H	0.329273	0.714857	3.795433
C	-2.058182	-0.061574	2.914056
H	2.551046	1.770697	3.618314
C	-3.419274	-0.024965	0.493443
C	-3.354342	-0.473747	2.898919
H	-1.503485	-0.048508	3.848629
C	-4.099017	-0.431559	1.681202
H	-3.851095	-0.803312	3.811048
C	-5.534510	-0.209701	-0.700669
H	-6.094907	-0.092294	-1.627271
C	-6.183680	-0.657538	0.456441
H	-7.242781	-0.907125	0.432583
C	3.601467	2.808240	1.347827
H	4.213591	2.941463	2.240692
C	4.016799	3.303730	0.125573
H	4.965875	3.828638	0.034452
C	3.185208	3.142919	-0.990670

H	3.480996	3.559472	-1.952625
C	1.981111	2.470277	-0.890109
H	1.341213	2.391562	-1.763863
C	-5.464893	-0.753814	1.634216
H	-5.949487	-1.072637	2.557918
C	-4.186446	0.102373	-0.685141
H	-3.720431	0.488154	-1.587748
N	-2.076167	0.262849	0.510136
N	0.378330	1.208927	0.428693
C	-0.201371	-1.434770	-0.334885
O	1.029975	-2.380981	-1.953803
O	-0.244474	-2.311748	0.410744
O	3.081121	-1.182144	-1.396561
H	1.987507	-1.850911	-1.725183
C	3.157880	-1.095846	-0.054816
H	3.890066	-0.336075	0.308660
H	2.201003	-0.843021	0.473678
C	3.601366	-2.392158	0.588102
F	3.666459	-2.294309	1.931794
F	4.814678	-2.788277	0.170031
F	2.757547	-3.408093	0.319565
H	1.142022	-3.243753	-1.528786

$[4\text{-CO}]^{+}$

Mn	-0.00032	-1.10743	0.26146
C	-0.05586	-1.54017	-1.55948
C	1.23940	-2.37110	0.63652
C	-1.22764	-2.35467	0.71929
O	-0.09498	-1.78315	-2.67669
O	-1.95575	-3.17962	1.06049
O	1.97512	-3.21116	0.91910
C	0.73682	1.66527	0.12779
C	2.66693	0.42922	-0.33940
C	1.48532	2.83685	0.34864
C	-0.73661	1.66937	0.12108
C	3.48056	1.57952	-0.11270
C	2.85091	2.77948	0.26735
H	0.99251	3.77003	0.59991
C	-1.47686	2.85755	0.27119
H	3.45889	3.66122	0.46425
C	-2.67372	0.42801	-0.30359
C	-2.84202	2.80727	0.18360
H	-0.97806	3.80189	0.46128
C	-3.47954	1.59334	-0.13314
H	-3.44365	3.70399	0.32420
C	-4.66586	-0.79794	-0.92940
H	-5.12746	-1.71947	-1.27825
C	-5.46830	0.33910	-0.69759
H	-6.54441	0.28148	-0.84511
C	4.88063	1.50059	-0.29622
H	5.47650	2.39073	-0.09882
C	5.46002	0.33704	-0.73174
H	6.53592	0.27799	-0.88043
C	4.64962	-0.78245	-1.01706
H	5.10558	-1.68974	-1.40811
C	3.28988	-0.74377	-0.82487
H	2.69004	-1.60491	-1.10324
C	-4.87978	1.51692	-0.31611
H	-5.46856	2.42025	-0.16229
C	-3.30612	-0.76030	-0.73655
H	-2.71487	-1.63924	-0.97248
N	-1.31868	0.48450	-0.09653
N	1.31208	0.48587	-0.13498
C	0.05636	-0.56814	2.05295
O	0.09647	-0.23694	3.14707

$[4\text{-CO}]^{0}$

Mn	0.00004	-1.10389	0.29082
C	-0.00546	-1.50944	-1.52820
C	1.23720	-2.34956	0.70581
C	-1.23571	-2.34761	0.71595
O	-0.00887	-1.71064	-2.65681
O	-1.96584	-3.18186	1.04129
O	1.96848	-3.18586	1.02315
C	0.71261	1.66549	0.15931
C	2.66267	0.41362	-0.34327
C	1.49422	2.83344	0.37186
C	-0.71328	1.66605	0.15808
C	3.48167	1.56539	-0.14475
C	2.84838	2.77977	0.25719
H	1.00688	3.76948	0.63157
C	-1.49372	2.83663	0.36019
H	3.46235	3.66256	0.43249
C	-2.66408	0.41324	-0.33941
C	-2.84781	2.78364	0.24468
H	-1.00539	3.77494	0.60959
C	-3.48218	1.56687	-0.14794
H	-3.46081	3.66878	0.41124
C	-4.65391	-0.80370	-1.03643
H	-5.09995	-1.72351	-1.41184
C	-5.45929	0.31690	-0.79749
H	-6.53311	0.26934	-0.96749
C	4.86632	1.48863	-0.36557
H	5.46391	2.38445	-0.19328
C	5.45748	0.31775	-0.80267
H	6.53121	0.27039	-0.97334
C	4.65120	-0.80084	-1.04797
H	5.09647	-1.71870	-1.42902
C	3.28612	-0.75611	-0.82767
H	2.68341	-1.62675	-1.07186
C	-4.86696	1.48996	-0.36795
H	-5.46367	2.38741	-0.20126

C	-3.28872	-0.75870	-0.81695
H	-2.68707	-1.63135	-1.05627
N	-1.31257	0.46140	-0.09774
N	1.31110	0.46146	-0.10206
C	0.00709	-0.52773	2.06521
O	0.01272	-0.17816	3.15682
